# Supplementary material for: Examining the reliability and validity of two versions of the Effort-Expenditure for Rewards Task (EEfRT)
Source: PLoS One. 2022 Jan 28;17(1):e0262902. doi: 10.1371/journal.pone.0262902 (PMC8797221; doi:10.1371/journal.pone.0262902)
Supplement: Attachment — Submitted filename: PONE-D-21-08512_R1_reviewer.pdf [file pone.0262902.s002.pdf]

PLOS ONE

Examining the reliability and validity of two versions of the Effort-Expenditure for Rewards Task (EEfRT)

--Manuscript Draft--

**Manuscript Number:**	PONE-D-21-08512R1
**Article Type:**	Research Article
**Full Title:**	Examining the reliability and validity of two versions of the Effort-Expenditure for Rewards Task (EEfRT)
**Short Title:**	Validation of two versions of the EEfRT
**Corresponding Author:**	Hanno Andreas Ohmann<br>Universität Hamburg: Universitat Hamburg<br>Hamburg, GERMANY
**Keywords:**	reliability; validity; EEfRT; Approach motivation; BAS
**Abstract:**	The Effort-Expenditure for Rewards Task (EEfRT) has gained validity evidence from several studies. However, various modifications have been applied to the original version, which have never been compared systematically. In Study 1, we tested 120 healthy participants to directly compare two versions of the EEfRT. In Study 2, we tested a larger sample of 394 healthy participants to further examine the original EEfRT. We replicated the split-half reliability of both task versions. However, self-reported personality traits (e.g., trait BAS) correlated with only some task performance parameters in Study 1, which did not replicate for the original EEfRT in Study 2. Our results indicate complex and sometimes inconsistent relations between different personality traits, task properties, and reward attributes.
**Order of Authors:**	Hanno Andreas Ohmann
	Niclas Kuper
	Jan Wacker
**Response to Reviewers:**	Response to Reviewer 1:<br>Reviewer #1: This study represents an important, relatively large scale study of the psychometrics and convergent and discriminant validity of alternate forms of the EEfRT, a task that is often modified with surprisingly little examination of the effects of those modifications. This report is an important one for anyone using the EEfRT to consider, and I offer a variety of suggestions to strengthen its contribution to the literature.<br>Thank You! Please note that line numbers in our response refer to the new “Manuscript” file (as line numbering is messed up within the tracked changes document.<br>INTRODUCTION<br><br>In the Hypotheses, the authors note that the reliability of the EEfRT appears promising from three previous studies; however, it would be helpful to note that based on their table, this is test-retest reliability specifically.<br>Thank you for raising this important issue. We clarified the types of reliability measured in previous studies as mentioned in the hypothesis (p. 11, l. 176-179).<br><br>In the paragraph spanning pp. 11-12, what were the actual correlations in the cited studies between EEfRT behavior and self-report measures? Having those relayed in the text would allow the reader to gauge immediately whether the authors’ assertions about the expected effect size were appropriate. In general, it would be appropriate to expect some shrinkage from published effect sizes, especially with a larger sample (which then has a smaller critical r value for a particular alpha level). Furthermore, Study 2’s Participants description seems to assume a smaller critical correlation without it being introduced here.<br>We fully agree and added the actual correlations to that paragraph (p. 11-12, l. 193-198). Indeed, we also expected some shrinkage from the published effect sizes. The smaller critical correlation expected in study 2 is partly based on the discrepancy found

between published correlations and the correlations found in our own lab. We clarified this issue in the methods section of study 2 (p. 19-20, l. 402-406).

METHOD (with references largely confined to Study 1)
To what extent do the rather rigorous exclusion criteria match those used in other studies with healthy samples?
Our exclusion criteria within study 1 are in line with previous studies conducted within our lab (i.e., Ohmann et al., 2018; Ohmann et al., 2020) and we believe those criteria are commonly used in the literature. The stricter criteria within study 2 are due to the clinical setting and the various molecular genetic hypotheses which are not part of the reported project.

Would changing the distribution of money from a randomly sampled two trials to 5% of the total winnings on the EEfRT constitute a modification to the overall procedure?
See below.

Nice job of using randomized task order to prevent order effects from influencing behavior. Why was the BART always in the middle of the task order?
Both versions of the EEfRT demand rather high motor-intense reactions. Thus, the BART (without any motor-intense reaction) has been used in the middle to avoid motoric fatigue of the subjects throughout the whole study. We adjusted the methods section to point out this issue (p. 14, l. 258-260).

Though the authors cite a couple of studies for the BART's automatic response procedure, it would seem that version of the task would compromise the motoric response component of the BART that might make it most comparable to the EEfRT. To what extent is that modified version of the BART consistent with the motor-intensive original version that requires key presses for each pump, both in terms of scores and correlations with external variables in theoretically expected ways?
The reactions to the BART should be seen as a different kind of reaction compared to the EEfRT, as the "pumps" of the original BART are not made under time pressure and each reaction could lead to the loss of the earned money within the specific trial (Lejuez et al., 2002). Thus, the task is not intended to measure physical effort. Instead, the BART (especially the automatic response procedure we used) measures impulsivity and the willingness of taking risks – which is the reason why we used this task. In this regard the automatic response version appears to be related to other measures in comparable fashion as the original version (for a comparison of both versions see Pleskac et al., 2008).

In the paragraph describing the modified EEfRT, I now see how the awarding of 5% of the total money earned might constitute a substantial modification compared to awarding the money won on two randomly selected trials. When money is awarded as a function of the proportion of (hard) trials completed, strategic concerns are minimized (which was an integral part of Zald and Treadway's reasoning for designing the task that way; see the middle of the second to last paragraph in the section describing the EEfRT in Treadway et al., 2009). However, when a flat 5% of the winnings are awarded, strategy plays a substantial role in what kinds of trials a person might choose to play. This discrepancy in extra compensation paradigms deserves explication.
Thank you for pointing out this important matter! We added a more critical review on this issue in the discussion (p. 41, l. 832-848).

Just to clarify, the maximum number of button presses across each of the 10 motoric ability trials was used as the MaxMot variable? Was this variable computed separately for each of the two iterations of this task?
Yes, the maximum number of button presses across each of the 10 motoric ability trials was used as the MaxMot variable and it was computed separately for each iteration of this task. As both versions of the EEfRT demand motor-intense reactions, we couldn`t

Powered by Editorial Manager® and ProduXion Manager® from Aries Systems Corporation

rule out any kind of motoric fatigue. Therefore, we added two iterations of the motoric ability task (one before each version of the EEfRT). However, the maximum number of button presses did not differ significantly between both iterations.

What response function was used in the GEEs? The analyses of hard task choices should use some kind of binomial-based response rather than one assuming a linear response.
Yes, we used a binomial-based response for the hard task choices. We added this information to the methods section (p. 18, l. 373-375).

The GEEs should not use an independent working correlation matrix; that will assume there are no correlations among cells, which is incorrect in a design featuring within-subjects factors. Treadway et al. (2009) used an unstructured correlation matrix to fully account for the vagaries of the relationships among cells, but one can argue that correlation matrix estimates too many parameters to be stable. An autoregressive or m-dependent matrix might be plausible, as might an exchangeable matrix if the authors really wanted to cut down on the parameters estimated. However, the dependence among observations should be accounted for in the working correlation matrix to ensure reasonable standard errors. The marginal means are consistent even if a working correlations is misspecified, but the standard errors are not (e.g., Hin & Wang, 2009). However, the overall standard errors are not so extreme as to vitiate finding meaningful results in this study (per Table 3).

GEEs provide unbiased estimates and appropriate robust standard errors even in the case that the working correlation matrix is misspecified. We emphasize this in the text (p. 18, l. 368-372). In line with your suggestions, we now report results using an exchangeable correlation matrix (Table 3). Results using this correlation matrix were highly similar to our original results.

Did the authors ensure equal distributions of low, medium, and high reward magnitudes across original EEfRT trials based on their binnings presented in the Data Analysis section?
The distribution of low, medium and high reward magnitudes is equal across the binnings of the original EEfRT trials.

RESULTS
If the split half reliability was calculated for the EEfRT's reliability, Cronbach's alpha (which is the average of all possible split halves) could also be calculated unless the halves were carefully balanced in some way. However, this is a relatively minor point.
Thank you for this suggestion. However, we intentionally chose the implemented split-half correlation over a measure of internal consistency. In particular, reliability was calculated by splitting by trial number (original task, with varying trial numbers per person) and block (modified task), yielding balanced test halves. We adjusted the method section to increase precision (p. 18, l. 363-365). Furthermore, we adjusted the report of reliabilities to show all relevant variables (Table 2)

How did the authors adjust raw EEfRT scores in Study 1 for motoric activity? Did they analyze some sort of residualized task performance score?
Yes, we residualized the task performance of the EEfRT (p. 21, l. 450-454). Within the GEEs we added the motoric abilities as an additional factor (MaxMot) to directly access its impact on task performance.

If I recall correctly, the β column in Table 3 should actually be a B column, as those coefficients are unstandardized.
Thank you for making us aware of this, we replaced all betas with Bs accordingly.

Figures 3-5 were really nicely done as a way to examine each participant's data along with the measures of central tendency and variability.
Thank You!

In Table 4, what adjustment was made for families of multiple comparisons? Some adjustment needs to be made given the exploratory nature of these analyses, which would likely alter the presentation of the results on p. 27. To maintain the presentation,

Powered by Editorial Manager® and ProduXion Manager® from Aries Systems Corporation

it might be reasonable to use one tabular asterisk for an uncorrected p < .05 and two tabular asterisks for a corrected p < .05.
Thank you for this suggestion. We implemented the suggested approach in Tables 4, 5, 6, 7, and 8. In particular, we Bonferroni-corrected the alpha level for each correlate (trait or strategy / motivation) separately (5 correlates each), yielding an adjusted alpha level of .01. Accordingly, we note in the respective table notes that * indicates p <.05 at the uncorrected significance level and ** indicates p <.01, corresponding to p <.05 at an alpha level corrected for multiple comparisons for each correlate.

In the first paragraph of the Secondary Analyses section, I would argue that these correlations are even moderate given that they ostensibly represent alternate versions of the same task. I also did not see the values of the correlations for the various reward magnitudes, only the reward probabilities reported in Figure 7 (which is a lovely visualization of the relevant data).
We agree and added an additional table (Table 6) containing all relevant correlations between task parameters of the two tasks.

Tables 6 and 7 need corrections for multiple comparisons as well.
Thank you for making us aware of this, we implemented this correction as described above.

DISCUSSION

I believe the authors oversell substantially the comparability of the two versions of the EEfRT. Considerations of effect size are paramount in the first paragraph (e.g., difference between each form’s internal consistency and alternate forms’ correlations).
We adjusted the discussion and added a more critical view on this issue (p. 37, l. 741-746).

The discussion of self-report correlations may be a discussion of false positive errors given the lack of replicability of most of them across Studies 1 and 2 and the lack of corrections for multiple comparisons. The authors nod to this near the end of the second paragraph of the Discussion’s first section, but they can check how well their findings would survive correction for multiple comparisons.
We adjusted the discussion and added a more critical view on this issue based on the correction for multiple testing (p. 38, l. 761-767).

I can appreciate how the authors contextualize the size of their adaptation of extra compensation allocation compared to other EEfRT adaptations. However, I think they may underestimate how that changes the (lack of) strategic considerations in the original EEfRT, which may explain some of the low correlations across EEfRT forms.
We adjusted the discussion and added a more critical view on this issue (p. 41, l. 832-848).

Good note that floor and ceiling effects in the original EEfRT might limit the size of the validity coefficients with the modified EEfRT.
Thank You!

MINOR POINTS

Many paragraphs span multiple pages. These must be broken so that they occupy ½ to ¾ of a single manuscript page so they contain a single idea and its elaborations.
We tried to break paragraphs wherever possible and hope that the Ms is now easier to read.

REFERENCES FOR THIS REVIEW

Hin, L.-Y., & Wang, Y.-G. (2009). Working-correlation-structure identification in generalized estimating equations. Statistics in Medicine, 28, 642-658. https://doi.org/10.1002/sim.3489

References for this response
Lejuez CW, Richards JB, Read JP, Kahler CW, Ramsey SE, Stuart GL, et al.

Evaluation of a behavioral measure of risk taking: The balloon analogue risk task (BART). J Exp Psychol Appl. 2002; 8(2): 75–84. doi: 10.1037/1076-898X.8.2.75
Ohmann HA, Kuper N, Wacker J. Left frontal anodal tDCS increases approach motivation depending on reward attributes. Neuropsychologia. 2018; 119: 417–423. doi: 10.1016/j.neuropsychologia.2018.09.002
Ohmann HA, Kuper N, Wacker J. A low dosage of the dopamine D2-receptor antagonist sulpiride affects effort allocation for reward regardless of trait extraversion. Personal Neurosci. 2020; 3: E7. doi: 10.1017/pen.2020.7
Pleskac TJ, Wallsten TS, Wang P, Lejuez CW. Development of an automatic response mode to improve the clinical utility of sequential risk-taking tasks. Exp Clin Psychopharmacol. 2008; 16(6): 555–564. doi: 10.1037/a0014245

Response to Reviewer 2:
Reviewer #2: Generally a well done paper. It is a useful set of results about a fairly widely used measure. It is also quite informative to show that two versions of what is ostensibly the same measure, may actually have fairly modest correlations.
Thank you! Please note that line numbers in our response refer to the new “Manuscript” file (as line numbering is messed up within the tracked changes document.

In the discussion, I think the authors should explicitly mention all the personality traits that are not related to EEfRT, such as the UPPS. Although they do have some discussion of what it means that the behavioral measure is not really related to some important reward related measures of depression, I also think that they should provide more discussion of what it means that this behavioral measure is only minimally correlated with personality variables that have been shown to be related to depression, which the EEfRT is claimed to help measure.

We agree and now explicitly mention these traits in the discussion (p. 38, l. 755-760). Furthermore, we expanded the discussion of the minimal correlations between behavioral measures and personality variables: We think that these correlations are still too exploratory in nature to be interpreted in terms of measurement of depression (via the EEfRT) and there are too many other possible factors impacting these correlations (p. 40, l. 814-826).
I have a number of minor comments.

As they note, the measure was developed for use in distinguishing clinical samples from normals, so it might be problematic to only collect data on a young, healthy sample. They do note at one point, the problem they had with ceiling and floor effects. This might be a general problem with the EEfRT.
Thank you for mentioning this issue. We adjusted the discussion (p. 42, l. 857-860).

They aren't always as explicit as they should be in stating that some of their measures, such as the TEPS and the UPPS are essentially not correlated with the EEfRT in a normal sample.
We adjusted the discussion as mentioned above (p. 38, l. 755-760).

Line 49. Culminated would be a better word choice than accumulated.
We corrected the term, thank you.
Line 75. Specifically would be a better word choice than Precisely.
We corrected the term, thank you.
Line 86. Say that one would expect relatively specific associations, but then don't give any idea of what those are.
We added an explanation to this section (p. 4-5, l. 89-92).
Line 108. "During task performance", not "while task performance"
We corrected the term, thank you.

Line 279. "Then, after a ready – screen (1s, C) main screen for the trial showing a…" There is punctuation or words missing. Doesn’t' read properly.
We increased the precision of the task description (p. 15, l. 289-291).

Line 324 starting with "Furthermore, we…" Something is wrong with this sentence.

Powered by Editorial Manager® and ProduXion Manager® from Aries Systems Corporation

	We corrected that sentence (p. 17, l. 334).<br><br>Line 387. "criterion" not "criterium"<br>We corrected the term, thank you.<br><br>Line 550. A correlation with a difference score is hard to interpret. Would be nice to explicitly state what it means.<br>We added an explanation to that section (p. 28 l. 576-579).<br><br>Line 560. So basically almost none of the personality variables are correlated with either version of the EEfRT in a normal sample?<br>That’s correct. Overall, we increased precision of our manuscript regarding this issue according to your valuable input!
**Additional Information:**	
**Question**	**Response**
**Financial Disclosure**<br><br>Enter a financial disclosure statement that describes the sources of funding for the work included in this submission. Review the submission guidelines for detailed requirements. View published research articles from *PLOS ONE* for specific examples.<br><br>This statement is required for submission and **will appear in the published article** if the submission is accepted. Please make sure it is accurate.<br><br>**Unfunded studies**<br>Enter: *The author(s) received no specific funding for this work.*<br><br>**Funded studies**<br>Enter a statement with the following details:<br>• Initials of the authors who received each award<br>• Grant numbers awarded to each author<br>• The full name of each funder<br>• URL of each funder website<br>• Did the sponsors or funders play any role in the study design, data collection and analysis, decision to publish, or preparation of the manuscript?<br>• **NO** - Include this sentence at the end of your statement: *The funders had no role in study design, data collection and analysis, decision to publish, or preparation of the manuscript.*<br>• **YES** - Specify the role(s) played.<br><br>* typeset	The author(s) received no specific funding for this work.

**Competing Interests**<br><br>Use the instructions below to enter a competing interest statement for this submission. On behalf of all authors, disclose any competing interests that could be perceived to bias this work—acknowledging all financial support and any other relevant financial or non-financial competing interests.<br><br>This statement is required for submission and **will appear in the published article** if the submission is accepted. Please make sure it is accurate and that any funding sources listed in your Funding Information later in the submission form are also declared in your Financial Disclosure statement.<br><br>View published research articles from *PLOS ONE* for specific examples.<br><br>**NO authors have competing interests**<br><br>Enter: *The authors have declared that no competing interests exist.*<br><br>**Authors with competing interests**<br><br>Enter competing interest details beginning with this statement:<br><br>*I have read the journal's policy and the authors of this manuscript have the following competing interests: [insert competing interests here]*<br><br>* typeset	The authors have declared that no competing interests exist.
**Ethics Statement**<br><br>Enter an ethics statement for this submission. This statement is required if the study involved:<br><br>• Human participants<br>• Human specimens or tissue<br>• Vertebrate animals or cephalopods	Study 1 has been approved by the local Ethics Committee of the University of Hamburg in written form. Study 2 has been approved by the local Ethics Committee of the Medical Chamber of Hamburg in written form.<br><br>Study 1: Participants gave their informed consent in written form via E-Mail and then received an individual link to the first part of the study. After arriving at our lab, participants personally signed the informed consent, which they sent before via E-Mail.<br><br>Study 2: After arriving at the lab the participants gave their informed consent in written

• Vertebrate embryos or tissues<br>• Field research<br><br>Write "N/A" if the submission does not require an ethics statement.<br><br>General guidance is provided below. Consult the submission guidelines for detailed instructions. **Make sure that all information entered here is included in the Methods section of the manuscript.**	form.

Powered by Editorial Manager® and ProduXion Manager® from Aries Systems Corporation

**Format for specific study types**<br><br>**Human Subject Research (involving human participants and/or tissue)**<br>• Give the name of the institutional review board or ethics committee that approved the study<br>• Include the approval number and/or a statement indicating approval of this research<br>• Indicate the form of consent obtained (written/oral) or the reason that consent was not obtained (e.g. the data were analyzed anonymously)<br><br>**Animal Research (involving vertebrate animals, embryos or tissues)**<br>• Provide the name of the Institutional Animal Care and Use Committee (IACUC) or other relevant ethics board that reviewed the study protocol, and indicate whether they approved this research or granted a formal waiver of ethical approval<br>• Include an approval number if one was obtained<br>• If the study involved *non-human primates*, add *additional details* about animal welfare and steps taken to ameliorate suffering<br>• If anesthesia, euthanasia, or any kind of animal sacrifice is part of the study, include briefly which substances and/or methods were applied<br><br>**Field Research**<br><br>Include the following details if this study involves the collection of plant, animal, or other materials from a natural setting:<br>• Field permit number<br>• Name of the institution or relevant body that granted permission	
**Data Availability**<br><br>Authors are required to make all data underlying the findings described fully available, without restriction, and from the time of publication. PLOS allows rare exceptions to address legal and ethical concerns. See the PLOS Data Policy and FAQ for detailed information.	Yes - all data are fully available without restriction

A Data Availability Statement describing where the data can be found is required at submission. Your answers to this question constitute the Data Availability Statement and **will be published in the article**, if accepted.<br><br>**Important:** Stating 'data available on request from the author' is not sufficient. If your data are only available upon request, select 'No' for the first question and explain your exceptional situation in the text box.<br><br>Do the authors confirm that all data underlying the findings described in their manuscript are fully available without restriction?	
**Describe where the data may be found in full sentences. If you are copying our sample text, replace any instances of XXX with the appropriate details.**<br><br>• If the data are **held or will be held in a public repository**, include URLs, accession numbers or DOIs. If this information will only be available after acceptance, indicate this by ticking the box below. For example: *All XXX files are available from the XXX database (accession number(s) XXX, XXX.*).<br>• If the data are all contained **within the manuscript and/or Supporting Information files**, enter the following: *All relevant data are within the manuscript and its Supporting Information files.*<br>• If neither of these applies but you are able to provide **details of access elsewhere**, with or without limitations, please do so. For example:<br><br>*Data cannot be shared publicly because of [XXX]. Data are available from the XXX Institutional Data Access / Ethics Committee (contact via XXX) for researchers who meet the criteria for access to confidential data.*<br><br>*The data underlying the results presented in the study are available from (include the name of the third party*	All data and codes are shared openly at our OSF-page: https://osf.io/35k2w/?view_only=1f315859028c471787a96e153f5ac43a.

Powered by Editorial Manager® and ProduXion Manager® from Aries Systems Corporation

and contact information or URL).<br>• This text is appropriate if the data are owned by a third party and authors do not have permission to share the data.<br><br>* typeset	
Additional data availability information:	

Powered by Editorial Manager® and ProduXion Manager® from Aries Systems Corporation

Manuscript Click here to access/download;Manuscript;Manuscript.docx

Examining the reliability and validity of two versions of the Effort-Expenditure for Rewards Task (EEfRT)

Hanno Andreas Ohmann[1*], Niclas Kuper[2], & Jan Wacker[1]

Universität Hamburg

Short title: Validation of two versions of the EEfRT

[1] Faculty of Psychology and Human Movement Science, Universität Hamburg, Hamburg, Germany

[2] Faculty of Psychology and Sports Science, Universität Bielefeld, Bielefeld, Germany

*Corresponding Author

hanno_ohmann@hotmail.com (HAO)

Keywords: Reliability; Validity; EEfRT; Approach Motivation; BAS

Abstract

The Effort-Expenditure for Rewards Task (EEfRT) has gained validity evidence from several studies. However, various modifications have been applied to the original version, which have never been compared systematically. In Study 1, we tested 120 healthy participants to directly compare two versions of the EEfRT. In Study 2, we tested a larger sample of 394 healthy participants to further examine the original EEfRT. We replicated the split-half reliability of both task versions. However, self-reported personality traits (e.g., trait BAS) correlated with only some task performance parameters in Study 1, which did not replicate for the original EEfRT in Study 2. Our results indicate complex and sometimes inconsistent relations between different personality traits, task properties, and reward attributes.

1. Introduction

Harmon-Jones et al. [1] (p. 291) define approach motivation as “the impulse to go toward”, which is based on internal state or trait - related processes and initiates behavior which is often (but not necessarily) associated with reaching specific goals. Approach motivation can therefore be seen as one of the main drivers of human behavior [2, 3]. The field of personality research related to approach motivation is often linked to Gray`s model of personality, which culminated in his Reinforcement Sensitivity Theory (RST) [4, 5]. A variety of self-report measures are available for the assessment of personality traits related to sensitivity to rewards and punishment, ranging from the commonly used BIS / BAS scales [6] to the Temporal Experience of Pleasure Scale (TEPS) [7] measuring anticipatory pleasure and consummatory pleasure.

However, linking self-reported personality traits to behavioral measurements often leads to rather small correlations [8, 9]. Dang et al. [10] suggest that this might be a result of the poor reliability of many behavioral measures and the different response processes of these two types of measurement. Moreover, multiple behavioral tasks to measure approach motivation have been developed in a post-hoc manner and / or are based on animal models [11]. A majority of these tasks utilizes physical effort, which participants have to invest to gain rewards. Various kinds of physical efforts have been utilized. For example, hand-grip tasks assess willingness to expend effort by force exerted on the grip [12–14]. Such tasks are often adapted to a model based on effort discounting, which allows one to measure the extent to which the need for effort reduces preference for a given reward [15–17]. Similar tasks use button or lever pressing in a progressive ratio format [18–20]. Unfortunately, the majority of these tasks lack a comprehensive test of their reliability and validity. Comparing the psychometric properties of five different effort-based decision-making tasks in a clinical

sample (schizophrenic patients) and a healthy control sample, the Effort Expenditure for Rewards Task (EEfRT) [21] exceeded the reliability of four other tasks [14, 22], making it a promising tool to investigate approach motivation.

The EEfRT is based on a concurrent choice paradigm developed by Salamone et al. [11] to explore effort-based decision making in rodents. The original EEfRT measures individual differences in human reward motivation by having participants decide between high cost/high reward (hard task, many clicks needed) and low cost/low reward (easy task, few clicks needed) behavioral options. The tendency to choose the hard task rather than the easy task has been shown to be associated with higher levels of approach motivation, as measured e.g., via personality trait questionnaires [23]. Specifically, trait BAS and trait anticipatory pleasure correlated positively with the percentage of hard-task-choices in trials with a low probability of rewards attainment in the original EEfRT. This indicates that higher approach motivation as measured by trait questionnaires is related to concrete behavior directed at gaining rewards in the EEfRT. Recently, these findings were replicated using a modified version of the EEfRT, showing that trait extraversion and trait BAS correlated positively with the mean number of clicks participants exerted in trials with a low probability of reward attainment [24]. However, Horan et al. [22], Anand et al. [25], as well as Kaack et al. [26] were not able to replicate these correlations using the original EEfRT, raising questions about the existence and magnitude of such associations.

According to Smillie [27], trait behavioral activation system (BAS) sensitivity should be predominantly related to reward sensitivity, while trait behavioral inhibition system (BIS) sensitivity should be predominantly related to punishment sensitivity. One would thus expect associations between behavior in the EEfRT and these personality traits. In particular, trait BAS should be associated with increased task performance (due to greater sensitivity to rewards) and trait BIS with decreased task performance (due to higher sensitivity to the

aversive quality of effort expenditure). The EEfRT has gained further support for its validity from various studies (see Table 1). For instance, healthy participants' preference for the hard task has been shown to correlate with lower scores on negative affect, depressive symptoms, and anhedonia [21]. Furthermore, left frontal anodal transcranial direct current stimulation (tDCS) over the dorsolateral prefrontal cortex increased participants' willingness to choose the hard task depending on reward attributes [28], which is in line with models associating left frontal brain activity with approach motivation [29, 30]. Wardle et al. [31] were the first to show that the EEfRT is also sensitive to pharmacological manipulation of dopamine (DA), as d-amphetamine increased participants' overall effort allocation. Furthermore, a low dosage of the D2 receptor blocker sulpiride, which is e.g., used in patients with depression and is believed to increase approach motivation, decreased participants' willingness to exert clicks in a modified version of the EEfRT [24].

Additional evidence for the validity of the EEfRT comes from patients suffering from impaired approach motivation: Patients with schizophrenia [32–34], first-episode psychosis [35], depression [36, 37], and autism [38] were less willing to choose the hard task as compared to healthy controls. Furthermore, the number of hard-task-choices was found to be negatively correlated with the severity of anhedonic symptoms in patients with schizophrenia [33] as well as in patients with depression [37]. Nguyen et al. [39] found in a large healthy sample of parents and their children that symptoms of psychopathology correlated with reduced effort allocation within the EEfRT. [new para] The EEfRT has also gained evidence on a neurophysiological level, as it has been shown to be related to left-frontal cortical asymmetry in the resting state as well as during task performance [26, 40], which is believed to be a neural signature of approach motivation. Moreover, Huang et al. [41] found that the percentage of hard-task-choices was directly linked to the activity of the NAcc, which is a key structure of the human reward circuit, in both patients with schizophrenia and healthy

participants. Overall, the literature thus shows intriguing support for the validity of the EEfRT.

However, there are also various limiting aspects (see Table 1). First, the number of studies reporting a significant link between the behavioral measurements within the EEfRT and self-reported personality traits related to approach motivation is still small, although many studies refer to this link as validity evidence of the EEfRT. Second, the number of participants in studies which used the EEfRT has often been relatively small, resulting in low statistical power to detect effects sizes that can be expected in individual difference research [42]. Together with concerns about the replicability of psychological findings in general [43] as well as literatures relevant to this manuscript (e.g., trait approach motivation – frontal asymmetry link) [44], this highlights the risk of false positive results in previous studies. Third, the original EEfRT has been shown to be partly related to individual strategic behavior, which is not related to participants' actual approach motivation [28]. The modified version of the EEfRT [24] seeks to eliminate this limitation of the original task, but requires additional data to document its reliability and validity. Fourth, seemingly small differences in task properties and administration could have a great impact on task behavior. Despite this, several studies already modified the original EEfRT to fit different experimental settings (see Table 1).

Table 1. An overview of selected studies using the EEfRT: Modifications, reliability, and validity (chronological order).

Study	N	Main dependent variable(s)	Main modifications applied to the original task	Modifications tested against original?	Reliability reported?	Validity test
Treadway et al. 2009 [21]	61	HTC	-	-	No	Self-report measures (Anhedonia, depression)
Wardle et al. 2011 [31]	17	HTC	Repeated-measure-design	No	No	Effects of d-amphetamine
Damiano et al. 2012 [38]	58	HTC, response change	Removed time-limit for task choice	No	No	Patients with ASD (n= 20) VS healthy controls (n = 38)
Wardle et al. 2012 [45]	23	HTC, response speed	Practice session, repeated measure	No	No	Effects of caffeine
Fervaha et al. 2013 [32]	32	HTC	Individual calibration of required clicks	No	No	Patients with SCZ (n= 16) VS healthy controls (n = 16)
Barch et al. 2014 [33]	98	HTC	Removed low probability trials	No	No	Patients with SCZ (n= 59) VS healthy controls (n = 39); self-report measures (anhedonia, depression)
Yang et al. 2014 [37]<br>- study 1	99	HTC	Reduced number of required clicks; fixed the possible monetary rewards	No	No	high BDI-score (n =43) VS low BDI score (n =56); self-report measures (anhedonia; pleasure)
- study 2	87	HTC	Reduced number of required clicks; fixed the possible monetary rewards	No	No	Patients with MDD (n = 41) VS remitted MDD (n= 41)

Note. ASD = Autism spectrum disorder; BAS = Behavioral Activation System; BIS= Behavioral Inhibition System BDI = Beck Depression Inventory; BMI = Body-mass-index; HTC = Hard-task-choices; MDD = major depressive disorder; SCZ = schizophrenia

Table 1 (Continuation). An overview of selected studies using the EEfRT: Modifications, reliability, and validity (chronological order).

**Study**	**N**	**Main dependent variable(s)**	**Main modifications applied to the original task**	**Modifications tested against original?**	**Reliability reported?**	**Validity test**
Hughes et al. 2015 [46]	51	HTC	Paid 10% of total winnings	No	No	Association with frontal asymmetry (resting state)
Geaney et al. 2015 [23]	97	HTC	Mood induction; paid 10% of total winnings	No	No	Self-report measures (anhedonia, pleasure, BAS)
Gilman et al. 2015 [47]	50	HTC; reaction time	Pictures of peers (social influence)	No	No	Effects of different social influence conditions
Reddy et al. 2015 [14]	134	Reward sensitivity	Repeated-measure-design; individual calibration of required clicks; no low probability trials	No	**Yes (retest)**	Patients with SCZ (n= 94) VS healthy controls (n = 40); comparison with four other tasks
Horan et al. 2015 [22]	134	Reward sensitivity	Repeated-measure-design; individual calibration of required clicks; no low probability trials	No	**Yes (retest)**	Self-report measures (SCZ symptoms; motivation; BIS/BAS)
Hughes et al. 2017 [48]	128	HTC	Repeated-measure-design; shorter task selection (3s)	No	No	Abstinent smokers (n = 61) VS former smokers (n = 67); self-reported reward enjoyment
Johnson et al. 2017 [49]	50	HTC	Fixed probability (50%), three bonus trials added (no effort needed)	No	No	Patients with remitted bipolar disorder; self-reported life ambitions
Lopez-Gamundi & Wardle 2018 [51]	60	HTC	Cognitive effort version	**Yes**	No	Comparison of cognitive VS original version

Note. ASD = Autism spectrum disorder; BAS = Behavioral Activation System; BIS= Behavioral Inhibition System BDI = Beck Depression Inventory; BMI = Body-mass-index; HTC = Hard-task-choices; MDD = major depressive disorder; SCZ = schizophrenia

141 **Table 1 (Continuation). An overview of selected studies using the EEfRT: Modifications, reliability, and validity (chronological order).**

Study	N	Main dependent variable(s)	Main modifications applied to the original task	Modifications tested against original?	Reliability reported?	Validity test
Ohmann et al. 2018 [28]	60	HTC	Repeated-measure-design; paid 5% of the total winnings	No	No	Effects of anodal tDCS stimulation
Racine et al. 2019 [50]	63	HTC	Food portions instead of monetary reward	No	No	Comparison of participants with different degree of binge-eating symptoms; BMI
Nguyen et al. 2019 [39]	2259	Reward sensitivity	No probabilities of reward attainment for kids	No	No	Comparison of parents (n= 1044) and children (n= 1215) for psychopathic symptoms
Byrne & Ghaiumy Anaraky 2019 [52]	94	HTC	Addition of “loose” trials; temporal delay after easy trials	No	No	Comparison of older (n= 46) and younger adults (n= 48)
Ohmann et al. 2020 [24]	203	Button presses	No task selection, instead increased potential monetary gains with each click	No	**Yes (split-half)**	Effects of sulpiride; self-report measures (Big Five; BIS/BAS)
Kaack et al. 2020 [26]	49	HTC	Additional “offer”- screen (5s) prior to choice screen; best performing student would receive $100 grocery store voucher	No	No	Associations with frontal asymmetry (resting and task states); self-report measures (BIS/BAS)

142 *Note.* ASD = Autism spectrum disorder; BAS = Behavioral Activation System; BIS= Behavioral Inhibition System BDI = Beck Depression Inventory; BMI =
143 Body-mass-index; HTC = Hard-task-choices; MDD = major depressive disorder; SCZ = schizophrenia

Just to name a few examples: Yang et al. [37] reduced the number of required clicks and fixed the possible monetary rewards to reduce the complexity of the task for depressive patients. This design was also used by Huang et al. [41] to make the EEfRT suitable for functional brain imaging. Barch et al. [33] completely removed the low probability of reward attainment category, Damiano et al. [38] removed the time limit when participants select either the easy or the hard task, Fervaha et al. [32] calculated an individual number of required clicks before the actual task based on motoric abilities, and Byrne & Ghaiumy Anaraky [52] introduced "loss trials", in which choosing the easy task leads to potentially higher monetary loss compared to the hard task. Other authors exchanged the monetary rewards of the EEfRT, e.g., by using food portions in a study with patients suffering from binge-eating [50]. A different study added a social influence aspect while participants chose between the easy and hard task of the EEfRT by adding pictures of "peers" and their respective choices [47]. Despite these various modifications applied to the EEfRT so far, surprisingly little is known about the effect of such modifications as almost no study directly compared different versions of the EEfRT within one experimental design. A commendable exception is a study by Lopez-Gamundi & Wardle [51], who compared the original EEfRT to a modification which uses cognitive effort (set-switching-task; C-EEfRT) instead of the physical effort within the original task (clicks). Although participants perceived the C-EEfRT as more difficult, participants did choose the hard task more often compared to the original EEfRT. Furthermore, the relationship between the effort allocation within both task versions was only moderate, indicating distinct processes for both kinds of effort when participants decide to allocate effort to gain a possible reward.

1.1 Present study

Bearing these limitations in mind, we here seek to analyze the reliability and validity of the original EEfRT and a modified EEfRT in two different study designs and try to deepen the understanding of the link between self-reported personality traits and behavioral task measures. Study 1 aims at directly comparing the validity and reliability of two versions of

the EEfRT. Study 2 aims to replicate the reliability and validity of the original EEfRT by making use of a large sample to further increase statistical power.

1.1.1 Reliability of the EEfRT

Based on the promising results regarding the retest-reliability found in previous studies for the original EEfRT [14, 22] and likewise promising results for the split-half reliability of the modified EEfRT [24], we expected both versions of the task to show overall good (Rel > .80) split-half reliability in both studies. We further examined the internal consistency of all questionnaire measures used in both studies.

1.1.2 Validity of Basic Task Variables

In line with previous research, we expected the reward attributes (reward magnitude and probability of reward attainment) to be positive predictors of the percentage of hard-task-choices (original EEfRT) and mean number of clicks (modified EEfRT), whereas we expected trial number (i.e., an indicator of fatigue) to be a negative predictor of the percentage of hard-task-choices (original EEfRT) and mean number of clicks (modified EEfRT). GEE models (generalized estimating equations) [53, 54] have been the main analysis strategy for the examination of basic task variables in previous work on the EEfRT [21, 23, 24]. Therefore, we also applied GEE models to test for the effects of the above-mentioned basic task variables.

1.1.3 Personality Correlations

As Gignac & Szodorai [42] stated, correlations of $r = .30$ should be considered as rather large correlations in the field of individual differences. Applying these standards, previous studies using the original EEfRT [23] as well as the modified version of the EEfRT [24] found medium to large correlations for the percentage of hard-task-choices (original EEfRT) and the mean number of clicks (modified EEfRT) in trials with low probability of reward attainment with trait BAS (*r* ranging from .212 to .361) and trait anticipatory pleasure

(r = .251). We seek to replicate these correlations in both studies. Furthermore, as some studies using the EEfRT focused on the impact of reward magnitude, as well as the differences between low and high reward trials [14, 22], indicating "reward sensitivity", we exploratorily analyzed the correlations between traits with the percentage of hard-task-choices (original EEfRT) and mean number of clicks (modified EEfRT) depending on reward magnitude. Therefore, we examined the correlations between trait measures and task performance in all probability of reward attainment categories as well as in all reward magnitude categories and difference scores between these trial categories in an exploratory fashion. To evaluate the discriminant validity of both tasks, we further tested for the associations with distinct constructs, which we expected to not correlate with effort allocation on both tasks - namely risk-taking (behavioral measure) and impulsivity (self-reported trait measure).

1.1.4 Secondary Analysis

As Study 1 includes both versions of the EEfRT in one experimental design, we seek to estimate the correlations between both task versions in an exploratory fashion. As there are no previous studies to base our hypotheses on, we only hypothesize a significant positive correlation between both versions, as both tasks should measure the same construct: approach motivation. We further analyzed participants' self-reported strategy usage and motivation and their linkage to task performance in Study 1 to further test the validity of both task versions.

2. Methods

2.1 Study 1

2.1.1 Participants

We recruited physically and psychologically healthy participants (78.3% female) aged 18 – 35 (M = 24.97; SD = 4.14) using online notice boards and flyers at a local university. Out of 125 recruited participants, 5 had to be excluded for different reasons (1 participant was not

able to understand the instructions; 2 participants did not understand the task; 2 participants had missing values due to technical failure). Thus, the final sample consisted of 120 participants. In line with previous studies [23, 24], we expected correlations around $\rho = .30$ (i.e., large correlations according to Gignac & Szodorai [42]). As intended, statistical power was therefore >.80 (exact 1-β = .92) to detect correlations of $\rho = .30$ ($\alpha = .05$). Participants received monetary compensation (10€ per hour) and were told that they could gain additional money based on their collected rewards from both versions of the EEfRT (5% of the virtually collected money) and the BART (one cent per 4 pumps in successful trials) which was paid to participants at the end of the study. The authors assert that all procedures contributing to this work comply with the ethical standards of the relevant national and institutional committees on human experimentation and with the Helsinki Declaration of 1975, as revised in 2008. The study has been approved by the local Ethics Committee of the University of Hamburg. Exclusion criteria comprised the intake of any kind of prescribed medication over the last three months, the consumption of illegal drugs over the last four weeks, neurological or medical conditions, and the presence of any mental disorders (in particular affective, somatoform, psychotic, anxiety, eating, and adaptive disorders, as well as substance use disorders).

2.1.2 Randomization

As we intended to compare both versions of the EEfRT (original / modified) and their relation to measures of personality traits and risk-taking behavior, we randomized the order of both tasks in a counterbalanced fashion to ensure that the order of both tasks did not influence effort allocation. Participants were randomly assigned to one of both conditions at the start of part two of our study.

2.1.3 Procedure

Participants who fit the inclusion criteria were provided with information about the study via email. Participants gave their informed consent in written form via email and then

received an individual link to the first part of the study. The first part of the study comprised an online-survey lasting about 30 minutes, in which participants filled out a series of questionnaires, including demographic information and German versions of the BIS/BAS scales [6, 55], the TEPS [7], and the UPPS [56, 57]. Participants who completed the first part of the study were invited into our lab. After arriving at our lab, participants personally signed the informed consent which they sent before via E-Mail. Afterwards, the participants completed a series of computer tasks lasting about 60 minutes. All participants started by completing a test of their motoric abilities. Participants then completed either the original or the modified version of the EEfRT according to the assigned condition. Afterwards, they completed the Balloon Analogue Risk Task (BART) [58, 59]. The BART was always completed in the middle of the study to avoid motoric fatigue of the participants throughout the whole study. The BART was followed by another test of motoric abilities and the complementary version of the EEfRT, which participants did not complete before. Finally, participants completed a small set of questions, including questions about aspects that might have influenced their effort allocation within each version of the task (reward magnitude, probability of reward, fatigue, resting one's fingers) and their motivation to earn additional money throughout the whole study using five-point Likert-scales ranging from "not at all" (1) to "a lot" (5).

2.1.4 Original EEfRT

We used a translated (German) version of the EEfRT [21], which was programmed using Presentation software 17.1 (Neurobehavioral Systems Inc, San Francisco). Every participant completed one 15–minute block of the EEfRT with their dominant hand. Participants were instructed to win as much virtual money as possible throughout the block. In short, participants need to choose between an easy, low-reward task and a hard, high-reward task in every trial (see Fig 1 for a schematic illustration). The reward for the easy task is fixed to 1 € while the reward for the hard task is variable (ranging between 1.21 € and 4.30 €). To

further manipulate the value of each reward, the probability of reward attainment also varies [either 12% (low), 50% (medium) or 88% (high)], which is presented at the start of each trial alongside the reward values. The easy task requires participants to press the space button 30 times (“clicks”) in 7 seconds with their index finger. The hard task requires participants to press the space button 100 times (“clicks”) in 21 seconds with their pinkie finger. While pressing the spacebar, a visually presented white bar gradually fills up with red color. After each trial, the participants are informed about the amount of money they won during the trial. The order of trials, as well as the probability of reward attainment and the reward magnitudes are not randomized between participants, but pre-assigned for each trial. This is done to rule out random feedback differences between participants.

Fig 1. Schematic illustration of one trial of the original EEfRT.

A fixation cross (1s, A) is followed by a screen showing probability of reward attainment and reward magnitude for the easy and the hard task (B), lasting until the participant made a choice which task to complete but no longer than 5s. Then, after presentation of a ready – screen (1s, C) the main screen for the trial showing a red bar that fills with each button press is presented until the task is completed or until the trial time is over (D). Finally, task completion is signaled (2s, E) and a feedback screen shows the amount of money won (2s, F).

2.1.5 BART

To test participants` risk-taking behavior, we also assessed the BART [58, 59]. In this task, participants are presented a picture of a balloon, and instructed to inflate this balloon. Inflating the balloon increases the size of the balloon on screen and the associated reward. However, overinflating the balloon would result in the balloon bursting accompanied by an aversive auditory sound. Bursting of the balloon causes the participant to lose the entire reward of that trial. Each of 30 balloons have a different predetermined bursting point, on a

scale of 1 to 128 (pumps). Participants are instructed that the average number of inflations that causes the balloon to burst is 64 and that they would gain one Cent per 4 pumps, only in successful trials (balloon did not burst). In line with previous studies, we used the automatic response procedure of the BART [59, 60], in which the participants could immediately select the intended number of pumps for that specific trial and receive immediate feedback as they watch the balloon inflating. Also, risk taking scores were calculated as the mean of the number of pumps across all balloons regardless of the burst event [59], unlike in the original BART [58].

2.1.6 Modified EEfRT

Additionally, we used a modified version of the original EEfRT [21], which was programmed using Presentation software 17.1 (Neurobehavioral Systems Inc, San Francisco) and has been used in one previous study [24]. Ohmann et al. [28] found that using the original EEfRT comes with a major downside: At least some participants understand that choosing the hard task is often lowering the possible overall monetary gain as the hard task takes almost 3 times as long as the easy task and the overall duration of the task is fixed. Hence, at least some participants' choices are partly based on a strategic decision and less on approach motivation per se. To overcome this downside, the original EEfRT was modified. First, the number of trials (2 blocks x 15 trials = 30 trials) and the duration of each trial (= 20 seconds) was fixed. Participants used their dominant hand for both blocks in the present study. Second, the original choice-paradigm was changed. Participants no longer choose between an easy and a hard task. As in the original task, the value of each reward varies, and participants are informed about this at the start of each trial. But instead of presenting specific reward magnitudes, participants are now presented with a reward magnitude per click (1 /2 / 3 / 4 / 5 cents per click). Thus, participants are able to increase the total possible monetary gain in each trial with each click. In accordance with the original task design, the probability of reward attainment also varied [either 12% (low), 50% (medium) or 88% (high)], which is

presented at the start of each trial alongside the reward value per click. Participants were instructed to win as much virtual money as possible throughout the task, however they were free to choose the amount of effort they exerted in each trial. Critically, the only way to increase the possible monetary gain is to increase the number of clicks in each trial. The task itself is designed to be close to the original EEfRT but comes with some modifications to prevent the use of strategies (see Fig 2). While pressing the spacebar, a visually presented red bar gradually grows. A scale (€) was implemented, so that the participants can always see how much their button-presses ("clicks") increase their possible monetary gain. Furthermore, the information on the reward magnitude per click and the probability of reward attainment is presented throughout the whole trial alongside a countdown (20 seconds) to increase participants' awareness of these parameters. After each trial, participants are informed about the amount of money they won during the trial. The order of trials, as well as the probability of reward attainment and the reward magnitudes per click are not randomized between participants, but pre-assigned for each trial. This is done to rule out random feedback differences between participants.

Fig 2. Schematic illustration of one trial of the modified EEfRT.

A fixation cross (1s, A) is followed by a screen showing probability of reward attainment and reward magnitude per click for 3s (B). Then, after presentation of a ready – screen (1s, C), the main screen for the trial showing a red bar that grows with each click is presented alongside a scale, indicating the current monetary gain and a countdown (20s, D). Finally, task completion is signaled (1.5s, E) and a feedback screen shows the amount of money won (2s, F).

2.1.7 Motoric Abilities

Participants with greater motoric ability exert more clicks throughout the modified version of the EEfRT [24] and studies calibrating an individual number of clicks to succeed within the original EEfRT suggest that participants with higher motoric abilities might also

choose the hard task more often in the original version [14, 32], which does not reflect their actual approach motivation. Therefore, we included 10 motoric trials to test participants' motoric abilities before each version of the EEfRT. Within these motoric trials, participants were instructed to press the spacebar as often as possible within 20 seconds. Critically, participants were not able to gain any rewards in these trials and visual feedback was reduced to a countdown and a display of the number of clicks they exerted. Participants' individual motoric abilities were operationalized as maximal clicks in motoric trials (MaxMot) and included in our statistical models.

2.1.8 Data Analysis

Aggregated data were analyzed using the SPSS 26.0 software* (Chicago, IL, USA). First, we examined the split-half reliability of different task parameters. To this end, we split the dataset into the first and second temporal half of trials for each individual in the original task. In the modified task, split-half reliability was estimated across the two blocks. Furthermore, we calculated internal consistencies of the questionnaire measures used.

To test for the effects of basic predictors on the percentage of hard-task-choices (original EEfRT) and on the mean number of clicks (modified EEfRT), we used GEEs. GEEs are marginal models that allow for robust parameter estimation despite correlated residuals, e.g., due to the clustering of trials within participants [53,54]. Crucially, GEEs are consistent and provide appropriate robust standard errors even when the correlation matrix for the residuals is specified incorrectly [54]. Models were fit using an exchangeable working correlation matrix. Given that our dependent variable (hard-task-choices) was binary, we implemented models using the binomial distribution with a logit link. For the modified task (dependent variable: number of clicks), a gaussian distribution was assumed. All GEE models included the factors trial number, probability (categorical), reward magnitude, and the interaction of probability x reward magnitude (often referred to as "expected value"). Moreover, participants' individual motoric abilities were included in all GEE models.

Pearson correlations were computed between self-reported personality traits (BIS/BAS/TEPS/UPPS), the number of pumps within the BART, the percentage of hard-task-choices within the original EEfRT, and the mean number of clicks within the modified EEfRT, separately for each probability of reward attainment category (low/medium/high), for each reward magnitude category (low/medium/high), as well as for difference scores between these categories. Within the original EEfRT, we formed these categories in line with previous studies [46]; low reward magnitude: <2,30 Euro, medium reward magnitude: 2,31-3,29 Euro, high reward magnitude: >3,30 Euro. For the modified EEfRT, we calculated analogue categories: Low reward magnitude: 1 or 2 Cent per click, medium reward magnitude: 3 Cent per click, high reward magnitude: 4 or 5 Cent per click. Further Pearson correlations were calculated between both versions of the EEfRT for each probability of reward attainment category, each reward magnitude category, as well as between both versions of the EEfRT and follow-up questions regarding individual strategies and motivation. Datasets and syntax can be found at: https://osf.io/35k2w/?view_only=1f315859028c471787a96e153f5ac43a.

2.2 Study 2

2.2.1 Participants

We recruited physically and psychologically healthy, right-handed participants (68.2% female) aged 18 – 50 (*M* = 25.74; *SD* = 5.37) using online notice boards and flyers at various universities. Out of 409 recruited participants, 15 had to be excluded for different reasons (11 participants consumed illegal drugs or hormones within the last 12 months, 1 subject already knew the EEfRT task, 1 subject was ambidextrous,1 subject had missing values for the UPPS, and for 1 subject it was not possible to collect a blood sample). Thus, the final sample consisted of 394 participants. In accordance with Gignac & Szodorai [42], we applied a more conservative criterium (criterion) than in Study 1 considering the discrepancy between published correlations and those correlations we found in our lab and expected only medium-sized

correlations. As intended, statistical power was therefore >.80 (exact 1-β = .98) to detect correlations of ρ = .20 (α = .05). Participants received monetary compensation (10€ per hour) and were told that they could gain additional money based on their collected rewards from the original EEfRT (5% of the virtually collected money). At the end of the study, all participants received an additional 5€ to ensure equity. This amount was always higher than the 5% of the virtually collected money. [new para] The authors assert that all procedures contributing to this work comply with the ethical standards of the relevant national and institutional committees on human experimentation and with the Helsinki Declaration of 1975, as revised in 2008. The study has been approved by the local Ethics Committee of the Medical Chamber of Hamburg. Exclusion criteria comprised the regular intake of any kind of prescribed medication, consumption of illegal drugs over the last 12 months, neurological or medical conditions, and the presence of any mental disorders - in particular affective-, somatoform-, psychotic-, anxiety-, eating-, and adaptive disorders, as well as substance use disorders, high consumption of alcohol (more than 15 glasses per week), nicotine (more than 15 cigarettes per week) or caffeine (more than 4 cups per day), and pregnancy (tested onside).

2.2.2 Procedure

As the original EEfRT and relevant questionnaires (BIS/BAS, UPPS) were deployed as part of a larger study, we will focus only on the most relevant information. A complete description of the procedure will be provided elsewhere. The order of tasks and questionnaires was not randomized and in contrast to Study 1, no motoric trials were included. Participants who fit the inclusion criteria were invited into the lab once. After arriving at the lab and giving their informed consent in written form, participants completed a large series of questionnaires, including the German BIS/BAS [6, 55] and the UPPS [56, 57], as well as a series of computer tasks, including the original EEfRT [21]. The overall experiment lasted for

about 4.5 hours. The EEfRT took place approximately 4 hours after the start of the experiment.

2.2.3 Original EEfRT

The procedure of the original EEfRT was identical to experiment 1 (see 2.1.4 and Fig 1).

2.2.4 Data Analysis

Statistical analysis for the original EEfRT was identical to experiment 1 (see 2.1.8), with the following exceptions: As no motoric trials were included, individual motoric abilities were removed as a factor from the GEE model. In accordance with Study 1, Pearson correlations were computed between self-reported personality traits (BIS/BAS/ UPPS) and the percentage of hard-task-choices within the original EEfRT for each probability of reward attainment (low/medium/high) and each category of reward magnitude (low/medium/high), as well as difference scores between these categories. Datasets and syntax can be found at: https://osf.io/35k2w/?view_only=1f315859028c471787a96e153f5ac43a.

3. Results

3.1 Reliability

We examined the internal consistency of all questionnaires in both studies by estimating Cronbach‘s Alpha. Most questionnaires showed moderate to good internal consistency (see Table 2). We then calculated the reliability of the EEfRT by estimating split-half correlations and applying Spearman-Brown corrections to the resulting estimates. Note however, as we introduced motoric trials within Study 1, we compared reliabilities unadjusted and adjusted for motoric abilities, respectively (see Table 2). Therefore, we calculated the reliability of the percentage of hard-task-choices and clicks after residualizing them on motoric abilities (operationalized as maximal clicks in motoric trials; MaxMot). The overall HTCs and clicks showed high reliability in both studies, within the original EEfRT in Study 1

(Rel =.90; Adj. Rel = .91) and in Study 2 (Rel. = .87), and within the modified EEfRT in Study 1 (Rel = .96; Adj Rel. = .92)

When separately analyzing the data for each probability and reward magnitude category, split-half reliabilities ranged between Rel = .73 and .97 over both studies and task versions. However, when calculating the split-half reliability for the difference scores, some indices showed relatively poor reliability (range: Rel. = .35 to .86), especially the difference between high and medium reward magnitudes. Overall, both versions of the EEfRT showed good reliability and the adjustment for motoric abilities in Study 1 impacted the reliability of the EEfRT only slightly.

Table 2. Internal consistencies and reliabilities for questionnaires and both versions of the EEfRT in Study 1 and 2.

	Study 1	Study 2
Questionnaires		
BAS	α = 0.79	α = 0.74
BIS	α = 0.78	α = 0.79
TEPS Anticipatory	α = 0.67	-
TEPS Consummatory	α = 0.70	-
UPPS Urgency	α = 0.86	α = 0.84
UPPS Premeditation	α = 0.78	α = 0.77
UPPS Perseverance	α = 0.83	α = 0.82
UPPS Sensation Seeking	α = 0.84	α = 0.83
Original EEfRT task		
HTCs	Rel =.90 / Rel_{adj} =.91	Rel =.87
Low Probability HTCs	Rel =.84 / Rel_{adj} =.84	Rel =.81
Medium Probability HTCs	Rel =.79 / Rel_{adj} =.78	Rel =.80
High Probability HTCs	Rel =.77 / Rel_{adj} =.76	Rel =.73
High – Low Probability HTCs	Rel =.76 / Rel_{adj} =.76	Rel =.75
High – Medium Probability HTCs	Rel =.67 / Rel_{adj} =.66	Rel =.57
Low Reward HTCs	Rel =.82 / Rel_{adj} =.83	Rel =.80
Medium Reward HTCs	Rel =.82 / Rel_{adj} =.81	Rel =.78
High Reward HTCs	Rel =.82 / Rel_{adj} =.82	Rel =.79
High – Low Reward HTCs	Rel =.67 / Rel_{adj} =.67	Rel =.66
High – Medium Reward HTCs	Rel =.39 / Rel_{adj} =.38	Rel =.40
Modified EEfRT task		
Clicks	Rel =.96 / Rel_{adj} =.92	-
Low Probability Clicks	Rel =.91 / Rel_{adj} =.87	-
Medium Probability Clicks	Rel =.96 / Rel_{adj} =.91	-
High Probability Clicks	Rel =.97 / Rel_{adj} =.93	-
High – Low Probability Clicks	Rel =.86 / Rel_{adj} =.86	
High – Medium Probability Clicks	Rel =.68 / Rel_{adj} =.68	
Low Reward Clicks	Rel =.93 / Rel_{adj} =.89	-
Medium Reward Clicks	Rel =.95 / Rel_{adj} =.89	-
High Reward Clicks	Rel =.95 / Rel_{adj} =.89	-
High – Low Reward Clicks	Rel =.80 / Rel_{adj} =.80	-
High – Medium Reward Clicks	Rel =.37 / Rel_{adj} =.35	-

Note. Depicted are internal consistencies of questionnaire measures and split-half reliabilities of task measures. α = Cronbach‘s Alpha, Rel = split-half reliability calculated by Spearman-Brown correcting the split-half correlation of task measures (derived by splitting the dataset into the first and second temporal half of trials for each individual in the original task; calculated across the two blocks in the modified task), adj = adjusted for motor abilities: variables were predicted by the maximum number of clicks in motoric trials and residuals were used to calculate reliability, EEfRT = Effort Expenditure for Reward Task, HTC = Hard Task Choices.

3.2 Experiment 1

3.2.1 Original EEfRT - Validity of Basic Task Variables

Participants on average chose the hard task in 53.41% of all trials (*SD* = 18.50%; Range = 2.90 – 100%). We conducted a GEE model to test validity of the basic task variables within the original EEfRT in Study 1 (see Table 3). The GEE model examined the main effects of task-dependent variables (reward magnitude, probability of reward attainment, and trial number) as well as one variable unique to our study (MaxMot) on the percentage of hard-task-choices. In line with previous studies, a significant positive main effect was found for reward magnitude and probability of reward attainment and a significant negative main effect was found for trial number, indicating that all three factors were predictors of percentage of hard-task choices (all *p*s < .001). Furthermore, the interaction of reward and probability (often referred to as “expected value”) did also reach significance, indicating that higher probability did predict a higher percentage of hard-task-choices with increasing reward magnitude (see Fig 3). The factor MaxMot did not reach significance (B = 0.00, $\chi^2(1) = 0.08$, $p = .772$), indicating that motoric ability as measured within the motoric trials did not strongly affect hard task choices within the original EEfRT.

Fig 3. Percentage of hard-task-choices (HTC) within the original EEfRT in Study 1.

Comparison of trials with low probability of reward attainment (left; A), medium probability of reward attainment (middle; B) and high probability of reward attainment (right, C) within the original EEfRT. For each probability category all three categories of reward magnitude (low / medium / high) are displayed. Data points are added as dots for individual scores. Error bars depict a 95% confidence interval (CI) of the mean.

Table 3. GEE Models for basic predictors of percentage of hard-task choices within the original EEfRT in Study 1 and 2 and of mean number of clicks within the modified EEfRT in Study 1.

**Effect**	***B***	***se***	χ^2	***p***
**Study 1 – Original EEfRT**				
Reward Magnitude	0.80	0.08	111.38	**<.001**
Probability 88%[a]	3.44	0.28	146.59	**<.001**
Probability 50%[a]	1.59	0.13	157.97	**<.001**
Probability 88%[a] × Reward Magnitude	1.32	0.20	44.46	**<.001**
Probability 50%[a] × Reward Magnitude	0.51	0.10	27.68	**<.001**
Trial	-0.77	0.12	41.81	**<.001**
MaxMot	0.00	0.01	0.08	.772
**Study 1 – Modified EEfRT**				
Reward Magnitude	4.91	0.46	111.80	**<.001**
Probability 88%[a]	17.80	1.53	135.98	**<.001**
Probability 50%[a]	12.47	1.07	135.35	**<.001**
Probability 88%[a] × Reward Magnitude	-1.83	0.43	18.51	**<.001**
Probability 50%[a] × Reward Magnitude	-1.33	0.38	12.19	**<.001**
Trial	-5.96	0.90	43.58	**<.001**
Block	-2.22	0.57	14.95	**<.001**
MaxMot	0.61	0.05	149.48	**<.001**
**Study 2 – Original EEfRT**				
Reward Magnitude	0.66	0.04	299.55	**<.001**
Probability 88%[a]	3.10	0.14	508.21	**<.001**
Probability 50%[a]	1.60	0.07	587.79	**<.001**
Probability 88%[a] × Reward Magnitude	0.91	0.10	90.63	**<.001**
Probability 50%[a] × Reward Magnitude	0.40	0.05	65.19	**<.001**
Trial	-0.72	0.07	103.37	**<.001**

Note. All models included probability (categorical), reward magnitude, and trial number (divided by the individual maximum number of trials) as within-subjects variables; χ^2 = Wald chi-square; B = regression coefficient; significant effects in **bold.** [a]Reference category: 12% probability

3.2.2 Modified EEfRT - Validity of Basic Task Variables

Participants on average exerted 117.71 number of clicks in each trial (*SD* = 16.03; Range = 77.17 – 156.43). In accordance with our analysis of the original EEfRT (see 3.1.1), we computed a GEE model to test validity of the basic task variables within the modified EEfRT in Study 1 (see Table 3). The GEE Model examined main effects of task-dependent variables (reward magnitude, probability of reward attainment, and trial number) as well as one variable unique to our study (MaxMot) capturing the mean number of clicks. In line with previous studies, significant positive main effects were found for reward magnitude and probability of reward attainment and a significant negative main effect was found for trial number, indicating all three factors were predictors of the mean number of clicks (all *p*s < .001). Furthermore, the interaction of reward and probability also reached significance ($p < .001$), indicating that higher probability predicted a stronger increase of mean number of clicks for smaller reward magnitudes (see Fig 4). The factor MaxMot also reached significance (B = 0.61, $\chi^2(1) = 149.48$, $p < .001$), indicating that participants with greater motoric ability as measured within the motoric trials exerted more clicks within the modified EEfRT.

Fig 4. Mean number of clicks within the modified EEfRT in Study 1.
Comparison of trials with low probability of reward attainment (left; **A**), medium probability of reward attainment (middle; **B**) and high probability of reward attainment (right; **C)** within the modified EEfRT. For each probability category all 5 different reward magnitudes (ranging from 1 Cent (most left) to 5 Cent (most right) are displayed. Data points are added as dots for individual scores. Error bars depict a 95% confidence interval (CI) of the mean.

3.3 Experiment 2

3.3.1 Original EEfRT - Validity of Basic Task Variables

Participants on average chose the hard task in 53.31% of all trials (*SD* = 19.21%; Range = 0 – 100%). We computed a GEE model to test the validity of basic task variables within the original EEfRT in Study 2 (see Table 3). The GEE Model examined main effects of task-dependent variables (reward magnitude, probability of reward attainment, and trial number) on the percentage of hard-task-choices. In line with previous studies and Study 1, significant positive main effects were found for reward magnitude and probability of reward attainment, and a significant negative main effect was found for trial number, indicating that all factors were predictors of the percentage of hard-task choices (all ps < .001). Furthermore, the interaction of reward and probability (often referred to as “expected value”) reached significance ($p < .001$), indicating that higher probability predicted a higher percentage of hard-task-choices with increasing reward magnitude (see Fig 5).

Fig 5. Percentage of hard task choices (HTC) within the original EEfRT in Study 2.

Comparison of trials with low probability of reward attainment (left; A), medium probability of reward attainment (middle; B) and high probability of reward attainment (right; C) within the original EEfRT. For each probability category, all three reward magnitude categories (low / medium / high) are displayed. Data points are added as dots for individual scores. Error bars depict a 95% confidence interval (CI) of the mean

3.4 Personality correlations

To further validate the original and the modified EEfRT, we exploratorily correlated the percentage of hard-task-choices within the original EEfRT (Study 1 and Study 2) and the mean number of clicks within the modified EEfRT (Study 1) with personality traits as well as

the mean number of pumps in the BART (Study 1). We compared all trial categories as well as difference scores (see Table 4 and 5). Difference scores reflect individual differences in the degree to which participants' hard task choices or clicks are influenced by the probability of reward attainment and the reward magnitude, respectively. For instance, positive correlations between traits and difference scores would indicate that a higher trait value is associated with a stronger impact of probability of reward attainment or reward magnitude on task performance. Surprisingly, trait BAS did not correlate significantly with the percentage of hard-task-choices in trials with low probability of reward attainment within the original EEfRT in both studies, nor with the mean number of clicks within the modified EEfRT in trials with low probability of reward attainment (see Fig 6). However, in the original EEfRT in Study 1, trait BAS correlated negatively with the difference score between trials with high probability of reward attainment and low probability of reward attainment. Conversely, trait BIS correlated positively with both difference scores (high probability minus low / medium probability trials). Trait anticipatory pleasure correlated positively with number of hard task choices within trials with low probability of reward attainment, as well as negatively with the difference score between trials with high probability of reward attainment and trials with low probability of reward attainment. Out of these correlations, only one was similar when comparing trials with different reward magnitudes. Trait anticipatory pleasure correlated positively with number of hard-task-choices within trials with low reward magnitude. Trait BIS correlated negatively with the difference score between trials with high reward magnitude and low reward magnitude Importantly, none of these correlations could be replicated in Study 2.

Note, however, that we did not administer the TEPS questionnaire and the BART in Study 2. The analyses for the modified EEfRT, which was administrated in Study 1 only, revealed a different pattern of results compared to the original EEfRT. Trait anticipatory pleasure correlated negatively with the difference score between trials with high probability of

reward attainment and trials with medium probability of reward attainment. Moreover, risk-taking behavior as measured via the BART correlated positively with the mean number of clicks in trials with medium and high probabilities of reward attainment. This finding was similar when examining reward magnitudes. Risk-taking behavior (BART) correlated positively with the mean number of clicks in trials with medium and high reward magnitudes. Across studies, tasks, and task parameters, the UPPS scales were largely unrelated to task performance.

Fig 6. Pearson correlations between trait BAS scores (z-standardized) and task performance (Study 1 and 2).

Correlations for the mean number of clicks within trials with low probability of reward attainment in the modified EEfRT (**A:** Study 1) and percentage of hard task choices within trials with low probability of reward attainment in the original EEfRT (**B**: Study 1; **C:** Study 2). All depicted correlations were nonsignificant with $p > .05$.

Table 4. Correlations between the original EEfRT (percentage of hard-task -choices), the modified EEfRT (mean number of clicks) and trait variables (Study 1 and 2).

Trait variable	Reward Magnitude				
**Original EEfRT (Study 1)**	**low**	**medium**	**high**	**high-low**	**high-medium**
BAS	.113	.055	-.055	-.159	-.147
BIS	.066	-.066	-.135	**-.180***	-.086
TEPS – anticipatory pleasure	**.235****	.178	.160	-.097	-.034
TEPS – consummatory pleasure	.061	.083	.023	-.041	-.083
UPPS-Urgency	-.131	-.072	-.021	.112	.070
UPPS-Premeditation	-.067	-.003	.069	.125	.094
UPPS-Perseverance	-.039	-.042	.090	.115	.175
UPPS-Sensation Seeking	.117	.107	.074	-.053	-.049
BART	.050	.115	.117	.050	-.004
**Original EEfRT (Study 2)**					
BAS	-.026	.006	.020	.046	.016
BIS	-.029	-.035	-.051	-.013	-.015
UPPS-Urgency	.009	-.009	-.025	-.032	-.019
UPPS-Premeditation	.017	.016	-.010	-.027	-.035
UPPS-Perseverance	-.007	.026	-.010	-.001	-.049
UPPS-Sensation Seeking	.036	.079	.092	.043	.005
**Modified EEfRT (Study 1)**					
BAS	.046	.006	-.006	-.089	-.044
BIS	.101	.082	.069	-.060	-.037
TEPS – anticipatory pleasure	.080	-.016	-.020	-.168	-.014
TEPS – consummatory pleasure	.069	.042	.062	-.018	.072
UPPS-Urgency	-.017	-.031	-.054	-.059	-.082
UPPS-Premeditation	-.060	-.051	-.041	.037	.032
UPPS-Perseverance	-.150	-.095	-.060	.159	.115
UPPS-Sensation Seeking	.024	.035	.055	.048	.071
BART	.154	**.224***	**.253****	.145	.120

Note. EEfRT = Effort Expenditure for Rewards Task; BAS = Behavioral Activation System Scale; BIS = Behavioral Inhibition System Scale; TEPS: Temporal Experience of Pleasure Scale; UPPS = Urgency, Premeditation, Perseverance, and Sensation Seeking Impulsive Behavior Scale; BART = Balloon Analogue Risk Task. Correlations with (unadjusted) p <.05 are printed in bold. The significance level adjusted for multiple comparisons (k = 5 hypothesis tests for each correlate) was set to $p < .01$. * $p < .05$; ** $p <.01$

Table 5. Correlations between the original EEfRT (percentage of hard-task -choices), the modified EEfRT (mean number of clicks) and trait variables (Study 1 and 2)

**Trait variable**	**Reward Probability**				
**Original EEfRT (Study 1)**	**12%**	**50%**	**88%**	**88-12%**	**88-50%**
BAS	.148	.018	-.073	**-.181***	-.074
BIS	-.114	-.105	.165	**.203***	**.238****
TEPS – anticipatory pleasure	**.291****	.164	.047	**-.243****	-.143
TEPS – consummatory pleasure	.154	-.007	-.010	-.149	.000
UPPS-Urgency	-.095	-.066	-.065	.050	.023
UPPS-Premeditation	.058	.016	-.131	-.131	-.116
UPPS-Perseverance	.029	-.039	-.006	-.031	.037
UPPS-Sensation Seeking	.115	.086	.095	-.051	-.023
BART	.021	.123	.148	.068	-.023
**Original EEfRT (Study 2)**					
BAS	-.024	-.022	.059	.060	.079
BIS	-.066	-.027	.004	.063	.036
UPPS-Urgency	-.035	-.015	.050	.064	.061
UPPS-Premeditation	.006	.023	-.017	-.017	-.043
UPPS-Perseverance	-.018	.051	-.026	.000	-.084
UPPS-Sensation Seeking	.025	.065	**.105***	.044	.015
**Modified EEfRT (Study 1)**					
BAS	.015	.045	-.007	-.024	-.123
BIS	.064	.108	.075	.001	-.071
TEPS – anticipatory pleasure	.075	.037	-.055	-.145	**-.222***
TEPS – consummatory pleasure	.049	.080	.048	-.010	-.072
UPPS-Urgency	.016	-.040	-.081	-.101	-.103
UPPS-Premeditation	-.032	-.036	-.080	-.044	-.111
UPPS-Perseverance	-.064	-.123	-.119	-.046	.000
UPPS-Sensation Seeking	.017	.041	.055	.036	.037
BART	.126	**.230***	**.249****	.106	.067

Note. EEfRT = Effort Expenditure for Rewards Task; BAS = Behavioral Activation System Scale; BIS = Behavioral Inhibition System Scale; TEPS: Temporal Experience of Pleasure Scale; UPPS = Urgency, Premeditation, Perseverance, and Sensation Seeking Impulsive Behavior Scale; BART = Balloon Analogue Risk Task. Correlations with (unadjusted) p <.05 are printed in bold. The significance level adjusted for multiple comparisons (k = 5 hypothesis tests for each correlate) was set to p < .01. * p < .05; ** p <.01

3.5 Secondary Analyses

As Study 1 is one of the first studies to test two versions of the EEfRT within one experimental design, we further evaluated the validity of both tasks by exploratorily correlating the main dependent variables of both task versions within all three different probability of reward attainment categories (low: 12% / medium: 50% / high: 88%; see Fig 7) and all three different reward magnitude categories (low / medium / high). The correlations between the matching dependent variables of both tasks were significant for all probabilities of reward attainment (*r* ranging from .192 - .305). Furthermore, the correlation between the matching dependent variables of both tasks was significant for trials with medium (r = .277) and high reward magnitudes (r = .218). Note that some discriminant correlations of non-matching reward probabilities or reward magnitudes exceeded the respective convergent correlations. The results indicate an overall linkage between performance on both task versions (albeit only small to moderate in size considering that the same construct should be measured). Subjects who choose the hard task more often on the original EEfRT also exert more clicks within the modified EEfRT (see Table 6).

Additionally, we asked participants to self-evaluate aspects that might have influenced their effort allocation individually for both task versions and asked them about their motivation to win money throughout the whole study. We then exploratorily correlated theses evaluations to their actual effort allocation in Study 1 comparing different trial categories and difference scores (see Table 7 and 8). In line with our GEE analysis, which indicated probability of reward attainment to be strongly connected to actual task performance, participants` self-evaluated importance of this factor for their task performance correlated moderately to strongly with various trial categories in both task versions of the EEfRT (see Table 7 and 8). The self-evaluated importance of reward magnitude was less strongly associated with performance in both task versions, although some moderately sized correlations emerged. When correlating participants` self-evaluated importance of fatigue for

their task performance throughout the task, only one significant effect was observed. The number of clicks within the modified EEfRT correlated positively with the difference score between trials with high probability of reward attainment and medium probability of reward attainment. When correlating participants` self-evaluated importance of resting their fingers for their performance throughout the modified EEfRT, this evaluation also correlated significantly with the difference score between trials with high probability of reward attainment and trials with medium probability of reward attainment. A comparable result pattern was found for the original EEfRT. The strategy to rest their fingers was especially negatively related to hard-task-choices in trials with low reward probabilities and medium reward magnitudes. Finally, when correlating participants` motivation to win money throughout the whole study, the mean number of clicks within the modified EEfRT correlated significantly within trials with all probability levels, as well as with trials with medium and high reward magnitudes, indicating that participants with high motivation to win money performed more clicks in almost all trial categories (see Table 7 and 8). In contrast to the modified EEfRT, the percentage of hard-task-choices within the original EEfRT did not correlate with participants` motivation to win money in any trial category.

Fig 7. Pearson correlations between task performance in both tasks in Study 1.

Correlations between the percentage of hard-task-choices (HTC %) within the original EEfRT and the mean number of clicks within the modified EEfRT for **A:** trials with low probability of reward attainment (=12%), **B:** medium probability of reward attainment (=50%) and **C:** high probability of reward attainment (=88%). All depicted correlations were significant with p <.05

Table 6. Zero-order correlations between the original EEfRT (percentage of hard-task - choices) and the modified EEfRT (mean number of clicks) for different probabilities of reward attainment and different reward magnitudes.

	**Reward Probability**				
**Task**	**Modified EEfRT**				
**Original EEfRT**	12%	50%	88%	88-12%	88-50%
12%	**.216***	.085	-.046	**-.302****	**-.313****
50%	**.340****	**.305****	**.221***	-.176	**-.180***
88%	.093	.140	**.192***	.086	.138
88-12%	-.146	.004	.156	**.331****	**.372****
88-50%	**-.300****	**-.227***	-.097	**.256****	**.299****
	**Reward Magnitude**				
**Task**	**Modified EEfRT**				
**Original EEfRT**	low	medium	high	high-low	high-medium
low	.160	.043	.020	**-.242****	-.077
medium	**.324****	**.277****	**.276****	-.108	.022
high	**.215***	**.210***	**.218***	-.015	.047
high-low	.024	.137	.166	**.228***	.116
high-medium	-.162	-.104	-.092	.129	.032

Notes. EEfRT = Effort Expenditure for Rewards Task. Correlations with (unadjusted) p <.05 are printed in bold. The adjusted significance level was set to $p < .01$. * $p < .05$; ** $p <.01$

Table 7. Zero-order correlations between the original EEfRT (percentage of hard-task - choices), the modified EEfRT (mean number of clicks) and follow-up questions concerning task strategy and motivation in Study 1 for different probabilities of reward attainment.

**Strategy and motivation**	**Reward Probability**				
**Original EEfRT**	**12%**	**50%**	**88%**	**88-12%**	**88-50%**
Reward	.118	**.219***	.041	-.086	**-.207***
Probability	**-.513****	**-.232***	.128	**.552****	**.348****
Fatigue	-.092	-.082	-.078	.040	.030
Resting fingers	**-.199***	-.156	-.093	.130	.100
Motivation to win money	-.085	-.002	.069	.120	.054
**Modified EEfRT**					
Reward	.140	.172	.155	-.007	-.026
Probability	**-.200***	.017	.144	**.382****	**.312****
Fatigue	-.122	-.055	.030	.175	**.204***
Resting fingers	-.171	-.140	-.053	.149	**.201***
Motivation to win money	**.192***	**.231***	**.238****	.015	.035

Notes. EEfRT = Effort Expenditure for Rewards Task. The following five-point Likert-scales ranging from "not at all" (1) to "a lot" (5) were administered separately for each task: Reward: "How much did the reward magnitudes influence you on the task?"; Probability: "How much did the probabilities of reward attainment influence you on the task?"; Fatigue: "How much did Fatigue influence you on the task?"; Resting Fingers: "How much did attempts to rest your fingers influence you on the task?". The last question (same response format) was asked only once (i.e., not separately for each task): Money: "How motivated were you to win money throughout the whole study?". Correlations with (unadjusted) p <.05 are printed in bold. The significance level adjusted for multiple comparisons (k = 5 hypothesis tests for each correlate) was set to $p < .01$. * $p < .05$; ** $p < .01$

Table 8. Zero-order correlations between the original EEfRT (percentage of hard-task - choices), the modified EEfRT (mean number of clicks) and follow-up questions concerning task strategy and motivation in Study 1 for different reward magnitudes.

**Strategy and motivation**	**Reward Magnitude**				
**Original EEfRT**	**low**	**medium**	**high**	**high-low**	**high-medium**
Reward	.029	.153	**.284****	**.212***	.162
Probability	**-.204***	**-.243****	**-.389****	-.129	-.177
Fatigue	-.065	-.115	-.086	-.009	.045
Resting fingers	-.108	**-.231***	-.152	-.022	.117
Motivation to win money	-.002	-.024	-.052	-.043	-.035
**Modified EEfRT**					
Reward	.116	**.190***	**.196***	.118	.039
Probability	-.123	.020	.059	**.306****	.139
Fatigue	-.100	-.022	-.023	.134	-.006
Resting fingers	-.163	-.124	-.096	.123	.087
Motivation to win money	.177	**.253****	**.271****	.136	.088

Notes. EEfRT = Effort Expenditure for Rewards Task. The following five-point Likert-scales ranging from "not at all" (1) to "a lot" (5) were administered separately for each task: Reward: "How much did the reward magnitudes influence you on the task?"; Probability: "How much did the probabilities of reward attainment influence you on the task?"; Fatigue: "How much did Fatigue influence you on the task?"; Resting Fingers: "How much did attempts to rest your fingers influence you on the task?". The last question (same response format) was asked only once (i.e., not separately for each task): Money: "How motivated were you to win money throughout the whole study?". Correlations with (unadjusted) p <.05 are printed in bold. The significance level adjusted for multiple comparisons (k = 5 hypothesis tests for each correlate) was set to $p < .01$. * $p < .05$; ** $p <.01$

4.Discussion

In the present study, we aimed to (1) validate the original EEfRT [21] and a modified version of the EEfRT [24] as measures of approach motivation by directly comparing both versions within one experimental design (Study 1) and to replicate the reliability and validity of the original EEfRT within a large sample (Study 2). We further aimed to (2) test the correlations between self-reported personality traits and behavioral measurements for different trial categories and difference scores, as well as between self-reported strategy usage and motivation and task performance in an exploratory fashion. We will now discuss the implication of the current findings.

4.1 Reliability and Validity of the original and modified version of the EEfRT

Supporting the results of previous studies [14, 22, 24], both the original EEfRT and the modified EEfRT showed overall good split-half reliability, indicating that both versions are producing reliable results. In terms of validity, our results are mixed. The basic validity of both tasks as measures of reward-dependent approach motivation received further support from the GEE models in both studies as the basic task variables are in line with previous studies. In particular, we replicated the typical pattern of effects of reward magnitude, probability of reward attainment, and trial number on the mean number of clicks (modified EEfRT) and the percentage of hard-task-choices (original EEfRT). Furthermore, we found that the two versions of the EEfRT intercorrelated significantly within all three matching probability of reward attainment categories as well as in trials with medium and high reward magnitudes in Study 1. However, these correlations have to be considered small to moderate given that the same construct is supposed to be measured. Thus, differences between the two tasks need to be considered and will be discussed below.

Regarding the relationship between self-reported personality traits and behavioral task measures, our correlations in both studies showed only very weak support for such a link. Only in Study 1, trait BAS and trait anticipatory pleasure correlated significantly with the

percentage of hard task choices within the original EEfRT and with the mean number of clicks within the modified EEfRT, for some task parameters. However, in contrast to our expectations, the correlating task parameters were not fully consistent with previous studies [23, 24]. Furthermore, trait BIS correlated with task performance in the original EEfRT in Study 1, indicating that trait BIS moderated the dependence of hard-task choices on reward magnitude (negatively related to BIS) and probability (positively related to BIS). However, as we analyzed the correlations of trait BAS and trait BIS for the original EEfRT within a larger sample in Study 2, none of these correlations replicated. Furthermore, other self-reported traits - especially impulsivity as measured via the UPPS as well as consummatory pleasure as measured via the TEPS - did not show meaningful correlations with the behavioral measures in either study.

Overall, these findings raise further questions about the existence and magnitude of such links, supporting studies not replicating them [22, 25, 26]. In particular, some correlations in previous work as well as Study 1 may have been overestimated due to random sampling error. Significant correlations may be at least partly attributable to the large number of possible correlations between task parameters and self-report measures. Supporting this notion, when correcting for multiple testing, only some effects of trait anticipatory pleasure and BIS remained significant, indicating that some of our findings might be false positives. Analyzing the correlations between performance on the BART and both task versions of the EEfRT in Study 1 revealed unexpected correlations between the mean number of clicks within the modified EEfRT and the mean number of pumps within the BART, indicating that risk-taking behavior might have impacted task performance as well, although this requires further replication.

Taken together, the results of our data show a very mixed pattern regarding the validity of the EEfRT. Therefore, our results support a multiply determined view on behavioral measurements [42]. In line with this, previous studies indicate that effort allocation

within the EEfRT can be manipulated by a wide range of factors, ranging from mood inductions [23] over neurophysiological manipulations [24, 28, 45] to the influence of reduced motivation [21], or the intake of caffeine [45]. So how does a person decide whether to increase effort to potentially gain a greater monetary reward within the EEfRT? Our mixed pattern of results shows that there is no simple answer to this question. Especially the impact of reward attributes hints at a complex pattern behind participants' decisions and at the importance of individual reward evaluation.

Reward-based decision making is not a uniform process, it can rather be described as a set of distinct cognitive processes, which together direct the evaluation of a reward and thus form a person`s decisions within a concrete situation. According to Orsini et al. [61], reward-based (or "value-based") decision-making is comprised of three phases: 1. Decision representation (different options are identified, as are the costs and benefits associated with each option) and option valuation (each option is also assessed in terms of its subjective value in the moment of the decision), 2. action selection and 3. outcome evaluation (the value of the outcome of a choice is compared with the expected value of that outcome). It is reasonable to assume that the evaluation of potential benefits and costs can differ greatly between participants. Importantly, these individual differences may be insufficiently captured by typical personality trait questionnaires. A potentially important factor is the type of reward and how much a person values this reward. Real-life reward types include e.g., social [62], physical [63, 64], and recreational [65, 66] rewards and their valuation has been successfully differentiated via self-reports [67]. As stated above, Lopez-Gamundi & Wardle [51] were able to show that participants chose the hard task more often within a modified version of the EEfRT using cognitive tasks (C-EEfRT), although participants described the modified version as more difficult. The cognitive challenge of the modified version might have been rewarding in itself (although the monetary reward magnitude was unchanged). These results indicate that "costs" and "benefits" within a task can also be related to properties of the task itself.

To reach a better understanding of the self-evaluated aspects which might have influenced participants decisions, we asked them a series of questions about their strategies and motivation at the end of Study 1. We were able to show that effort allocation on both task versions was impacted by the self-evaluated importance of probability of reward attainment and reward magnitude, indicating that participants show some awareness of the factors that impact their behavior. Surprisingly, participants' self-evaluated motivation to win money throughout the whole study correlated positively only with the mean number of clicks within the modified EEfRT, in all three categories of probability of reward attainment as well as in trials with medium and high reward magnitudes. The percentage of hard-task-choices within the original EEfRT was not correlated with this self-evaluated monetary motivation. These results indicate that the individual evaluation of "costs" and "benefits" differs between both versions of the EEfRT, and hints at a potentially better validity of the modified EEfRT. Lastly, one should also consider the nature of the questionnaires which assess personality traits, like trait BAS [6,55], trait anticipatory pleasure [7] or trait impulsivity [56, 57]: These questionnaires consist of questions about various different situations, most of them complex real-life situations. Linking those scales to behavior in one artificial experimental situation might be rather difficult. There are several potential reasons for the lack of consistent associations. These pertain to (1) the validity of the questionnaire measures (e.g., self-reported traits only reflect one aspect of personality), (2) the validity of the tasks (e.g., factors other than approach motivation may substantially affect task performance), and (3) the similarity of the measured constructs. Regarding the last point, one issue that deserves particular attention is the breadth of global trait measures as compared to the narrowness of task behavior in one laboratory situation. For instance, the EEfRT could be a valid measure of approach motivation in this circumscribed situation and nevertheless be too narrow for the assessment of broad trait-like behavioral tendencies.

4.2 Limitations and future directions

Although we analyzed two rather large samples to test the reliability and validity of the original and the modified version of the EEfRT and our study is one of the first to directly compare two versions of the EEfRT within one experimental design, there are still some limitations to our study. First, although we tried to stick as close to the original version of the EEfRT as possible [21], there is still a noteworthy adaption, which might have impacted participants` behavior in our "original" EEfRT strongly. The adaption is based on a study by Hughes et al. [46], who decided to pay participants a percentage of the virtually won money instead of paying participants the money which they have won on two random trials [21]. We followed this adaption, as we expected the non-random payment to increase participants` overall approach motivation. However, we did not expect this adaption to change the basic response pattern in any significant way, which is also supported by our results replicating the basic predictors (i.e., reward attributes). Nonetheless, as we stated in the introduction, many adaptions of the EEfRT have been used in various studies, ranging from reduced complexity by fixing the monetary rewards [37], or by removing trials with low probability of reward attainment [38] to the addition of "loose" – trials [52], or the addition of a social component [47]. Thus, we cannot rule out that our modification might have caused a significant change in behavior within the original EEfRT. In this regard, our modification might have impacted participants' strategic behavior, as the random payment introduced by the original EEfRT [21] might reduce strategic task choices compared to our version of the EEfRT. Therefore, another direct comparison of these two task versions is needed in future studies.

Furthermore, although both task versions used in Study 1 correlated significantly, suggesting some overlap, the correlations ranging from r = .160 to .305 between matching variables indicate that the variables captured by the two tasks also differ substantially. Fig 7 indicates that this might partly be a result of floor-, and ceiling-effects regarding the effort allocation within original EEfRT (especially in trials with low and high probability of reward

attainment). Future studies should therefore always consider that any change made to the original EEfRT e.g., to make the task fit to the experimental setting could lead to substantial changes in behavior and should therefore carefully compare any new version of the EEfRT to the original version. Furthermore, the floor- and ceiling-effects could be a general problem of the original EEfRT – at least when testing young healthy participants. These effects should also be considered, for instance, when comparing healthy participants to patients with impaired approach motivation. Second, although we were able to test the original EEfRT within a large sample in Study 2, the comparison of both studies is limited. As stated in the methods section, the original EEfRT was part of a larger genetic study in Study 2 and task administration took place approximately 4 hours after start of the testing session. It is reasonable to assume that completing the task after 4 hours of testing might have influenced participants' fatigue or boredom. Study 1 on the other hand lasted for only one hour, which might have led to less fatigue or boredom.

Third, both samples consisted mainly of young healthy students and both samples were not balanced in terms of age or gender. Thus, it remains an open question whether the results of our research can be generalized to broader populations. As the original EEfRT has been demonstrated to be a sensitive tool for detecting reduced approach motivation within various clinical samples [32–38], we cannot rule out that the results we found regarding the reliability and validity of the EEfRT could show a different pattern for other populations, e.g., within clinical samples. Fourth, as we discussed above, the rather mixed results regarding the validity of the original and the modified EEfRT might be a result of correlating scales from self-reports summarizing a large set of real-life situations and behavioral measurements within one laboratory task. The EEfRT assesses the investment of physical effort for monetary rewards. Other forms of effort as well as other forms of rewards should be introduced and tested. Furthermore, we suggest that assessing approach motivation should not be limited to one specific "cost" and one specific "benefit". For instance, future work might

use a range of tasks or a range of variations of the EEfRT and calculate scales comparable to questionnaires assessing personality traits. The EEfRT offers a solid foundation to probe other forms of “costs” (e.g., the C-EEfRT which uses cognitive costs) [51] and a variety of different “benefits” (e.g., food portions) [50].

4.3 Conclusion

Taken together, our findings provide additional support for the split-half reliability of the original and the modified version of the EEfRT. Furthermore, the correlations between both task versions provide evidence for some overlap of the two tasks. However, these correlations have to be considered small to moderate given that the same construct was targeted. Thus, these findings also hint at noteworthy differences between the two tasks. The results regarding the validity of the tasks are mixed. While the basic predictors of both task versions replicated well in both studies and are also supported by participants’ self-evaluated importance of these factors, we were not able to replicate previous findings linking trait BAS and trait anticipatory pleasure to effort allocation within both versions of the EEfRT as only some performance parameters in Study 1 correlated with self-reported personality traits. Study 2 with a larger sample did not reveal any correlation involving trait BAS for the original EEfRT. Furthermore, self-evaluated motivation hints at a possible advantage of the modified EEfRT regarding its validity. Our results indicate a complex interplay of personality traits, task properties, and reward attributes. Furthermore, they highlight the importance of analyzing the reliability and validity of the EEfRT and any modification applied to the task.

Acknowledgements

This research did not receive any specific grant from funding agencies in the public, commercial, or not-for-profit sectors. There were no conflicts of interest. The studies and the corresponding hypotheses were not preregistered. Datasets and syntax will be made publicly available here: https://osf.io/35k2w/?view_only=1f315859028c471787a96e153f5ac43a.

Furthermore, we would like to thank Karoline Rosenkranz for supporting our project by collecting the data of study 2.

5. References

1. Harmon-Jones E, Harmon-Jones C, Price TF. What is approach motivation? Emot Rev. 2013; 5(3): 291–295. doi: 10.1177/1754073913477509
2. Lazarus RS. Cognition and motivation in emotion. Am Psychol. 1991; 46(4): 352–367. doi: 10.1093/jcr/ucaa025 025
3. Lazarus RS. Progress on a cognitive-motivational-relational theory of emotion. Am Psychol. 1991; 46(8): 819–834. doi: 10.1037/0003-066X.46.8.819
4. Gray JA. Brain Systems that mediate both emotion and cognition. Cogn Emot. 1990; 4(3): 269–288. doi: 10.1080/02699939008410799
5. Corr PJ. Testing problems in JA Gray's personality theory: a commentary on Matthews and Gillard (1999). Pers Individ Dif. 2001; 30(2): 333–352. doi: 10.1016/S0191-8869(00)00028-3
6. Carver CS, White TL. Behavioral inhibition, behavioral activation, and affective responses to impending reward and punishment: The BIS/BAS scales. J Pers Soc Psycholog. 1994; 67(2): 319–319. doi: 10.1037/0022-3514.67.2.319
7. Gard DE, Gard MG, Kring AM, John OP. Anticipatory and consummatory components of the experience of pleasure: A scale development study. J Res Per. 2006; 40(6): 1086–1102. doi: 10.1037/abn0000244
8. Mischel W. Personality and assessment. New York: Wiley; 1968.
9. Mischel W. From personality and assessment (1968) to personality science, 2009. J Res Per. 2009; 43(2): 282–290. doi: 10.1016/j.jrp.2008.12.037

10. Dang J, King KM, & Inzlicht M. Why Are Self-Report and Behavioral Measures Weakly Correlated? Trends Cogn Sci. 2020; 24(4): 267–269. doi: 10.1016/j.tics.2020.01.007
11. Salamone JD, Cousins MS, Bucher S. Anhedonia or anergia? Effects of haloperidol and nucleus accumbens dopamine depletion on instrumental response selection in a T-maze cost/benefit procedure. Behav Brain Res. 1994; 65(2): 221–229. doi: 10.1016/0166-4328(94)90108-2
12. Cléry-Melin M-L, Schmidt L, Lafargue G, Baup N, Fossati P, Pessiglione M. Why don't you try harder? An investigation of effort production in major depression. PLoS One. 2011; 6(8): e23178. doi: 10.1371/journal.pone.0023178
13. Kurniawan IT, Seymour B, Talmi D, Yoshida W, Chater N, Dolan RJ. Choosing to make an effort: the role of striatum in signaling physical effort of a chosen action. J Neurophysiol. 2010; 104(1): 313–321. doi: 10.1152/jn.00027.2010
14. Reddy LF, Horan WP, Barch DM, Buchanan RW, Dunayevich E, Gold JM, et al. Effort-based decision-making paradigms for clinical trials in schizophrenia: Part 1—Psychometric characteristics of 5 paradigms. Schizophr Bull. 2015; 41(5): 1045–1054. doi: 10.1093/schbul/sbv089
15. Hartmann MN, Hager OM, Reimann AV, Chumbley JR, Kirschner M, Seifritz E, et al. Apathy but not diminished expression in schizophrenia is associated with discounting of monetary rewards by physical effort. Schizophr Bull. 2015; 41(2): 503–512. doi: 10.1093/schbul/sbu102
16. Hartmann MN, Hager OM, Tobler PN, Kaiser S. Parabolic discounting of monetary rewards by physical effort. Behav Processes. 2013; 100: 192–196. doi: 10.1016/j.beproc.2013.09.014
17. Klein-Flügge MC, Kennerley SW, Saraiva AC, Penny WD, Bestmann S. Behavioral modeling of human choices reveals dissociable effects of physical effort and temporal

delay on reward devaluation. PLoS Comput Biol. 2015; 11(3): e1004116. doi: 0.1371/journal.pcbi.1004116

18. Hershenberg R, Satterthwaite TD, Daldal A, Katchmar N, Moore TM, Kable JW, et al. Diminished effort on a progressive ratio task in both unipolar and bipolar depression. J Affect Disord. 2016; 196: 97–100. doi: 10.1016/j.jad.2016.02.003
19. Lane SD, Cherek DR, Tcheremissine OV, Steinberg JL, & Sharon JL. Response perseveration and adaptation in heavy marijuana-smoking adolescents. Addict Behav. 2007; 32(5): 977–990. doi: 10.1016/j.addbeh.2006.07.007
20. Strauss GP, Whearty KM, Morra LF, Sullivan SK, Ossenfort KL, & Frost KH. Avolition in schizophrenia is associated with reduced willingness to expend effort for reward on a Progressive Ratio task. Schizophr Res. 2016; 170(1): 198–204. doi: 10.1016/j.schres.2015.12.006
21. Treadway MT, Buckholtz JW, Schwartzman AN, Lambert WE, Zald DH. Worth the 'EEfRT'? The effort expenditure for rewards task as an objective measure of motivation and anhedonia. PLoS One. 2009; 4(8): e6598. doi: 10.1371/journal.pone.0006598
22. Horan WP, Felice Reddy L, Barch DM, Buchanan RW, Dunayevich E, Gold JM, et al. Effort-based decision-making paradigms for clinical trials in schizophrenia: Part 2—External validity and correlates. Schizophr Bull. 2015; 41(5): 1055–1065. doi: 10.1093/schbul/sbv090
23. Geaney JT, Treadway MT, Smillie LD. Trait anticipatory pleasure predicts effort expenditure for reward. PLoS One. 2015; 10(6): e0131357. doi: 10.1371/journal.pone.0131357
24. Ohmann HA, Kuper N, Wacker J. A low dosage of the dopamine D2-receptor antagonist sulpiride affects effort allocation for reward regardless of trait extraversion. Personal Neurosci. 2020; 3: E7. doi: 10.1017/pen.2020.7

25. Anand D, Oehlberg KA, Treadway MT, Nusslock R. Effect of failure/success feedback and the moderating influence of personality on reward motivation. Cogn Emot. 2016; 30(3): 458–471. doi: 10.1080/02699931.2015.1013088

26. Kaack I, Chae J, Shadli SM, Hillman K. Exploring approach motivation: Correlating self-report, frontal asymmetry, and performance in the Effort Expenditure for Rewards Task. Cogn Affect Behav Neurosci. 2020; 20(6): 1234–1247. doi: 10.3758/s13415-020-00829-x

27. Smillie LD. What is reinforcement sensitivity? Neuroscience paradigms for approach-avoidance process theories of personality. Eur J Pers. 2008; 22(5): 359–384. doi: 10.1002/per.674

28. Ohmann HA, Kuper N, Wacker J. Left frontal anodal tDCS increases approach motivation depending on reward attributes. Neuropsychologia. 2018; 119: 417–423. doi: 10.1016/j.neuropsychologia.2018.09.002

29. Harmon-Jones E, Gable PA. On the role of asymmetric frontal cortical activity in approach and withdrawal motivation: An updated review of the evidence. Psychophysiol. 2017; 55(1): e12879. doi: 10.1111/psyp.12879

30. Rutherford HJV, Lindell AK. Thriving and surviving: Approach and avoidance motivation and lateralization. Emot Rev. 2011; 3(3): 333–343. doi: 10.1177/1754073911402392

31. Wardle MC, Treadway MT, Mayo LM, Zald DH, de Wit H. Amping up effort: Effects of d-amphetamine on human effort-based decision-making. J Neurosci. 2011; 31(46): 16597–16602. doi: 10.1523/JNEUROSCI.4387-11.2011

32. Fervaha G, Graff-Guerrero A, Zakzanis KK, Foussias G, Agid O, Remington G. Incentive motivation deficits in schizophrenia reflect effort computation impairments

during cost-benefit decision-making. J Psychiatr Res. 2013; 47(11): 1590–1596. doi: 10.1016/j.jpsychires.2013.08.003

33. Barch DM, Treadway MT, Schoen N. Effort, anhedonia, and function in schizophrenia: Reduced effort allocation predicts amotivation and functional impairment. J Abnorm Psychol. 2014; 123(2): 387–397. doi: 10.1037/a0036299

34. McCarthy JM, Treadway MT, Bennett ME, Blanchard JJ. Inefficient effort allocation and negative symptoms in individuals with schizophrenia. Schizophr Res. 2016; 170(2-3): 278–284. doi: 10.1016/j.schres.2015.12.017

35. Chang WC, Chu AOK, Treadway MT, Strauss GP, Chan SKW, Lee EHM, et al. Effort-based decision-making impairment in patients with clinically-stabilized first-episode psychosis and its relationship with amotivation and psychosocial functioning. Eur Neuropsychopharmacol. 2019; 29(5): 629–642. doi: 10.1016/j.euroneuro.2019.03.006

36. Treadway MT, Bossaller NA, Shelton RC, Zald DH. Effort-based decision-making in major depressive disorder: A translational model of motivational anhedonia. J Abnorm Psychol. 2012; 121(3): 553–558. doi: 10.1037/a0028813

37. Yang XH, Huang J, Zhu CY, Wang YF, Cheung EF, Chan RC, et al. Motivational deficits in effort-based decision making in individuals with subsyndromal depression, first-episode and remitted depression patients. Psychiatry Res. 2014; 220(3): 874–882. doi: 10.1016/j.psychres.2014.08.056

38. Damiano CR, Aloi J, Treadway M, Bodfish JW, Dichter GS. Adults with autism spectrum disorders exhibit decreased sensitivity to reward parameters when making effort-based decisions. J Neurodev Disord. 2012; 4(1): 13. doi: 10.1186/1866-1955-4-13

39. Nguyen NH, Albert AB, Van Orman S, Forken P, Blatt SD, Fremont WP, et al. Effort valuation and psychopathology in children and adults. Psychol Med. 2019; 49(16): 2801–2807. doi: 10.1017/S0033291718003884

40. Hughes DM, Yates MJ, Morton EE, & Smillie LD. Asymmetric frontal cortical activity predicts effort expenditure for reward. Cogn Affect Behav Neurosci. 2015; 10(7): 1015–1019. doi: 10.1093/scan/nsu149

41. Huang J, Yang X-H, Lan Y, Zhu C-Y, Liu X-Q, Wang Y-F, et al. Neural substrates of the impaired effort expenditure decision making in schizophrenia. Neuropsychology. 2016; 30(6): 685–696. doi: 10.1037/neu0000284

42. Gignac GE, Szodorai ET. Effect size guidelines for individual differences researchers. Pers Individ Dif. 2016; 102: 74–78. doi: 10.1016/j.paid.2016.06.069

43. Open Science Collaboration. Estimating the reproducibility of psychological science. Science. 2015; 349(6251): aac4716. doi: 10.1126/science.aac4716

44. Kuper N, Käckenmester W, Wacker J. Resting Frontal EEG Asymmetry and Personality Traits: A Meta-analysis. Eur J Pers. 2019; 33(2): 154–175. doi: 10.1002/per.2197

45. Wardle MC, Treadway MT, De Wit H. Caffeine increases psychomotor performance on the effort expenditure for rewards task. Pharmacol Biochem Behav. 2012; 102(4): 526–531. doi: 10.1016/j.pbb.2012.06.016

46. Hughes DM, Yates MJ, Morton EE, Smillie LD. Asymmetric frontal cortical activity predicts effort expenditure for reward. Cogn Affect Behav Neurosci. 2015; 10(7): 1015–1019. doi: 10.1093/scan/nsu149

47. Gilman JM, Treadway MT, Curran MT, Calderon V, Evins AE. Effect of social influence on effort-allocation for monetary rewards. PLoS One. 2015; 10(5): e0126656. doi: 10.1371/journal.pone.0126656

48. Hughes JR, Budney AJ, Muellers SR, Lee DC, Callas PW, Sigmon SC, et al. Does tobacco abstinence decrease reward sensitivity? A human laboratory test. Nicotine Tob Res. 2017; 19(6): 677–685. doi: 10.1093/ntr/ntw204

49. Johnson SL, Swerdlow BA, Treadway M, Tharp JA, Carver CS. Willingness to Expend Effort Toward Reward and Extreme Ambitions in Bipolar I Disorder. Clin Psychol Sci. 2017; 5(6): 943–951. doi: 10.1177/2167702617718181

50. Racine SE, Horvath SA, Brassard SL, Benning SD. Effort expenditure for rewards task modified for food: A novel behavioral measure of willingness to work for food. Int J Eat Disord. 2019; 52(1): 71–78. doi: 10.1002/eat.22999

51. Lopez-Gamundi P, Wardle MC. The cognitive effort expenditure for rewards task (C-EEfRT): A novel measure of willingness to expend cognitive effort. Psychol Assess. 2018; 30(9): 1237–1248. doi: 10.1037/pas0000563

52. Byrne KA, Ghaiumy Anaraky R. Strive to win or not to lose? Age-related differences in framing effects on effort-based decision-making. J Gerontol B Psychol Sci Soc Sci. 2020; 75(10): 2095-2105. doi: 10.1093/geronb/gbz136

53. Liang KY, Beaty TH, Cohen BH. Application of odds ratio regression models for assessing familial aggregation from case-control studies. Am J Epidemiol. 1986; 124(4): 678–683. doi: 10.1093/oxfordjournals.aje.a114441

54. Zeger SL, Liang KY. Longitudinal data analysis for discrete and continuous outcomes. Biometrics. 1986; 42(1): 121–130. doi: 10.2307/2531248

55. Strobel A, Beauducel A, Debener S, Brocke B. Eine deutschsprachige Version des BIS/BAS-Fragebogens von Carver und White. Zeitschrift für Differentielle und Diagnostische Psychologie. 2001; 22(3): 216-227. doi: 10.1024//0170-1789.22.3.216

56. Whiteside SP, Lynam DR. The five factor model and impulsivity: Using a structural model of personality to understand impulsivity. Pers Individ Dif. 2001; 30(4): 669–689. doi: 10.1016/S0191-8869(00)00064-7

57. Schmidt RE, Gay P, D’Acremont M, Van Der Linden M. A German adaptation of the UPPS impulsive behavior scale: Psychometric properties and factor structure. Swiss J Psychol. 2008; 67(2): 107–112. doi: 10.1024/1421-0185.67.2.107

58. Lejuez CW, Richards JB, Read JP, Kahler CW, Ramsey SE, Stuart GL, et al. Evaluation of a behavioral measure of risk taking: The balloon analogue risk task (BART). J Exp Psychol Appl. 2002; 8(2): 75–84. doi: 10.1037/1076-898X.8.2.75

59. Pleskac TJ, Wallsten TS, Wang P, Lejuez CW. Development of an automatic response mode to improve the clinical utility of sequential risk-taking tasks. Exp Clin Psychopharmacol. 2008; 16(6): 555–564. doi: 10.1037/a0014245

60. Janssen T, Larsen H, Peeters M, Boendermaker WJ, Vollebergh WAM, Wiers RW. Do online assessed self-report and behavioral measures of impulsivity-related constructs predict onset of substance use in adolescents? Addict Behav Rep. 2015; 1: 12–18. doi: 10.1016/j.abrep.2015.01.002

61. Orsini CA, Hernandez CM, Bizon JL, Setlow B. Deconstructing value-based decision making via temporally selective manipulation of neural activity: Insights from rodent models. Cogn Affect Behav Neurosci. 2019; 19(3): 459–476. doi: 10.3758/s13415-018-00649-0

62. Forbes EE, Dahl RE. Research Review: altered reward function in adolescent depression: what, when and how? J Child Psychol Psychiatry. 2012; 53(1): 3–15. doi: 10.1111/j.1469-7610.2011.02477.x

63. Chapman LJ, Chapman JP, Raulin ML. Scales for physical and social anhedonia. J Abnorm Psychol. 1976; 85(4): 374–382. doi: 10.1037/0021-843X.85.4.374

64. Zhang H, Harris L, Split M, Troiani V, Olson IR. Anhedonia and individual differences in orbitofrontal cortex sulcogyral morphology. Hum Brain Mapp. 2016; 37(11): 3873–3881. doi: 10.1002/hbm.23282

65. Johnson SL, Fulford D, Carver CS. The double-edged sword of goal engagement: consequences of goal pursuit in bipolar disorder. Clin Psychol Psychother. 2012; 19(4): 352–362. doi: 10.1002/cpp.1801

66. Ryba MM, Hopko DR. Gender Differences in Depression: Assessing Mediational Effects of Overt Behaviors and Environmental Reward through Daily Diary Monitoring. Depress Res Treat. 2012; 2012: 865679. doi: 10.1155/2012/865679

67. Khazanov GK, Ruscio AM, Forbes CN. The Positive Valence Systems Scale: Development and Validation. Assessment. 2020; 27(5): 1045–1069. doi: 10.1177/1073191119869836

Figure 1

Click here to access/download;Figure;fig1.eps

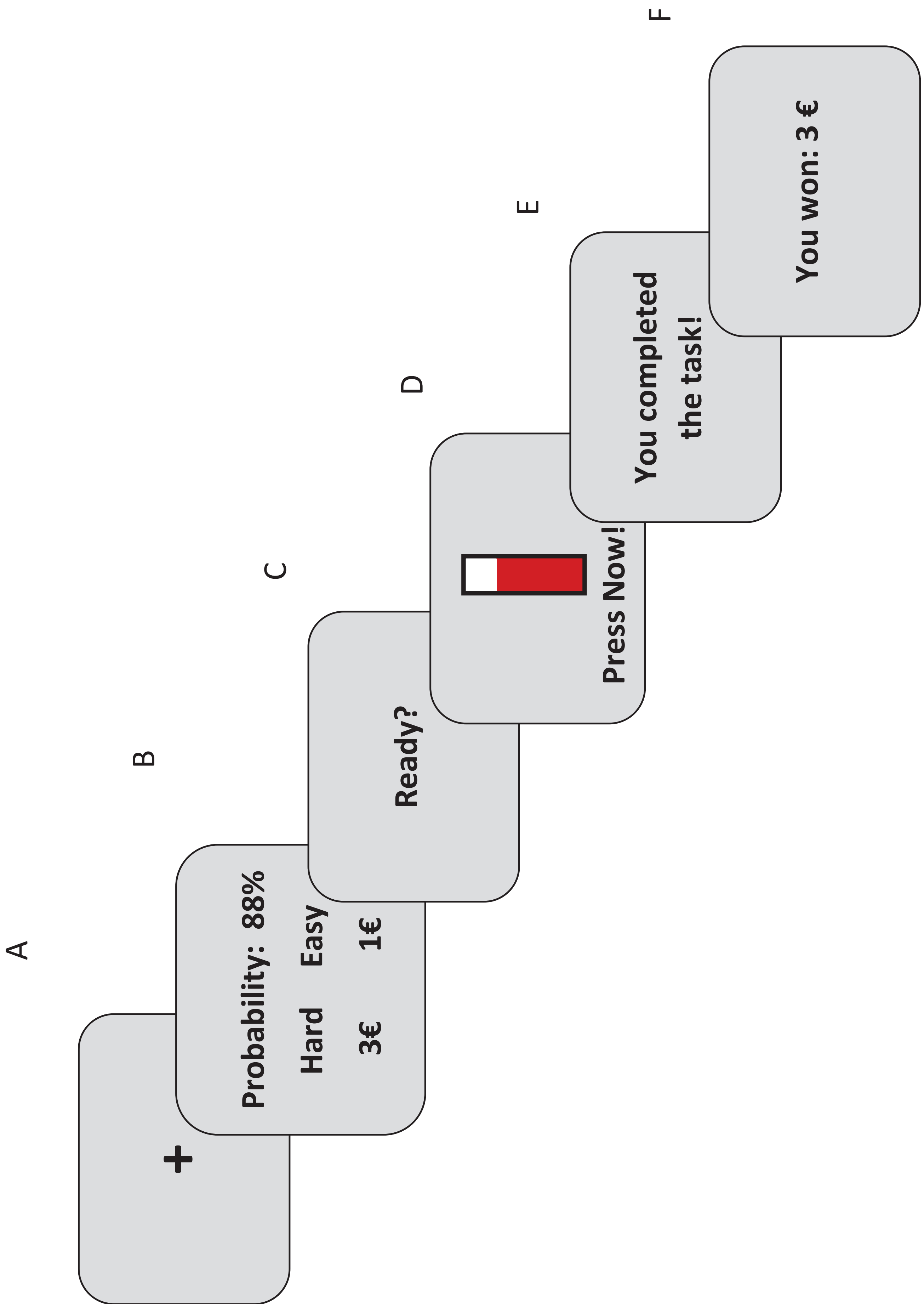

Figure 2 Click here to access/download;Figure;fig2.eps

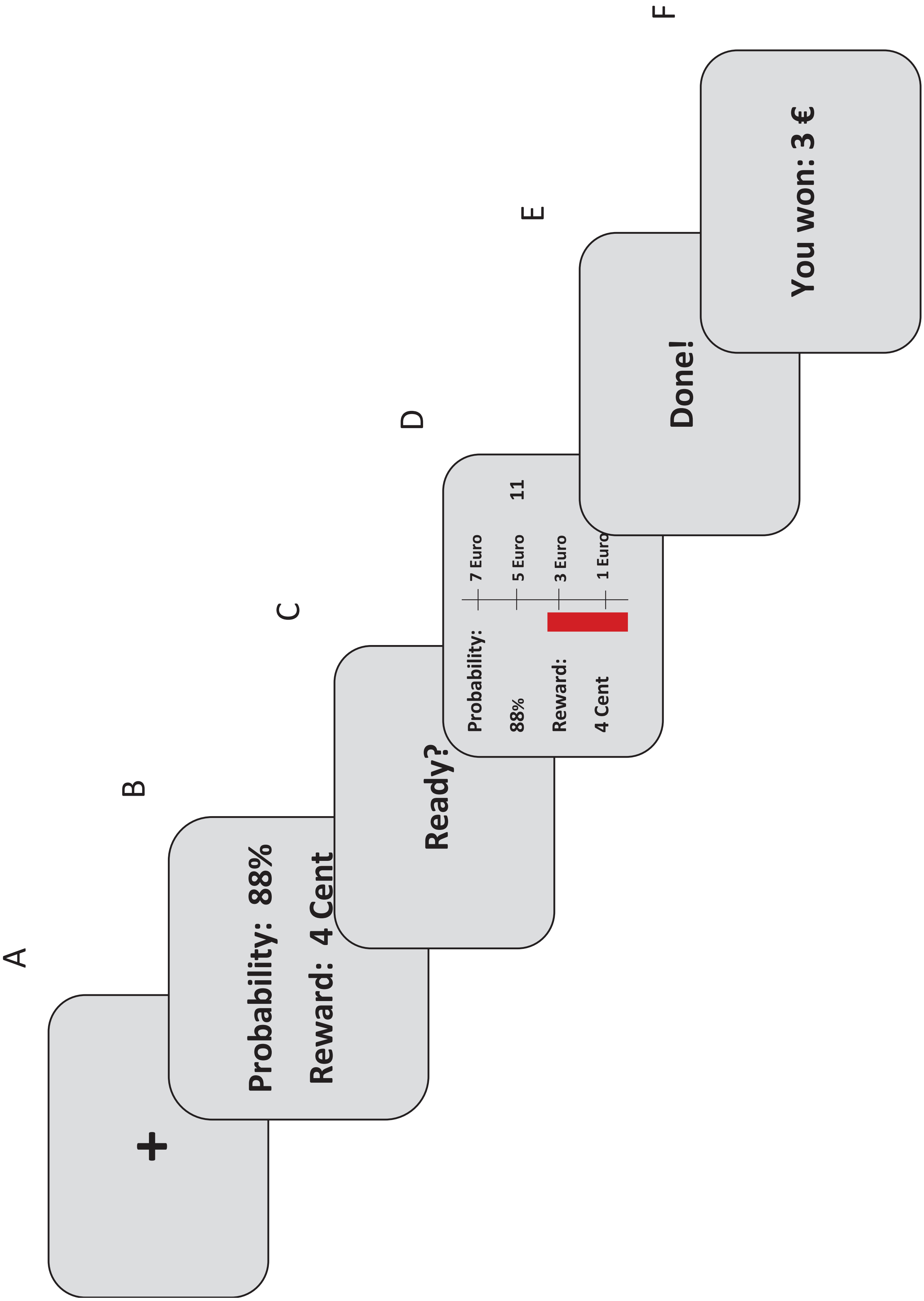

Figure 3

Click here to access/download;Figure;fig3.eps

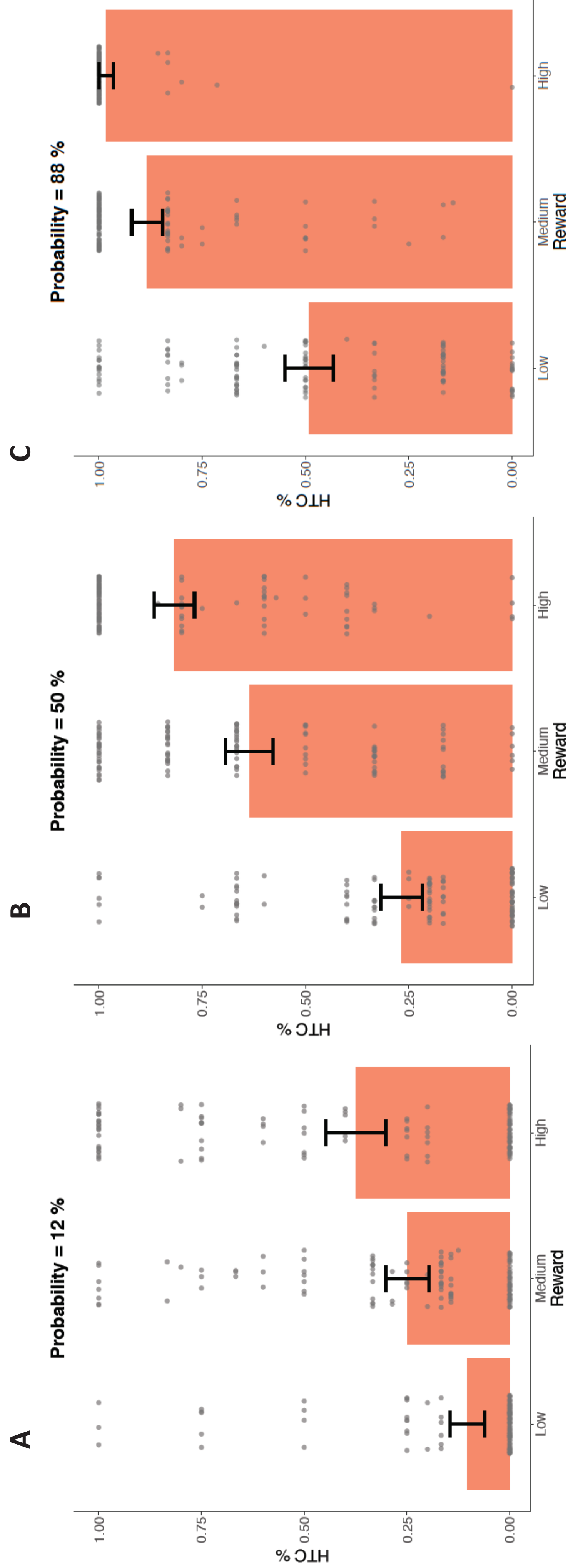

Figure 4

Click here to access/download;Figure;fig4.eps

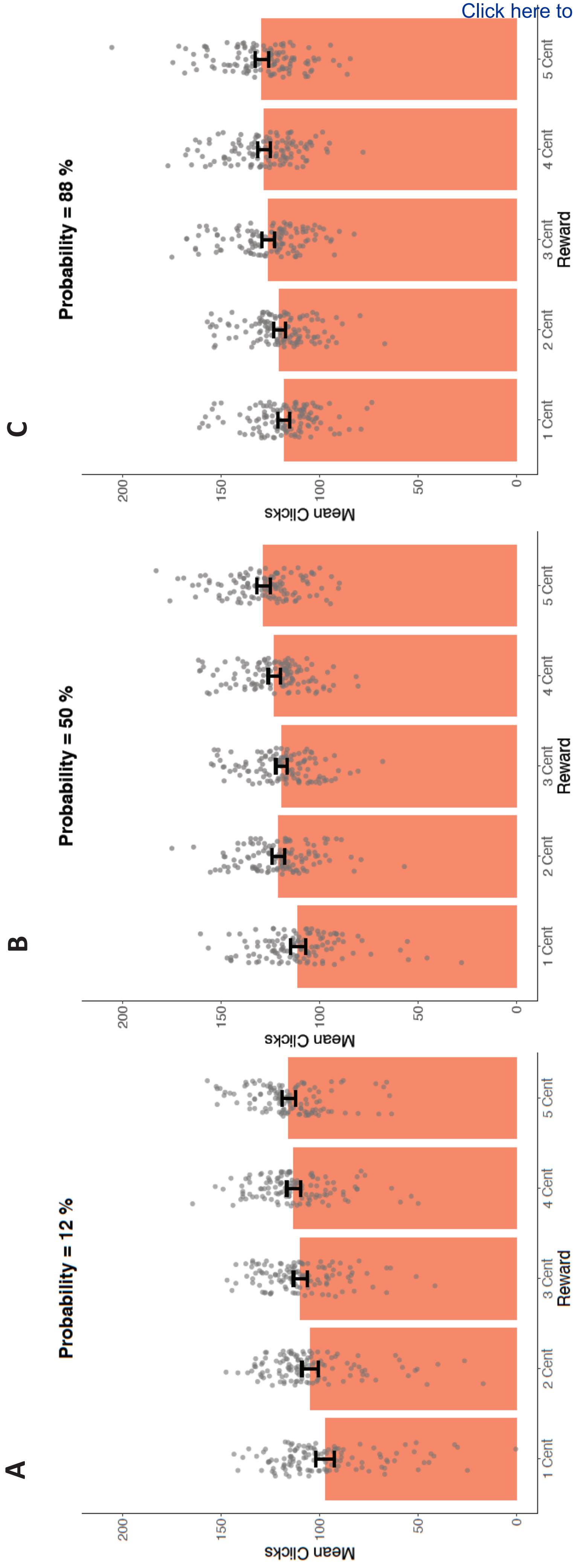

Figure 5

Click here to access/download;Figure;fig5.eps

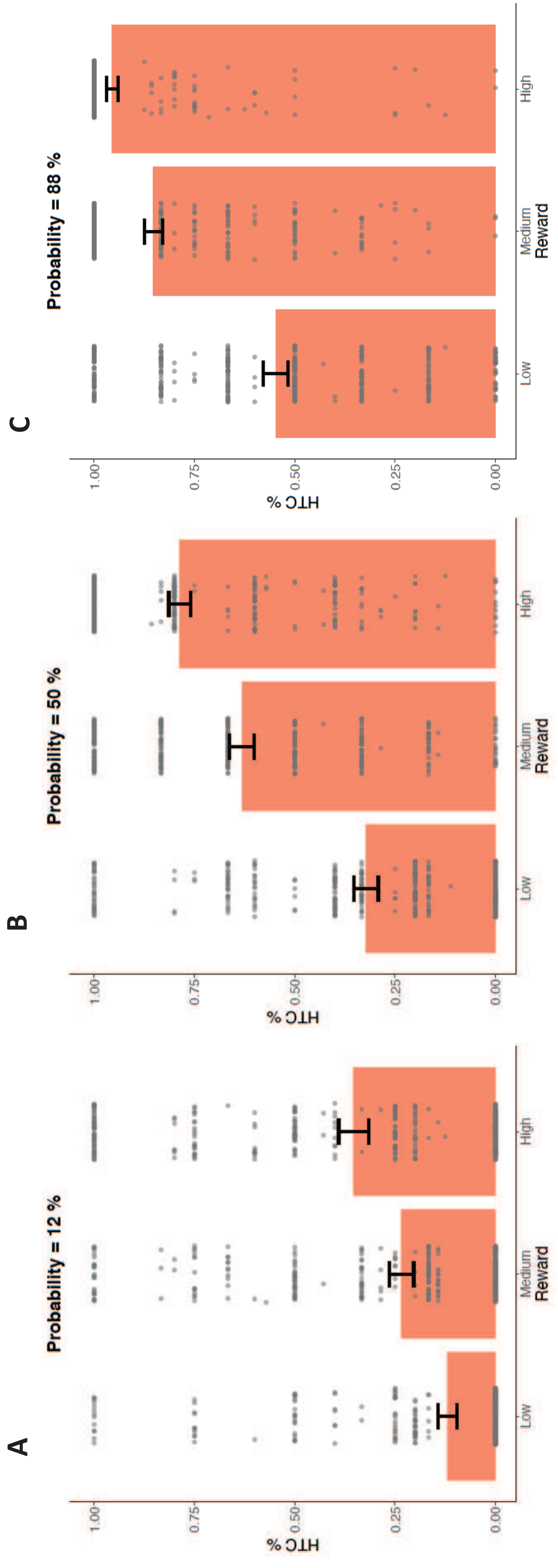

Figure 6

Click here to access/download;Figure;fig6.eps

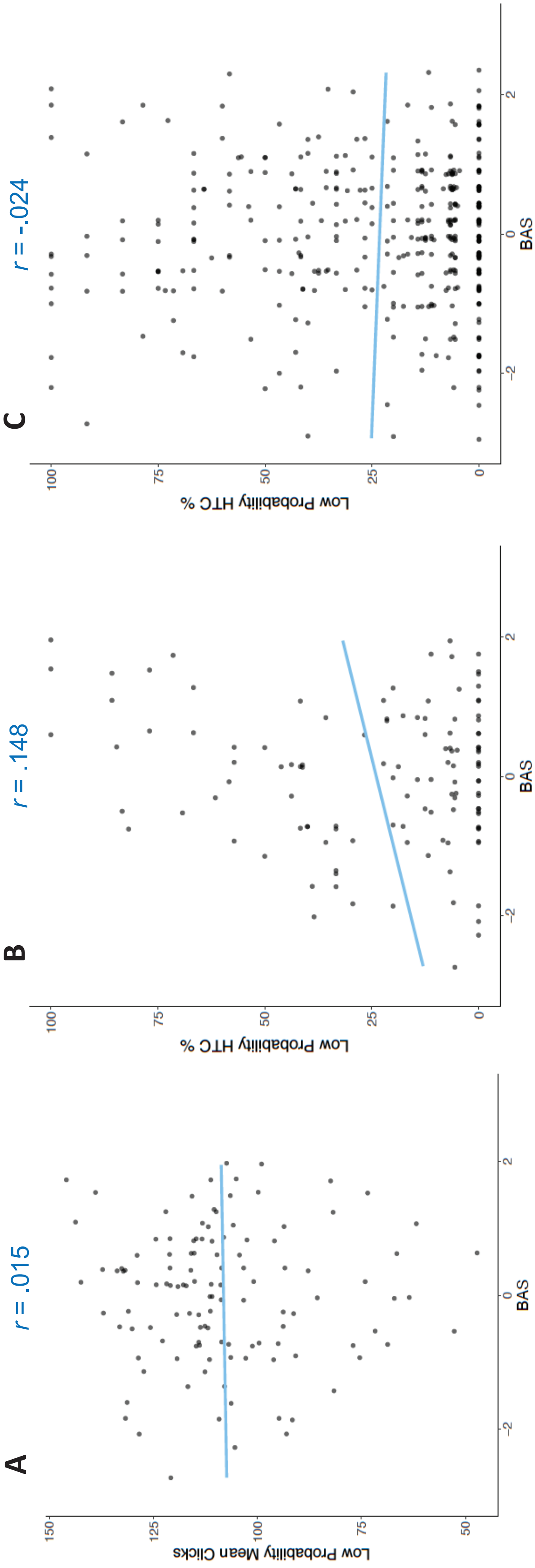

Figure 7

Click here to access/download;Figure;fig7.eps

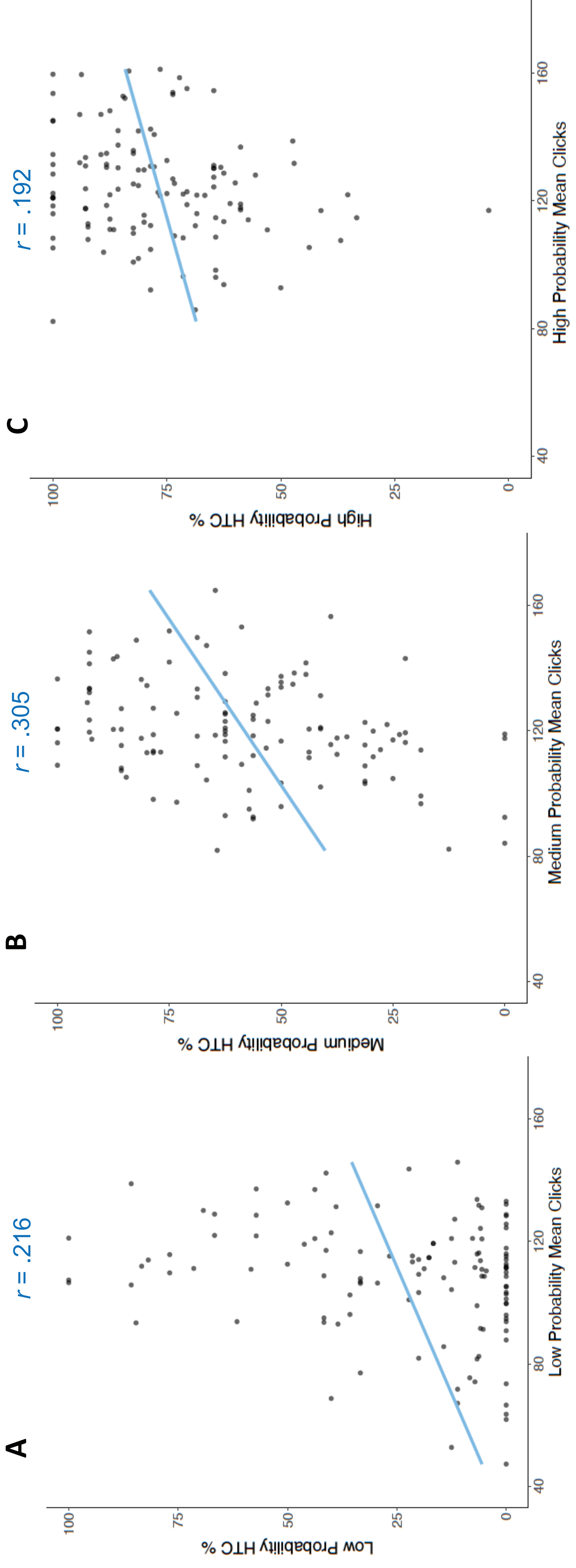

Examining the reliability and validity of two versions of the Effort-Expenditure for Rewards Task (EEfRT)

Hanno Andreas Ohmann[1*], Niclas Kuper[2], & Jan Wacker[1]

Universität Hamburg

Short title: Validation of two versions of the EEfRT

[1] Faculty of Psychology and Human Movement Science, Universität Hamburg, Hamburg, Germany

[2] Faculty of Psychology and Sports Science, Universität Bielefeld, Bielefeld, Germany

*Corresponding Author

hHanno_ohmann@hotmail.com ~~andreas.ohmann@uni-hamburg.de~~ (HAO)

Field Code Changed

Formatted: Default Paragraph Font, Font: (Default) +Body (Calibri), 11 pt, (Asian) Korean, (Other) German (Germany), Border: : (No border)

Keywords: Reliability; Validity; EEfRT; Approach Motivation; BAS

Abstract

The Effort-Expenditure for Rewards Task (EEfRT) has gained validity evidence from ~~various~~ several studies. However, various modifications have been applied to the original version, which have never been compared systematically. In Study 1, we tested 120 healthy participants to directly compare two versions of the EEfRT. In Study 2, we tested a larger sample of 394 healthy participants to further examine the original EEfRT. We replicated the split-half reliability of both task versions. However, self-reported personality traits (e.g., trait BAS) correlated with only some task performance parameters in Study 1, which did not replicate for the original EEfRT in Study 2. Our results indicate complex and sometimes inconsistent relations between different personality traits, task properties, and reward attributes. ~~However, trait BAS and trait anticipatory pleasure correlated positively with only some task performance parameters in Study 1 that did not replicate for the original EEfRT in Study 2. Our results indicate complex and sometimes inconsistent relations between different personality traits, task properties, reward attributes, and study designs.~~

1. Introduction

Harmon-Jones et al. [1] (p. 291) define approach motivation as "the impulse to go toward", which is based on internal state or trait - related processes and initiates behavior which is often (but not necessarily) associated with reaching specific goals. Approach motivation can therefore be seen as one of the main drivers of human behavior [2, 3]. The field of personality research related to approach motivation is often linked to Gray`s model of personality, which culminated~~accumulated~~ in his Reinforcement Sensitivity Theory (RST) [4, 5]. A variety of self-report measures are available for the assessment of personality traits related to sensitivity to rewards and punishment, ranging from the commonly used BIS / BAS scales [6] to the Temporal Experience of Pleasure Scale (TEPS) [7] measuring anticipatory pleasure and consummatory pleasure.

However, linking self-reported personality traits to behavioral measurements often leads to rather small correlations [8, 9]. Dang et al. [10] suggest that this might be a result of the poor reliability of many behavioral measures and the different response processes of these two types of measurement. Moreover, multiple behavioral tasks to measure approach motivation have been developed in a post-hoc manner and / or are based on animal models [11]. A majority of these tasks utilizes physical effort, which participants have to invest to gain rewards. Various kinds of physical efforts have been utilized. For example, hand-grip tasks assess willingness to expend effort by force exerted on the grip [12–14]. Such tasks are often adapted to a model based on effort discounting, which allows to measure the extent to

which the need for effort reduces preference for a given reward [15–17]. Similar tasks use button or lever pressing in a progressive ratio format [18–20]. Unfortunately, the majority of these tasks lack a comprehensive test of their reliability and validity. Comparing the psychometric properties of five different effort-based decision-making tasks in a clinical sample (schizophrenic patients) and a healthy control sample, the Effort Expenditure for Rewards Task (EEfRT) [21] exceeded the reliability of four other tasks [14, 22], making it a promising tool to investigate approach motivation.

The EEfRT is based on a concurrent choice paradigm developed by Salamone et al. [11] to explore effort-based decision making in rodents. The original EEfRT measures individual differences in human reward motivation by having participants decide between high cost/high reward (hard task, many clicks needed) and low cost/low reward (easy task, few clicks needed) behavioral options. The tendency to choose the hard task rather than the easy task has been shown to be associated with higher levels of approach motivation, as measured e.g., via personality trait questionnaires [23]. Specifically,~~Precisely,~~ trait BAS and trait anticipatory pleasure correlated positively with the percentage of hard-task-choices in trials with a low probability of rewards attainment in the original EEfRT. This indicates that higher approach motivation as measured by trait questionnaires is related to concrete behavior directed at gaining rewards in the EEfRT. Recently, these findings were replicated using a modified version of the EEfRT, showing that trait extraversion and trait BAS correlated positively with the mean number of clicks participants exerted in trials with a low probability of reward attainment [24]. However, Horan et al. [22], Anand et al. [25], as well as Kaack et al. [26] were not able to replicate these correlations using the original EEfRT, raising questions about the existence and magnitude of such associations.

According to Smillie [27], trait behavioral activation system (BAS) sensitivity should be predominantly related to reward sensitivity, while trait behavioral inhibition system (BIS)

sensitivity should be predominantly related to punishment sensitivity. One would thus expect ~~relatively specific~~ associations between behavior in the EEfRT~~task~~ and these personality traits. In particular, trait ~~— overall trait~~ BAS should be associated with increased task performance (due to greater sensitivity to rewards) and trait BIS with decreased task performance (due to higher sensitivity to the aversive quality of effort expenditure). The EEfRT has gained further support for its validity from various studies (see Table 1). For instance, h~~H~~ealthy participants' preference for the hard task has ~~i.e.~~ been shown to correlate with lower scores on negative affect, depressive symptoms, and anhedonia [21]. Furthermore, ~~it was shown that~~ left frontal anodal transcranial direct current stimulation (tDCS) over the dorsolateral prefrontal cortex increased participants' willingness to choose the hard task depending on reward attributes [28], which is in line with models associating left frontal brain activity with approach motivation [29, 30]. Wardle et al. [31] were the first to show that the EEfRT is also sensitive to pharmacological manipulation of dopamine (DA), as d-amphetamine increased participants' overall effort allocation. Furthermore, a low dosage of the D2 receptor blocker sulpiride, which is e.g., used in patients with depression and is believed to increase approach motivation, decreased participants' willingness to exert clicks in a modified version of the EEfRT [24].

~~Probably the most convincing~~Additional evidence for the validity of the EEfRT comes from patients suffering from impaired approach motivation: Patients with schizophrenia [32–34], first-episode psychosis [35], depression [36, 37], and autism [38] were less willing to choose the hard task as compared to healthy controls. Furthermore, the number of hard-task-choices was found to be negatively correlated with the severity of anhedonic symptoms in patients with schizophrenia [33] as well as in patients with depression [37]. Nguyen et al. [39] found in a large healthy sample of parents and their children that symptoms of psychopathology correlated with reduced effort allocation within the EEfRT. The EEfRT has also gained evidence on a neurophysiological level, as it has been shown to be related to left-

frontal cortical asymmetry in the resting state as well as during~~while~~ task performance [26, 40], which is believed to be a neural signature of approach motivation. Moreover, Huang et al. [41] found that the percentage of hard-task-choices was directly linked to the activity of the NAcc, which is a key structure of the human reward circuit, in both patients with schizophrenia and healthy participants. Overall, the literature thus shows intriguing support for the validity of the EEfRT.

However, there are also various limiting aspects (see Table 1). First, the number of studies reporting a significant link between the behavioral measurements within the EEfRT and self-reported~~ascribed~~ personality traits related to approach motivation is still small, ~~albeit~~ although many studies refer to this link ~~to prove the~~as validity evidence of the EEfRT. Second, the number of participants in studies which used the EEfRT has often been relatively small, resulting in low statistical power to detect effects sizes that can be expected in individual difference research [42]. Together with concerns about the replicability of psychological findings in general [43] as well as literatures relevant to this manuscript (e.g., trait approach motivation – frontal asymmetry link) [44], this highlights the risk of false positive results in previous studies. Third, the original EEfRT has been shown to be partly related to individual strategic behavior, which is not related to participants' actual approach motivation [28]. The modified version of the EEfRT [24] seeks to eliminate this limitation of the original task, but ~~so far lacks~~ requires additional data to document its ~~own~~ reliability and validity. Fourth, seemingly small differences in task properties and administration could have a great impact on task behavior. Despite this, several studies already modified the original EEfRT to fit different experimental settings (see Table 1).

Table 1. An overeview of selected studies using the EEfRT: Modifications, reliability, and validity (chronological order).

Formatted: Font: Bold, Not Italic

Formatted: Font: Bold

Study	N	Main dependent variable(s)	Main modifications applied to the original task	Modifications tested against original?	Reliability reported?	Validity test
Treadway et al. 2009 [21]	61	HTC	-	-	No	Self-report measures (Anhedonia, depression)
Wardle et al. 2011 [31]	17	HTC	Repeated--measure--design	No	No	Effects of d-amphetamine
Damiano et al. 2012 [38]	58	HTC, response change	Removed time-limit for task choice	No	No	Patients with ASD (n= 20) VS healthy controls (n = 38)
Wardle et al. 2012 [45]	23	HTC, response speed	Practice session, repeated measure	No	No	Effects of caffeine
Fervaha et al. 2013 [32]	32	HTC	Individual calibration of required clicks	No	No	Patients with SCZ (n= 16) VS healthy controls (n = 16)
Barch et al. 2014 [33]	98	HTC	Removed low probability trials	No	No	Patients with SCZ (n= 59) VS healthy controls (n = 39); self-report measures (anhedonia, depression)
Yang et al. 2014 [37]<br>- study 1	99	HTC	Reduced number of required clicks; fixed the possible monetary rewards	No	No	high BDI-score (n =43) VS low BDI score (n =56); self-report measures (anhedonia; pleasure)
- study 2	87	HTC	" Reduced number of required clicks; fixed the possible monetary rewards"	No	No	Patients with MDD (n = 41) VS remitted MDD (n= 41)

Note. ASD = Autism spectrum disorder; BAS = Behavioral Activation System; BIS= Behavioral Inhibition System BDI = Beck Depression Inventory; BMI = Body-mass-index; HTC = Hard-task-choices; MDD = major depressive disorder; SCZ = schizophrenia

Table 1 (Continuation). An overview of selected studies using the EEfRT: Modifications, reliability, and validity (chronological order).

Study	N	Main dependent variable(s)	Main modifications applied to the original task	Modifications tested against original?	Reliability reported?	Validity test
Hughes et al. 2015 [46]	51	HTC	Paid 10% of total winnings	No	No	Association with frontal asymmetry (resting state)
Geaney et al. 2015 [23]	97	HTC	Mood induction; paid 10% of total winnings	No	No	Self-report measures (anhedonia, pleasure, BAS)
Gilman et al. 2015 [47]	50	HTC; reaction time	Pictures of peers (social influence)	No	No	Effects of different social influence conditions
Reddy et al. 2015 [14]	134	Reward sensitivity	Repeated--measure-design; individual calibration of required clicks; no low probability trials	No	**Yes (retest)**	Patients with SCZ (n= 94) VS healthy controls (n = 40); comparison with four other tasks
Horan et al. 2015 [22]	134“	Reward sensitivity “	Repeated-measure-design; individual calibration of required clicks; no low probability trials “	No “	**"Yes (retest)**	Self-report measures (SCZ symptoms; motivation; BIS/BAS)
Hughes et al. 2017 [48]	128	HTC	Repeated-measure-design; shorter task selection (3s)	No	No	Abstinent smokers (n = 61) VS former smokers (n = 67); self-reported reward enjoyment
Johnson et al. 2017 [49]	50	HTC	Fixed probability (50%), tThree bonus trials added (no effort needed)	No	No	Patients with remitted bipolar disorder; self-reported life ambitions
Lopez-Gamundi & Wardle 2018 [51]	60	HTC	Cognitive effort version	**Yes**	No	Comparison of cognitive VS original version

Note. ASD = Autism spectrum disorder; BAS = Behavioral Activation System; BIS= Behavioral Inhibition System BDI = Beck Depression Inventory; BMI = Body-mass-index; HTC = Hard-task-choices; MDD = major depressive disorder; SCZ = schizophrenia

Formatted: Font: Bold, Not Italic

Formatted: Font: Bold

Table 1 (Continuation). An over~~e~~view of selected studies using the EEfRT: Modifications, reliability, and validity (chronological order).

Study	N	Main dependent variable(s)	Main modifications applied to the original task	Modifications tested against original?	Reliability reported?	Validity test
Ohmann et al. 2018 [28]	60	HTC	Repeated-~~-~~measure-~~-~~design; paid 5% of the total winnings	No	No	Effects of anodal tDCS stimulation
Racine et al. 2019 [50]	63	HTC	Food portions instead of monetary reward	No	No	Comparison of participants with different degree of b~~B~~inge-eating symptoms; BMI
Nguyen et al. 2019 [39]	2259	Reward sensitivity	No probabilities of reward attainment for kids	No	No	Comparison of parents (n= 1044) and children (n= 1215) for psychopathic symptoms
Byrne & Ghaiumy Anaraky 2019 [52]	94	HTC	Addition of “loose” trials; temporal delay after easy trials	No	No	Comparison of older (n= 46) and younger adults (n= 48)
Ohmann et al. 2020 [24]	203	Button presses	No task selection, instead increased potentiali~~ng~~ monetary gains~~win~~ with each click	No	**Yes (split-half)**	Effects of sulpiride; s~~S~~elf-report measures (Big Five; BIS/BAS)
Kaack et al. 2020 [26]	49	HTC	Additional “offer”- screen (5s) prior to choice screen; best performing student would receive $100 grocery store voucher	No	No	~~Effects of~~Associations with frontal asymmetry (resting and task states); s~~S~~elf-report measures (BIS/BAS)

Note. ASD = Autism spectrum disorder; BAS = Behavioral Activation System; BIS= Behavioral Inhibition System BDI = Beck Depression Inventory; BMI = Body-mass-index; HTC = Hard-task-choices; MDD = major depressive disorder; SCZ = schizophrenia

Formatted: Font: Bold, Not Italic

Formatted: Font: Bold

Just to name a few examples: Yang et al. [37] reduced the number of required clicks and fixed the possible monetary rewards to reduce the complexity of the task for depressive patients. This design was also used by Huang et al. [41] to make the EEfRT suitable for functional brain imaging. Barch et al. [33] completely removed the low probability of reward attainment category, Damiano et al. [38] removed the time limit when participants select either the easy or the hard task, Fervaha et al. [32] calculated an individual number of required clicks before the actual task based on motoric abilities, and Byrne & Ghaiumy Anaraky [52] introduced "loss trials", in which choosing the easy task leads to potentially higher monetary loss compared to the hard task. Other authors exchanged the monetary rewards of the EEfRT, e.g., by using food portions in a study with patients suffering from binge-eating [50].~~,~~ A different study~~or~~ added a social influence aspect while participants chose between the easy and hard task of the EEfRT by adding pictures of "peers" and their respective choices [47]. Despite these various modifications applied to the EEfRT so far, surprisingly little is known about the effect of such modifications as almost no study directly compared different versions of the EEfRT within one experimental design. A commendable exception is a study by Lopez-Gamundi & Wardle [51], who compared the original EEfRT to a modification which uses cognitive effort (set-switching-task; C-EEfRT) instead of the physical effort within the original task (clicks). Although participants perceived the C-EEfRT as more difficult, participants did choose the hard ~~-~~task more often compared to the original EEfRT. Furthermore, the relationship between the effort allocation within both task versions was only moderate, indicating distinct processes for both kinds of effort~~s~~ when participants decide to allocate effort to gain a possible reward.

~~Bearing these limitations in mind, we here seek to analyze the reliability and validity of the original EEfRT and a modified EEfRT in two~~ ~~2~~ ~~different study designs and try to deepen the understanding of the link between self-ascribed~~ reported ~~personality traits and behavioral task measures. Study 1 aims at directly comparing the validity and reliability of~~

Formatted: Outline numbered + Level: 2 + Numbering Style: 1, 2, 3, … + Start at: 1 + Alignment: Left + Aligned at: 0" + Indent at: 0.25"

~~two versions of the EEfRT. Study 2 aims to replicate the reliability and validity of the original EEfRT by making use of a large sample to further increase statistical power.~~

1.1 ~~1.1~~ Present study~~Hypotheses~~

Bearing these limitations in mind, we here seek to analyze the reliability and validity of the original EEfRT and a modified EEfRT in two different study designs and try to deepen the understanding of the link between self-reported personality traits and behavioral task measures. Study 1 aims at directly comparing the validity and reliability of two versions of the EEfRT. Study 2 aims to replicate the reliability and validity of the original EEfRT by making use of a large sample to further increase statistical power.

Formatted: Font: (Default) Times New Roman, 12 pt, Font color: Text 1

Formatted: Normal, None, Indent: First line: 0.25", Space Before: 0 pt, Line spacing: Double, Widow/Orphan control, Hyphenate

Formatted: Font: Not Bold

1.1.1 Reliability of the EEfRT

Based on the promising results regarding the retest~~-~~reliability found in previous studies for the original EEfRT [14, 22] and likewise promising results for the split-half reliability of the modified EEfRT [24], we expected both versions of the task to show overall good (Rel > .80)~~-~~ split-half reliability in both studies. We further examined the internal consistency of all questionnaire measures used in both studies.

1.1.2 Validity of Basic Task Variables

In line with previous research, we expected the reward attributes (reward magnitude and probability of reward attainment) to be positive predictors of the percentage of hard-task-choices (original EEfRT) and mean number of clicks (modified EEfRT), whereas we expected trial number (i.e., an indicator of fatigue) to be a negative predictor of the percentage of hard-task-choices (original EEfRT) and mean number of clicks (modified EEfRT). ~~-~~GEE models (generalized estimating equations) [53, 54] have been the main analysis strategy for the examination of basic task variables in previous work on the EEfRT [21, 23, 24]. Therefore, we also applied GEE models to test for the effects of the above-mentioned basic task variables.

1.1.3 Personality Correlations

As Gignac & Szodorai [42] stated, correlations of r = .30 should be considered as rather ~~high~~ large correlations in the field of individual differences. Applying these standards, previous studies using the original EEfRT [23] as well as the modified version of the EEfRT [24] found medium to large correlations for the percentage of hard-task-choices (original EEfRT) ~~)~~ and the mean number of clicks (modified EEfRT) ~~)~~ in trials with low probability of reward attainment with trait BAS (*r* ranging from .212 to .361) and trait anticipatory pleasure (*r* = .251). We seek to replicate these correlations in both studies. Furthermore, as some studies using the EEfRT focused on the impact of reward magnitude, as well as the differences between low and high reward trials [14, 22], indicating "reward sensitivity", we exploratorily analyzed the correlations between traits with the percentage of hard-task-choices (original EEfRT) and mean number of clicks (modified EEfRT) depending on reward magnitude. Therefore, we examined the correlations between trait measures~~s~~ and task performance in all probability of reward attainment categories as well as in all reward magnitude categories and difference scores between these trial categories in an exploratory fashion. To evaluate the discriminant validity of both tasks, we further tested for the associations with distinct constructs, which we expected to not correlate with effort allocation on both tasks - namely risk-taking (behavioral measure) and impulsivity (self-reported trait measure).

1.1.4 Secondary Analysis

As Study 1 includes both versions of the EEfRT in one experimental design, we seek to estimate the correlations between both task versions in an exploratory fashion. As there are no previous studies to base our hypothese~~i~~s on, we only hypothesize a significant positive correlation between both versions, as both tasks should measure the same construct: approach motivation. We further analyzed participants' self-reported strategy usage and motivation and their linkage to task performance in Study 1 to further test the validity of both task versions.

2. Methods

2.1 Study 1

2.1.1 Participants

We recruited physically and psychologically healthy participants (78.~~,~~3% female) aged 18 – 35 (*M* = 24.~~,~~97; *SD* = 4.~~,~~14) using online notice boards and flyers at a local university. Out of 125 recruited participants, 5 had to be excluded for different reasons (1 participant was not able to understand the instructions; 2 participants did not understand the task; 2 participants had missing values due to technical failure). Thus, the final sample consisted of 120 participants. In line with previous studies [23, 24], we expected correlations around ρ = .30 (i.e., large correlations according to Gignac & Szodorai [42]). As intended, statistical power was therefore >.80 (exact 1-β = .92) to detect correlations of $\rho = .30$ ($\alpha = .05$). Participants received monetary compensation (10€ per hour) and were told that they could gain additional money based on their collected rewards from both versions of the EEfRT (5% of the virtually collected money) and the BART (one cent per 4 pumps in successful trials) which was paid to participants at the end of the study. The authors assert that all procedures contributing to this work comply with the ethical standards of the relevant national and institutional committees on human experimentation and with the Helsinki Declaration of 1975, as revised in 2008. The study has been approved by the local Ethics Committee of the University of Hamburg. Exclusion criteria comprised the intake of any kind of prescribed medication over the last three months, the consumption of illegal drugs over the last four weeks, neurological or medical conditions, and the presence of any mental disorders (in particular affective~~ –~~, somatoform~~ –~~, psychotic~~ –~~, anxiety~~ –~~, eating~~ –~~, and adaptive disorders, as well as substance use disorders)~~mental disorders triggered by drug abuse)~~.

2.1.2 Randomization

As we intended to compare both versions of the EEfRT (original / modified) and their relation to measures of personality traits and risk-taking behavior, we randomized the order of both tasks in a counterbalanced fashion to ensure that the order of both tasks did not influence effort allocation. Participants were randomly assigned to one of both conditions at the start of part two of our study.

2.1.3 Procedure

Participants who fit the inclusion criteria were provided with information about the study via email. Participants gave their informed consent in written form via email~~E-Mail~~ and then received an individual link to the first part of the study. The first part of the study comprised an online-survey lasting about 30 minutes, in which participants filled out a ~~small~~ series of questionnaires, including demographic information and German versions of the BIS/BAS scales [6, 55], the TEPS [7], and the UPPS [56, 57]. Participants who completed the first part of the study were invited into our lab. After arriving at our lab, participants personally signed the informed consent~~,~~ which they sent before via E-Mail. Afterwards, the participants completed a series of computer tasks lasting about 60 minutes. All participants started by completing a test of their motoric abilities. Participants then completed either the original or the modified version of the EEfRT according to the assigned condition. Afterwards, they completed the Balloon Analogue Risk Task~~BART~~ (~~Balloon Analogue Risk Task~~BART) [58, 59]. The BART ~~has been applied~~was always completed in the middle of the study to avoid motoric fatigue of the participants throughout the whole study. The BART~~is~~ was followed by another test of~~ their~~ motoric abilities and the complementary version of the EEfRT, which participants did not complete before. Finally, participants completed a small set of questions, including questions about aspects that might have influenced their effort allocation within each version of the task (reward magnitude, probability of reward, fatigue, resting one's fingers) and their motivation to earn additional money throughout the whole study using five-point Likert-scales ranging from "not at all" (1) to "a lot" (5).

2.1.4 Original EEfRT

We used a translated (German) version of the EEfRT [21], which was programmed using Presentation software 17.1 (Neurobehavioral Systems Inc, San Francisco). Every participant completed one 15–minute block of the EEfRT with their dominant hand. Participants were instructed to win as much virtual money as possible throughout the block. In short, participants need to choose between an easy, low-reward task and a hard, high-reward task in every trial (see Fig 1 for a schematic illustration). The reward for the easy task is fixed to 1 € while the reward for the hard task is variable (ranging between 1.21 € and 4.30 €). To further manipulate the value of each reward, the probability of reward attainment also varies [either 12% (low), 50% (medium~~iddle~~) or 88% (high)], which is presented at the start of each trial alongside the reward values. The easy task requires participants to press the space button 30 times ("clicks") in 7 seconds with their index finger. The hard task requires participants to press the space button 100 times ("clicks") in 21 seconds with their pinkie finger. While pressing the spacebar, a visually presented white bar gradually fills up with red color. After each trial, the participants are informed about the amount of money they won during the trial. The order of trials, as well as the probability of reward attainment and the reward magnitudes are not randomized between participants, but pre-assigned for each trial. This is done to rule out random feedback differences between participants.

Fig 1. Schematic illustration of one trial of the original EEfRT.

Formatted: Font: Not Italic

A fixation cross (1s, A) is followed by a screen showing probability of reward attainment and reward magnitude for the easy and the hard task (B), lasting until the participant made a choice which task to complete but no longer than 5s. Then, after presentation of a ready – screen (1s, C) the main screen for the trial showing a red bar that fills with each button press is presented until the task is completed or until the trial time is over (D). Finally, task completion is signaled (2-s, E) and a feedback screen shows the amount of money won (2s, F).

2.1.5 BART

To test participants` risk-taking behavior, we also assessed the BART [58, 59]. In this task, participants are presented a picture of a balloon, and instructed to inflate this balloon. Inflating the balloon increases the size of the balloon on screen and the associated reward. However, overinflating the balloon would result in the balloon bursting accompanied by an aversive auditory sound. Bursting of the balloon causes the participant to lose the entire reward of that trial. Each of 30 balloons have a different predetermined bursting point, on a scale of 1 to 128 (pumps). Participants are instructed that the average number of inflations that causes the balloon to burst is 64 and that they would gain one Cent per 4 pumps, only in successful trials (balloon did not burst). In line with previous studies, we used the automatic response procedure of the BART [59, 60], in which the participants could immediately select the intended number of pumps for that specific trial and receive immediate feedback as they watch the balloon inflating. Also, risk taking scores were calculated as the mean of the number of pumps across all balloons regardless of the burst event [59], unlike in the original BART [58].

2.1.6 Modified EEfRT

Additionally, we used a modified version of the original EEfRT [21], which was programmed using Presentation software 17.1 (Neurobehavioral Systems Inc, San Francisco) and has been used in one previous study [24]. Ohmann et al. [28] found that using the original EEfRT comes with a major downside: At least some participants understand that choosing the hard task is often lowering the possible overall monetary gain as the hard task takes almost 3 times as long as the easy task and the overall duration of the task is fixed. Hence, at least some participants' choices are partly based on a strategic decision and less on approach motivation per se. To overcome this downside, the original EEfRT was modified. First, the number of trials (2 blocks x 15 trials = 30 trials) and the duration of each trial (= 20 seconds)

was fixed. Participants used their dominant hand for both blocks in the present study. Second, the original choice-paradigm was changed. Participants no longer choose between an easy and a hard task. As in the original task, the value of each reward varies, and participants are informed about this at the start of each trial. But instead of presenting specific reward magnitudes, participants are now presented with a reward magnitude per click (1 /2 / 3 / 4 / 5 cents per click). Thus, participants are able to increase the total possible monetary gain in each trial with each click. In accordance with the original task design, the probability of reward attainment also varied [either 12% (low), 50% (medium~~iddle~~) or 88% (high)], which is presented at the start of each trial alongside the reward value per click. Participants were instructed to win as much virtual money as possible throughout the task, however they were free to choose the amount of effort they exerted in each trial. Critically, the only way to increase the possible monetary gain is to increase the number of clicks in each trial. The task itself is designed close to the original EEfRT but comes with some modifications to prevent the use of strategies (see Fig 2). While pressing the spacebar, a visually presented red bar gradually grows. A scale (€) was implemented, so that the participants can always see how much their button-presses (“clicks”) increase their possible monetary gain. Furthermore,~~ we~~ the information on the reward magnitude per click and the probability of reward attainment is presented throughout the whole trial alongside a countdown (20 seconds) to increase participants’ awareness of these parameters. After each trial, participants are informed about the amount of money they won during the trial. The order of trials, as well as the probability of reward attainment and the reward magnitudes per click are not randomized between participants, but pre-assigned for each trial. This is done to rule out random feedback differences between participants.

Fig 2. Schematic illustration of one trial of the modified EEfRT.

Formatted: Font: Not Italic

A fixation cross (1s, A) is followed by a screen showing probability of reward attainment and reward

magnitude per click for 3s (B). Then, after presentation of a ready – screen (1s, C), the main screen for the trial showing a red bar that grows with each click is presented alongside a scale, indicating the current monetary gain and a countdown (20s, D). Finally, task completion is signaled (1.,5s, E) and a feedback screen shows the amount of money won (2s, F).

Formatted: Space After: 8 pt

2.1.7 Motoric Abilities

Participants with greater motoric ability exert more clicks throughout the modified version of the EEfRT [24] and studies calibrating an individual number of clicks to succeed within the original EEfRT suggest that participants with higher motoric abilities might also choose the hard task more often in the original version [14, 32], which does not reflect their actual approach motivation. Therefore, we included 10 motoric trials to test participants' motoric abilities before each version of the EEfRT. Within these motoric trials, participants were instructed to press the spacebar as often as possible within 20 seconds. Critically, participants were not able to gain any rewards in these trials and visual feedback was reduced to a countdown and a display of the number of clicks they exerted. Participants' individual motoric abilities were operationalized as maximal clicks in motoric trials (MaxMot) and included in our statistical models.

2.1.8 Data Analysis

Aggregated data were further analyzed using the SPSS 26.0 software* (Chicago, IL, USA). First, we examined the split-half reliability of different task parameters. To this end, we split the dataset into the first and second temporal half of trials for each individual in the original task. In the modified task, split-half reliability was estimated across the two blocks. Furthermore, we calculated internal consistencies of the questionnaire measures used.

To test for the effects of basic predictors on the percentage of hard-task-choices (original EEfRT) and on the mean number of clicks (modified EEfRT), we used GEEs. GEEs are marginal models that allow for robust parameter estimation despite correlated residuals,

e.g., due to the clustering of trials within participants [53,54] ~~[53, 54]~~. ~~Models were fit using an independent working correlation matrix.~~ Crucially, GEEs~~they~~ are consistent and provide appropriate robust standard errors even when the correlation matrix for the residuals is specified incorrectly [54]. Models were fit using an exchangeable working correlation matrix. Given that our dependent variable (hard~~-~~-task~~-~~-choices) was binary, we implemented models using the binomial distribution with a logit link. For the modified task (dependent variable: number of clicks), a gaussian distribution was assumed. All GEE models included the factors trial number, probability (categorical), reward magnitude, and the interaction of probability x reward magnitude (often referred to as "expected value"). Moreover, participants' individual motoric abilities were included in all GEE models.

Pearson correlations were computed between ~~trait personality variables~~self-reported personality traits (BIS/BAS/TEPS/UPPS), the number of pumps within the BART, the percentage of hard-task-choices within the original EEfRT, and the mean number of clicks within the modified EEfRT, separately for each probability of reward attainment category (low/medium/high), for each reward magnitude category (low/medium/high), as well as for difference scores between these categories. Within the original EEfRT, we formed these categories in line with previous studies [46]; low reward magnitude: <2,30 Euro, medium reward magnitude: 2,31-3,29 Euro, high reward magnitude: >3,30 Euro. For the modified EEfRT, we calculated analogue categories: Low reward magnitude: 1 or 2 Cent per click, medium reward magnitude: 3 C~~c~~ent per click, high reward magnitude: 4 or 5 Cent per click. Further Pearson correlations were calculated between both versions of the EEfRT for each probability of reward attainment category, each reward magnitude category, as well as between both versions of the EEfRT and follow-up questions regarding individual strategies and motivation. Datasets and syntax can be found at: https://osf.io/35k2w/?view_only=1f315859028c471787a96e153f5ac43a.

Formatted: English (United States)

Formatted: English (United States)

Field Code Changed

2.2 Study 2

2.2.1 Participants

We recruited physically and psychologically healthy, right-handed participants (68.2% female) aged 18 – 50 (*M* = 25.74; *SD* = 5.37) using online notice boards and flyers at various universities. Out of 409 recruited participants, 15 had to be excluded for different reasons (11 participants consumed illegal drugs or hormones within the last 12 months, 1 subject already knew the EEfRT task, 1 subject was ambidextrouser,1 subject had missing values for the UPPS, and for 1 subject it was not possible to collect a blood sample). Thus, the final sample consisted of 394 participants. In accordance with Gignac & Szodorai [42], we applied a more conservative criterium than in Study 1 considering the discrepancy between published correlations and those correlations we found in our lab -and expected only medium-sized correlations. As intended, statistical power was therefore >.80 (exact 1-β = .98) to detect correlations of *ρ* = .20 (*α* = .05). Participants received monetary compensation (10€ per hour) and were told that they could gain additional money based on their collected rewards from the original EEfRT (5% of the virtually collected money). At the end of the study, all participants received 5€ to ensure equity. This amount was always higher than the 5% of the virtually collected money. The authors assert that all procedures contributing to this work comply with the ethical standards of the relevant national and institutional committees on human experimentation and with the Helsinki Declaration of 1975, as revised in 2008. The study has been approved by the local Ethics Committee of the Medical Chamber of Hamburg. Exclusion criteria comprised the regular intake of any kind of prescribed medication, consumption of illegal drugs over the last 12 months, neurological or medical conditions, and the presence of any mental disorders - (in particular affective-,- somatoform,-,- psychotic-,- anxiety-,- eating-,- and adaptive disorders, as well as substance use disordersmental disorders triggered by drug abuse), high consumption of alcohol (more than 15 glasses per week), nicotine (more than 15 cigarettes per week) or caffeine (more than 4 cups per day),

and pregnancy (tested onside).

2.2.2 Procedure

As the original EEfRT and ~~both~~ relevant questionnaires (BIS/BAS, UPPS) were deployed as part of a larger study, we will focus only on the most relevant information. A complete description of the procedure will be provided elsewhere. The order of tasks and questionnaires was not randomized and in contrast to Study 1, no motoric trials were included. Participants who fit the inclusion criteria were invited into the lab once. After arriving at the lab and giving their informed consent in written form, participants completed a large series of questionnaires, including the German BIS/BAS [6, 55] and the UPPS [56, 57], as well as a series of computer tasks, including the original EEfRT [21]. The overall experiment lasted for about 4.~~,~~5 hours. The EEfRT took place approximately 4 hours after the start of the experiment.

2.2.3 Original EEfRT

The procedure of the original EEfRT was identical to experiment 1 (see 2.1.4 and Fig 1).

2.2.4 Data Analysis

Statistical analysis for the original EEfRT was identical to experiment 1 (see 2.1.8), with the following exceptions: As no motoric trials were included, individual motoric abilities were removed as a factor from the GEE model. In accordance with Study 1, Pearson correlations were computed between ~~trait personality variables~~self-reported personality traits (BIS/BAS/UPPS) and the percentage of hard-task-choices within the original EEfRT for each probability of reward attainment (low/medium/high) and each category of reward magnitude (low/medium/high), as well as difference scores between these categories. Datasets and syntax can be found at: https://osf.io/35k2w/?view_only=1f315859028c471787a96e153f5ac43a.

Field Code Changed

3. Results

3.1 Reliability

We examined the internal consistency of all questionnaires in both studies by estimating Cronbach‘s Alpha. Most questionnaires showed moderate to good internal consistency (see Table 2). ~~However, two subscales of the BIS/BAS scales (Fun Seeking; α = 0.56 / Reward Responsiveness; α = 0.53) showed insufficient reliability in Study 2.~~ We then calculated the reliability of the EEfRT by estimating ~~the~~ split-half co~~reliabilit~~rrelations~~y~~ and applying Spearman-Brown corrections to the resulting estimates. Note however, as we introduced motoric trials within Study 1, we compared ~~both~~ reliabilities un~~(~~adjusted and adjusted for motoric abilities, respectively (see~~for motoric abilities and non-adjusted, see~~ Table 2). Therefore, we calculated the reliability of the percentage of hard-task-choices and clicks after residualizing them on~~for~~ motoric abilities (operationalized as maximal clicks in motoric trials; MaxMot). The overall HTCs and clicks showed ~~overall robust~~high reliability in both studies, within the original EEfRT in Study 1 (Rel =.90; Adj. Rel = .91) and in Study 2 (Rel. = .87), and within the modified EEfRT in Study 1 (Rel = .96; Adj Rel. = .92)

When separately analyzing the data for each probability and reward magnitude category, split-half reliabilities ranged between Rel = .73 and .97 over both studies and task versions. However, when calculating the split-half reliability for the difference scores, some indices showed relatively poor reliability (range: ~~d between~~ Rel. = .35 to~~and~~ .86), especially the difference between high and medium reward magnitudes. Overall, both versions of the EEfRT showed good reliability and the adjustment for motoric abilities in Study 1 impacted the reliability of the EEfRT only slightly.

Formatted: Level 1, Indent: First line: 0.49"

Table 2. Internal consistencies and reliabilities for questionnaires and both versions of the EEfRT in Study 1 and 2.

	Study 1	Study 2
Questionnaires		
BAS	α = 0.79	α = 0.74
BAS Fun Seeking	α = 0.67	α = 0.56
BAS Reward Responsiveness	α = 0.70	α = 0.53
BAS Drive	α = 0.74	α = 0.75
BIS	α = 0.78	α = 0.79
TEPS Anticipatory	α = 0.67	-
TEPS Consummatory	α = 0.70	-
UPPS Urgency	α = 0.86	α = 0.84
UPPS Premeditation	α = 0.78	α = 0.77
UPPS Perseverance	α = 0.83	α = 0.82
UPPS Sensation Seeking	α = 0.84	α = 0.83
Original EEfRT task		
HTCs	Rel =.90 / Rel_{adj} =.91	Rel =.87
Low Probability HTCs	Rel =.84 / Rel_{adj} =.84	Rel =.81
Medium Probability HTCs	Rel =.79 / Rel_{adj} =.78	Rel =.80
High Probability HTCs	Rel =.77 / Rel_{adj} =.76	Rel =.73
Low Reward HTCs	Rel =.82 / Rel_{adj} =.83	Rel =.80
Medium Reward HTCs	Rel =.82 / Rel_{adj} =.81	Rel =.78
High Reward HTCs	Rel =.82 / Rel_{adj} =.82	Rel =.79
New EEfRT task		
Clicks	Rel =.96 / Rel_{adj} =.92	-
Low Probability Clicks	Rel =.91 / Rel_{adj} =.87	-
Medium Probability Clicks	Rel =.96 / Rel_{adj} =.91	-
High Probability Clicks	Rel =.97 / Rel_{adj} =.93	-
Reward 1 Cent Clicks	Rel =.88 / Rel_{adj} =.84	-
Reward 2 Cent Clicks	Rel =.90 / Rel_{adj} =.82	-
Reward 3 Cent Clicks	Rel =.95 / Rel_{adj} =.89	-
Reward 4 Cent Clicks	Rel =.93 / Rel_{adj} =.84	-
Reward 5 Cent Clicks	Rel =.95 / Rel_{adj} =.88	-

	Study 1	Study 2
Questionnaires		
BAS	α = 0.79	α = 0.74
BIS	α = 0.78	α = 0.79
TEPS Anticipatory	α = 0.67	-
TEPS Consummatory	α = 0.70	-
UPPS Urgency	α = 0.86	α = 0.84
UPPS Premeditation	α = 0.78	α = 0.77
UPPS Perseverance	α = 0.83	α = 0.82
UPPS Sensation Seeking	α = 0.84	α = 0.83

Formatted: Font: Bold, Not Italic

Formatted: Font: Bold

Formatted Table

Formatted: Font: 12 pt

Formatted: Font: 12 pt

Original EEfRT task		
HTCs	Rel =.90 / Rel_{adj} =.91	Rel =.87
Low Probability HTCs	Rel =.84 / Rel_{adj} =.84	Rel =.81
Medium Probability HTCs	Rel =.79 / Rel_{adj} =.78	Rel =.80
High Probability HTCs	Rel =.77 / Rel_{adj} =.76	Rel =.73
High – Low Probability HTCs	Rel =.76 / Rel_{adj} =.76	Rel =.75
High – Medium Probability HTCs	Rel =.67 / Rel_{adj} =.66	Rel =.57
Low Reward HTCs	Rel =.82 / Rel_{adj} =.83	Rel =.80
Medium Reward HTCs	Rel =.82 / Rel_{adj} =.81	Rel =.78
High Reward HTCs	Rel =.82 / Rel_{adj} =.82	Rel =.79
High – Low Reward HTCs	Rel =.67 / Rel_{adj} =.67	Rel =.66
High – Medium Reward HTCs	Rel =.39 / Rel_{adj} =.38	Rel =.40
Modified~~New~~ EEfRT task		
Clicks	Rel =.96 / Rel_{adj} =.92	-
Low Probability Clicks	Rel =.91 / Rel_{adj} =.87	-
Medium Probability Clicks	Rel =.96 / Rel_{adj} =.91	-
High Probability Clicks	Rel =.97 / Rel_{adj} =.93	-
High – Low Probability Clicks	Rel =.86 / Rel_{adj} =.86	
High – Medium Probability Clicks	Rel =.68 / Rel_{adj} =.68	
Low Reward Clicks	Rel =.93 / Rel_{adj} =.89	-
Medium Reward Clicks	Rel =.95 / Rel_{adj} =.89	-
High Reward Clicks	Rel =.95 / Rel_{adj} =.89	-
High – Low Reward Clicks	Rel =.80 / Rel_{adj} =.80	-
High – Medium Reward Clicks	Rel =.37 / Rel_{adj} =.35	-

Note. Depicted are internal consistencies of questionnaire measures and split-half reliabilities of task measures. α = Cronbach‘s Alpha, Rel = split-half reliability calculated by Spearman-Brown correcting the split-half correlation of task measures (derived by splitting the dataset into the first and second temporal half of trials for each individual in the original task; calculated across the two blocks in the modified task), adj = adjusted for motor abilities: variables were predicted by the maximum number of clicks in motoric trials and residuals were used to calculate reliability, EEfRT = Effort Expenditure for Reward Task, HTC = Hard Task Choices.

Formatted: Space After: 8 pt, Widow/Orphan control, Hyphenate, Border: Top: (No border), Bottom: (No border), Left: (No border), Right: (No border), Between : (No border), Bar : (No border)

3.2 Experiment 1

3.2.1 Original EEfRT - Validity of Basic Task Variables

Participants on average chose the hard task in 53.41% of all trials (*SD* = 18.50%; Range = 2.90 – 100%). We conducted a GEE model to test validity of the basic task variables within the original EEfRT in Study 1 (see Table 3). The GEE model examined the main effects of task-dependent variables (reward magnitude, probability of reward attainment, and trial number) as well as one variable unique to our study (MaxMot) on the percentage of hard-task-choices. In line with previous studies, a significant positive main effect was found for reward magnitude and probability of reward attainment and a significant negative main effect was found for trial number, indicating that all three factors were predictors of percentage of hard-task choices (all *p*s < .001). Furthermore, the interaction of reward and probability (often referred to as “expected value”) did also reach significance, indicating that higher probability did predict a higher percentage of hard-task-choices with increasing reward magnitude (see Fig 3). The factor MaxMot did not reach ed also significance (Bβ = 0.002, $\chi^2(1)$ = 09.086, *p* = .772003), indicating that ~~participants with greater~~ motoric ability as measured within the motoric trials did -not strongly affectchose ~~the~~ hard task choices ~~more often~~ within the original EEfRT.

Fig 3. Percentage of hard-task-choices (HTC) within the original EEfRT in Study 1.

Comparison of trials with low probability of reward attainment (left; A), medium probability of reward attainment (middle; B) and high probability of reward attainment (right, C) within the original EEfRT. For each probability category all three categories of reward magnitude (low / medium / high) are displayed. Data points are added as dots for individual scores. Error bars depict a 95% confidence interval (CI) of the mean.

Formatted: Font: Not Italic

Formatted: Space After: 8 pt, Widow/Orphan control, Hyphenate, Border: Top: (No border), Bottom: (No border), Left: (No border), Right: (No border), Between : (No border), Bar : (No border)

Table 3. GEE Models for basic predictors of percentage of hard-task choices within the original EEfRT in Study 1 and 2 and of mean number of clicks within the modified EEfRT in Study 1.

Effect	*B*	*se*	χ^2	*p*
**Study 1 – Original EEfRT**				
Reward Magnitude	0.80	0.08	111.38	**<.001**
Probability 88%[a]	3.44	0.28	146.59	**<.001**
Probability 50%[a]	1.59	0.13	157.97	**<.001**
Probability 88%[a] × Reward Magnitude	1.32	0.20	44.46	**<.001**
Probability 50%[a] × Reward Magnitude	0.51	0.10	27.68	**<.001**
Trial	-0.77	0.12	41.81	**<.001**
MaxMot	0.00	0.01	0.08	.772
**Study 1 – Modified EEfRT**				
Reward Magnitude	4.91	0.46	111.80	**<.001**
Probability 88%[a]	17.80	1.53	135.98	**<.001**
Probability 50%[a]	12.47	1.07	135.35	**<.001**
Probability 88%[a] × Reward Magnitude	-1.83	0.43	18.51	**<.001**
Probability 50%[a] × Reward Magnitude	-1.33	0.38	12.19	**<.001**
Trial	-5.96	0.90	43.58	**<.001**
Block	-2.22	0.57	14.95	**<.001**
MaxMot	0.61	0.05	149.48	**<.001**
**Study 2 – Original EEfRT**				
Reward Magnitude	0.66	0.04	290.55	**<.001**
Probability 88%[a]	3.10	0.14	508.21	**<.001**
Probability 50%[a]	1.60	0.07	587.79	**<.001**
Probability 88%[a] × Reward Magnitude	0.91	0.10	90.63	**<.001**
Probability 50%[a] × Reward Magnitude	0.40	0.05	65.19	**<.001**
Trial	-0.72	0.07	103.37	**<.001**

Note. All models included probability (categorical), reward magnitude, and trial number (divided by the individual maximum number of trials) as within-subjects variables; χ² = Wald

chi-square; Bβ = regression coefficient; significant effects in **bold.** [a]Reference category: 12% probability

3.2.2 Modified EEfRT - Validity of Basic Task Variables

Participants on average exerted 117.,71 number of clicks in each trial (*SD* = 16.03; Range = 77.17 – 156.43). In accordance with our analysis of the original EEfRT (see 3.1.1), we computed a GEE model to test validity of the basic task variables within the modified EEfRT in Study 1 (see Table 3). The GEE Model examined main effects of task-dependent variables (reward magnitude, probability of reward attainment, and trial number) as well as one variable unique to our study (MaxMot) capturing the mean number of clicks. In line with previous studies, significant positive main effects were found for reward magnitude and probability of reward attainment and a significant negative main effect was found for trial number, indicating all three factors were predictors of the mean number of clicks (all *p*s < .001). Furthermore, the interaction of reward and probability also reached significance ($p <$ 0.001), indicating that higher probability predicted a stronger increase of mean number of clicks for smaller reward magnitudes (see Fig 4). The factor MaxMot also reached significance (Bβ = 0.61, $\chi^2(1) = 149.48$, p -< .001), indicating that participants with greater motoric ability as measured within the motoric trials exerted more clicks within the modified EEfRT.

Fig 4. Mean number of clicks within the modified EEfRT in Study 1.

Formatted: Font: Italic

Formatted: Font: Not Italic

Comparison of trials with low probability of reward attainment (left; **A**), medium probability of reward attainment (middle; **B**) and high probability of reward attainment (right; **C)** within the modified EEfRT. For each probability category all 5 different reward magnitudes (ranging from 1 Cent (most left) to 5 Cent (most right) are displayed. Data points are added as dots for individual scores. Error bars depict a 95% confidence interval (CI) of the mean.

3.3 Experiment 2

3.3.1 Original EEfRT - Validity of Basic Task Variables

Participants on average chose the hard task in 53.31% of all trials (*SD* = 19.21%; Range = 0 – 100%). We computed a GEE model to test the validity of basic task variables within the original EEfRT in Study 2 (see Table 3). The GEE Model examined main effects of task-dependent variables (reward magnitude, probability of reward attainment, and trial number) on the percentage of hard-task-choices. In line with previous studies and Study 1, significant positive main effects were found for reward magnitude and probability of reward attainment, and a significant negative main effect was found for trial number, indicating that all factors were predictors of the percentage of hard-task choices (all *p*s < .001). Furthermore, the interaction of reward and probability (often referred to as “expected value”) ~~did reach~~reached significance ($p < .001$), indicating that higher probability predicted ~~did predict~~ a higher percentage of hard-task-choices with increasing reward magnitude (see Fig 5).

Fig 5. Percentage of hard task choices (HTC) within the original EEfRT in Study 2.

Formatted: Font: Not Italic

Comparison of trials with low probability of reward attainment (left; A), medium probability of reward attainment (middle; B) and high probability of reward attainment (right; C) within the original EEfRT. For each probability category, all three reward magnitude categories (low / medium / high) are displayed. Data points are added as dots for individual scores. Error bars depict a 95% confidence interval (CI) of the mean

3.4 Personality correlations

To further validate the original and the modified EEfRT, we exploratorily correlated the percentage of hard-task-choices within the original EEfRT (Study 1 and Study 2) and the mean number of clicks within the modified EEfRT (Study 1) with personality traits as well as

the mean number of pumps in the BART (Study 1). We compared all trial categories as well as difference scores (see Table 4 and 5). Difference scores ~~indicate~~ reflect individual differences in the degree to which participants' hard task choices or clicks are influenced by the probability of reward attainment and the reward magnitude, respectively. For instance, p~~P~~ositive correlations between traits and difference scores would indicate that a higher ~~higher score within a specific trait~~trait value ~~increases the~~is associated with a stronger impact of probability of reward attainment or ~~the~~ reward magnitude on task performance.~~-~~ Surprisingly, trait BAS did not correlate significantly with the percentage of hard-task-choices in trials with low probability of reward attainment within the original EEfRT in both studies, nor with the mean number of clicks within the modified EEfRT in trials with low probability of reward attainment (see Fig 6). However, in the original EEfRT in Study 1, trait BAS correlated negatively with the difference score between trials with high probability of reward attainment and low probability of reward attainment. Conversely, trait BIS correlated positively with both difference scores (high probability minus low / medium probability trials). Trait anticipatory pleasure correlated positively with number of hard task choices within trials with low probability of reward attainment, as well as negatively with the difference score between trials with high probability of reward attainment and trials with low probability of reward attainment. Out of these correlations, only one was ~~two were~~ similar when comparing trials with different reward magnitudes. Trait anticipatory pleasure correlated positively with number of hard-task-choices within trials with low reward magnitude. Trait BIS correlated negatively with the difference score between trials with high reward magnitude and low reward magnitude Importantly, none of these correlations could be replicated in Study 2.

Note, however, that we did not administer the TEPS questionnaire and the BART in Study 2. The analyses for the modified EEfRT, which was administrated in Study 1 only, revealed a different pattern of results compared to the original EEfRT. Trait anticipatory pleasure correlated negatively with the difference score between trials with high probability of

reward attainment and trials with medium probability of reward attainment. Moreover, risk-taking behavior as measured via the BART correlated positively with the mean number of clicks in trials with medium and high probabilities of reward attainment. This~~ese~~ finding~~s~~ was~~ere~~ similar when examining reward magnitudes. Risk-taking behavior (BART) correlated positively with the mean number of clicks in trials with medium and high reward magnitudes. Across studies, tasks, and task parameters, the UPPS scales were largely unrelated to task performance.

Fig 6. Pearson correlations between trait BAS scores (z-standardized) and task performance (Study 1 and 2).

Correlations for the mean number of clicks within trials with low probability of reward attainment in the modified EEfRT (**A:** Study 1) and percentage of hard task choices within trials with low probability of reward attainment in the original EEfRT (**B**: Study 1; **C:** Study 2). All depicted correlations were nonsignificant with *p* >.05.

Formatted: Font: Not Italic

Table 4. Correlations between the original EEfRT (percentage of hard-task -choices), the modified EEfRT (mean number of clicks) and trait variables (Study 1 and 2).

**Trait variable**	**Reward Magnitude**				
**Original EEfRT (Study 1)**	**low**	**medium**	**high**	**high-low**	**high-medium**
BAS	.113	.055	-.055	-.159	-.147
BIS	.066	-.066	-.135	**-.180***	-.086
TEPS – anticipatory pleasure	**.235****	.178	.160	-.097	-.034
TEPS – consummatory pleasure	.061	.083	.023	-.041	-.083
UPPS-Urgency	-.131	-.072	-.021	.112	.070
UPPS-Premeditation	-.067	-.003	.069	.125	.094
UPPS-Perseverance	-.039	-.042	.090	.115	.175
UPPS-Sensation Seeking	.117	.107	.074	-.053	-.049
BART	.050	.115	.117	.050	-.004
**Original EEfRT (Study 2)**					
BAS	-.026	.006	.020	.046	.016
BIS	-.029	-.035	-.051	-.013	-.015
UPPS-Urgency	.009	-.009	-.025	-.032	-.019
UPPS-Premeditation	.017	.016	-.010	-.027	-.035
UPPS-Perseverance	-.007	.026	-.010	-.001	-.049
UPPS-Sensation Seeking	.036	.079	.092	.043	.005
**Modified EEfRT (Study 1)**					
BAS	.046	.006	-.006	-.089	-.044
BIS	.101	.082	.069	-.060	-.037
TEPS – anticipatory pleasure	.080	-.016	-.020	-.168	-.014
TEPS – consummatory pleasure	.069	.042	.062	-.018	.072
UPPS-Urgency	-.017	-.031	-.054	-.059	-.082
UPPS-Premeditation	-.060	-.051	-.041	.037	.032
UPPS-Perseverance	-.150	-.095	-.060	.159	.115
UPPS-Sensation Seeking	.024	.035	.055	.048	.071
BART	.154	**.224***	**.253****	.145	.120

Note. EEfRT = Effort Expenditure for Rewards Task; BAS = Behavioral Activation System Scale; BIS = Behavioral Inhibition System S~~s~~cale; TEPS: Temporal Experience of Pleasure Scale; UPPS = Urgency, Premeditation, Perseverance, and Sensation Seeking Impulsive Behavior Scale; BART = Balloon Analogue Risk Task.~~;~~ Correlations with (unadjusted) *p* <.05 are printed in bold. The significance level adjusted for multiple comparisons (*k* = 5 hypothesis tests for each correlate) was set to *p* < .01. * *p* < .05; ** *p* <.01 ~~significant effects~~

Formatted: Font: Bold, Not Italic

Formatted: Font: Bold

Formatted: Font: Italic

Formatted: Font: Italic

Formatted: Font: Italic

Formatted: Font: Italic

Formatted: Font: Italic

in **bold**. * p < .05; ** p <.01

Table 5. Correlations between the original EEfRT (percentage of hard-task -choices),

Trait variable	Reward Probability				
**Original EEfRT (Study 1)**	**12%**	**50%**	**88%**	**88-12%**	**88-50%**
BAS	.148	.018	-.073	**-.181***	-.074
BIS	-.114	-.105	.165	**.203***	**.238****
TEPS – anticipatory pleasure	**.291****	.164	.047	**-.243****	-.143
TEPS – consummatory pleasure	.154	-.007	-.010	-.149	.000
UPPS-Urgency	-.095	-.066	-.065	.050	.023
UPPS-Premeditation	.058	.016	-.131	-.131	-.116
UPPS-Perseverance	.029	-.039	-.006	-.031	.037
UPPS-Sensation Seeking	.115	.086	.095	-.051	-.023
BART	.021	.123	.148	.068	-.023
**Original EEfRT (Study 2)**					
BAS	-.024	-.022	.059	.060	.079
BIS	-.066	-.027	.004	.063	.036
UPPS-Urgency	-.035	-.015	.050	.064	.061
UPPS-Premeditation	.006	.023	-.017	-.017	-.043
UPPS-Perseverance	-.018	.051	-.026	.000	-.084
UPPS-Sensation Seeking	.025	.065	**.105***	.044	.015
**Modified EEfRT (Study 1)**					
BAS	.015	.045	-.007	-.024	-.123
BIS	.064	.108	.075	.001	-.071
TEPS – anticipatory pleasure	.075	.037	-.055	-.145	**-.222***
TEPS – consummatory pleasure	.049	.080	.048	-.010	-.072
UPPS-Urgency	.016	-.040	-.081	-.101	-.103
UPPS-Premeditation	-.032	-.036	-.080	-.044	-.111
UPPS-Perseverance	-.064	-.123	-.119	-.046	.000
UPPS-Sensation Seeking	.017	.041	.055	.036	.037
BART	.126	**.230***	**.249****	.106	.067

the modified EEfRT (mean number of clicks) and trait variables (Study 1 and 2)

Note. EEfRT = Effort Expenditure for Rewards Task; BAS = Behavioral Activation System Scale; BIS = Behavioral Inhibition System Sscale; TEPS: Temporal Experience of Pleasure Scale; UPPS = Urgency, Premeditation, Perseverance, and Sensation

Seeking Impulsive Behavior Scale; BART = Balloon Analogue Risk Task; significant effects in **bold**. Correlations with

Formatted: Font: Border: : (No border)

Formatted: Font: Bold, Not Italic

Formatted: Font: Bold

Formatted: Font: 9 pt

Formatted: Font: 9 pt

Formatted: Font: 9 pt

Formatted: Font: 9 pt

Formatted: Font: 9 pt

Formatted: Font: 9 pt

(unadjusted) *p* <.05 are printed in bold. The significance level adjusted for multiple comparisons (*k* = 5 hypothesis tests for each correlate) was set to *p* < .01. * *p* < .05; ** *p* <.01 ~~* *p* < .05; ** *p* <.01~~

3.5 Secondary Analyses

As Study 1 is one of the first studies to test two versions of the EEfRT within one experimental design, we further evaluated the validity of both tasks by exploratorily correlating the main dependent variables of both task versions within all three different probability of reward attainment categories (low: 12% / medium: 50% / high: 88%; see Fig 7) and all three different reward magnitude categories (low / medium / high). The correlations between the ~~complementary~~~~matcing~~matching dependent variables of both tasks were significant for all probabilities of reward attainment (*r* ranging from .192 - .305). Furthermore, the correlation between the ~~complementary~~matching dependent variables of both tasks was significant for trials with medium (*r* = .277) and high reward magnitudes (*r* = .218). Note that some discriminant correlations of non-matching reward probabilities or reward magnitudes exceeded the respective convergent correlations. The~~se~~ results indicate an overall linkage ~~(albeit only small~~~~moderate in size)~~ between performance on both task versions (albeit only small to moderate in size considering that the same construct should be measured).~~, as~~ S~~s~~ubjects who choose the hard task more often on the original EEfRT also exert more clicks within the modified EEfRT (see T~~t~~able 6 ~~for a complete overview of correlations~~).

Additionally, we asked participants to self-evaluate aspects that might have influenced their effort allocation individually for both task versions and asked them about their motivation to win money throughout the whole study. We then exploratorily correlated theses evaluations to their actual effort allocation in Study 1 comparing different trial categories and difference scores (see Table 7~~6~~ and 8~~7~~).

In line with our GEE analysis, which indicated probability of reward attainment ~~and~~

Formatted: Font: Italic

Formatted: Font: Italic

Formatted: Font: Italic

Formatted: Indent: First line: 0.49"

reward magnitude to be strongly connected to actual task performance, participants` self-evaluated importance of thesise two factors for their task performance correlated moderately to strongly with various trial categories in both task versions of the EEfRT. A comparable pattern was found for the modified EEfRT (see Table 76 and 87). The self-evaluated importance of reward magnitude was less strongly associated with performance in both task versions, although some moderately sized correlations emerged. When correlating participants` self-evaluated importance of fatigue for their task performance throughout the task, only one significant effect was observed. The number of clicks within the modified EEfRT correlated positively with the difference score between trials with high probability of reward attainment and medium probability of reward attainment. When correlating participants` self-evaluated importance of resting their fingers for their performance throughout the modified EEfRT, this evaluation also correlated significantly with the difference score between trials with high probability of reward attainment and trials with medium probability of reward attainment, indicating that participants who tried to rest their fingers more strongly, did so more often on trials with medium probability of reward attainment. A comparable result pattern was found for the original EEfRT. The strategy to rest their fingers was especially negatively related to hard-task-choices in often used for trials with low reward probabilities and medium reward magnitudes. Finally, when correlating participants` motivation to win money throughout the whole study, the mean number of clicks within the modified EEfRT correlated significantly within trials with all probability levels, as well as with trials with medium and high reward magnitudes, indicating that participants with high motivation to win money performed more clicks in almost all trial categories (see Table 76 and 87). In contrast to the modified EEfRT, the percentage of hard-task-choices within the original EEfRT did not correlate with participants` motivation to win money in any trial category.

Fig 7. Pearson correlations between task performance~~es~~ in both tasks in Study 1.

Formatted: Font: Not Italic

Correlations between the percentage of hard-task-choices (HTC %) within the original EEfRT and the mean number of clicks within the modified EEfRT for **A:** trials with low probability of reward attainment (=12%), **B:** medium probability of reward attainment (=50%) and **C:** high probability of reward attainment (=88%). All depicted correlations were significant with p <.05

Table 6. Zero-order correlations between the original EEfRT (percentage of hard-task choices) and the modified EEfRT (mean number of clicks) for different probabilities of reward attainment and different reward magnitudes.

	**Reward Probability**				
**Task**	**Modified EEfRT**				
**Original EEfRT**	12%	50%	88%	88-12%	88-50%
12%	**.216***	.085	-.046	**-.302****	**-.313****
50%	**.340****	**.305****	**.221***	-.176	**-.180***
88%	.093	.140	**.192***	.086	.138
88-12%	-.146	.004	.156	**.331****	**.372****
88-50%	**-.300****	**-.227***	-.097	**.256****	**.299****
	**Reward Magnitude**				
**Task**	**Modified EEfRT**				
**Original EEfRT ~~Modified EEfRT~~**	low	medium	high	high-low	high-medium
low	.160	.043	.020	**-.242****	-.077
medium	**.324****	**.277****	**.276****	-.108	.022
high	**.215***	**.210***	**.218***	-.015	.047
high-low	.024	.137	.166	**.228***	.116
high-medium	-.162	-.104	-.092	.129	.032

Notes. EEfRT = Effort Expenditure for Rewards Task. Correlations with (unadjusted) $p < .05$ are printed in bold. The adjusted significance level was set to $p < .01$. * $p < .05$; ** $p < .01$

Formatted: Font: Bold, Not Italic

Formatted: Font: Bold

Formatted: Font: 11 pt, Not Italic

Formatted: Font: 11 pt

Table 7~~6~~. Zero-order correlations between the original EEfRT (percentage of hard-task -choices), the modified EEfRT (mean number of clicks) and follow-up questions concerning task strategy and motivation in Study 1 for different probabilities of reward

**Strategy and motivation**	**Reward Probability**				
**Original EEfRT**	**12%**	**50%**	**88%**	**88-12%**	**88-50%**
Reward	.118	**.219***	.041	-.086	**-.207***
Probability	**-.513****	**-.232***	.128	**.552****	**.348****
Fatigue	-.092	-.082	-.078	.040	.030
Resting fingers	**-.199***	-.156	-.093	.130	.100
Motivation to win money	-.085	-.002	.069	.120	.054
**Modified EEfRT**					
Reward	.140	.172	.155	-.007	-.026
Probability	**-.200***	.017	.144	**.382****	**.312****
Fatigue	-.122	-.055	.030	.175	**.204***
Resting fingers	-.171	-.140	-.053	.149	**.201***
Motivation to win money	**.192***	**.231***	**.238****	.015	.035

attainment.~~.~~

Notes. EEfRT = Effort Expenditure for Rewards Task. The following five-point Likert-scales ranging from "not at all" (1) to "a lot" (5) were administered separately for each task: Reward: "How much did the reward magnitudes influence you on the task?"; Probability: "How much did the probabilities of reward attainment influence you on the task?"; Fatigue: "How much did Fatigue influence you on the task?"; Resting Fingers: "How much did attempts to rest your fingers influence you on the task?". The last question (same response format) was asked only once (i.e., not separately for each task): Money: "How motivated were you to win money throughout the whole study?". Correlations with (unadjusted)

Formatted: Font: Bold, Not Italic
Formatted: Font: Bold
Formatted Table
Formatted: Normal (Web), Line spacing: Double
Formatted: Font: 11 pt
Formatted: Font: 11 pt
Formatted: Font: 11 pt, English (United States)

p <.05 are printed in bold. The significance level adjusted for multiple comparisons (*k* = 5 hypothesis tests for each correlate) was set to *p* < .01. * *p* < .05; ** *p* <.01

~~**bold**. * p < .05 ** p < .01~~

~~**Strategy and motivation**~~	~~**Reward Magnitude**~~				
~~**Original EEfRT**~~	~~**low**~~	~~**medium**~~	~~**high**~~	~~**high-low**~~	~~**high-medium**~~
~~Reward~~	~~.029~~	~~.153~~	~~**.284****~~	~~**.212***~~	~~.162~~
~~Probability~~	~~**-.204****~~	~~**-.243****~~	~~**-.389****~~	~~-.129~~	~~-.177~~
~~Fatigue~~	~~-.065~~	~~-.115~~	~~-.086~~	~~-.009~~	~~.045~~
~~Resting fingers~~	~~-.108~~	~~**-.231***~~	~~-.152~~	~~-.022~~	~~.117~~
~~Motivation to win money~~	~~-.002~~	~~-.024~~	~~-.052~~	~~-.043~~	~~-.035~~
~~**Modified EEfRT**~~					
~~Reward~~	~~.116~~	~~**.190***~~	~~**.196***~~	~~.118~~	~~.039~~
~~Probability~~	~~-.123~~	~~.020~~	~~.059~~	~~**.306****~~	~~.139~~
~~Fatigue~~	~~-.100~~	~~-.022~~	~~-.023~~	~~.134~~	~~-.006~~
~~Resting fingers~~	~~-.163~~	~~-.124~~	~~-.096~~	~~.123~~	~~.087~~
~~Motivation to win money~~	~~.177~~	~~**.253****~~	~~**.271****~~	~~.136~~	~~.088~~

Table 8~~7~~. Zero-order correlations between the original EEfRT (percentage of hard-task -choices), the modified EEfRT (mean number of clicks) and follow-up questions concerning task strategy and motivation in Study 1 for different reward magnitudes.

Formatted: Font: 11 pt

Formatted: Font: 11 pt, English (United States)

Formatted: Font: 11 pt, Font color: Black, English (United States)

Formatted: Font: Bold

Formatted Table

Formatted: Font: Bold, Not Italic

Formatted: Font: Bold

Formatted: Font: Bold

Notes. EEfRT = Effort Expenditure for Rewards Task. The following five-point Likert-scales ranging from “not at all” (1) to “a lot” (5) were administered separately for each task: Reward: “How much did the reward magnitudes influence you on the task?”; Probability: “How much did the probabilities of

**Strategy and motivation**	**Reward Magnitude**				
**Original EEfRT**	**low**	**medium**	**high**	**high-low**	**high-medium**
Reward	.029	.153	**.284****	**.212***	.162
Probability	**-.204***	**-.243****	**-.389****	-.129	-.177
Fatigue	-.065	-.115	-.086	-.009	.045
Resting fingers	-.108	**-.231***	-.152	-.022	.117
Motivation to win money	-.002	-.024	-.052	-.043	-.035
**Modified EEfRT**					
Reward	.116	**.190***	**.196***	.118	.039
Probability	-.123	.020	.059	**.306****	.139
Fatigue	-.100	-.022	-.023	.134	-.006
Resting fingers	-.163	-.124	-.096	.123	.087
Motivation to win money	.177	**.253****	**.271****	.136	.088

reward attainment influence you on the task?”; Fatigue: “How much did Fatigue influence you on the task?”; Resting Fingers: “How much did attempts to rest your fingers influence you on the task?”. The last question (same response format) was asked only once (i.e., not separately for each task): Money: “How motivated were you to win money throughout the whole study?”. Correlations with (unadjusted) *p* <.05 are printed in bold. The significance level adjusted for multiple comparisons (*k* = 5 hypothesis tests for each correlate) was set to *p* < .01. * *p* < .05; ** *p* <.01 ~~**bold**. * p < .05 ** p < .01~~

Formatted: Font: Italic

Formatted: Font: Italic

Formatted: Font: Italic

Formatted: Font: Italic

Formatted: Font: Italic

4.Discussion

In the present study, we aimed to (1) validate the original EEfRT [21] and a modified version of the EEfRT [24] as measures of approach motivation by directly comparing both versions within one experimental design (Study 1) and to replicate the reliability and validity of the original EEfRT within a large sample (Study 2). We further aimed to (2) test the correlations between self-reported personality traits and behavioral measurements for different trial categories and difference scores, as well as between self-reported strategy usage and~~,~~ motivation and task performance in an exploratory fashion. We will now discuss the implication of the current findings.

4.1 Reliability and Validity of the original and modified version of the EEfRT

Supporting the results of previous studies [14, 22, 24], both the original EEfRT and the modified EEfRT showed overall good split-half reliability, indicating that both versions are producing reliable results. In terms of validity, our results are mixed. The basic validity of both tasks as measures of reward-dependent approach motivation ~~got~~ received further support from the GEE models in both studies as the basic task variables are in line with previous studies. In particular, we replicated~~ing~~ the typical pattern of effects of reward magnitude, probability of reward attainment, and trial number on the mean number of clicks (modified EEfRT) and the percentage of hard-task-choices (original EEfRT)~~. It can be concluded that both studies replicated the basic validity of the EEfRT~~. Furthermore, we ~~were able to show~~ found that the two~~both~~ versions of the EEfRT ~~did correlate~~intercorrelated significantly within all three ~~complementary~~matching probability of reward attainment categories as well as in trials with medium and high reward magnitudes in Study 1. ~~, indicating~~ a ~~a meaningful conceptual linkage between both tasks.~~ However, these correlations ~~correlations range between small and medium size~~have to be considered small to moderate given that the same construct is supposed to be measured. Thus,~~, indicating several conceptual~~ differences between the two~~both~~ tasks ~~that~~ need to be considered and will be discussed below.

Regarding the relationship between the self-ascribedself-reported personality traits and behavioral task measures, our correlations in both studies showed only very weak support for such a link, at best. Only in Study 1, trait BAS and trait anticipatory pleasure correlated significantly with the percentage of hard task choices within the original EEfRT and with the mean number of clicks within the modified EEfRT, for some task parameters. However, in contrast to our expectations, the correlating task parameters were not fully consistent withthe same as in previous studies [23, 24]. Furthermore, trait BIS correlated negatively with task performance in the original EEfRT in Study 1, indicating that trait BIS moderated the dependence of hard-task choices on reward magnitude (negatively related to BIS) and probability (positively related to BIS). However, as we analyzed the correlations of trait BAS and trait BIS for the original EEfRT within a larger sample in Study 2, none of these correlations replicated. Furthermore, other traits measuredself-reported traits - especially impulsivity as measured via the UPPS as well as consummatory pleasure as measured via the TEPS - did not show any meaningful correlations withto the behavioral measures in either both studyies.

Overall, tThese findings raise further questions about the existence and magnitude of such links, supporting studies not replicating them [22, 25, 26]. In particular, some correlations in previous work as well as Study 1 may have been overestimated due to random sampling error. Significant correlations may be at least partly attributable to the large number of possible correlations between task parameters and self-report measures. Supporting this notion, when correcting for multiple testing, only some effects of trait anticipatory pleasure and BIS remained significant, indicating that some of our findings might be false positives. Analyzing the correlations between performance on the BART and both task versions of the EEfRT in Study 1 revealed unexpected correlations between the mean number of clicks within the modified EEfRT and the mean number of pumps within the BART, indicating that risk-taking behavior might have impacted task performance as well, although this requires

Formatted: Indent: First line: 0.49"

further replication.

Taken together, the results of our data show a very mixed pattern regarding the validity of the EEfRT. Therefore, our results support a multiply determined view on behavioral measurements [42]. ~~Putting our results into a broader context, they are well in line with other studies that~~ In line with this, previous studies indicate that effort allocation within the EEfRT can be manipulated by a wide range of factors, ranging from mood inductions [23] over~~and~~ neurophysiological manipulations [24, 28, 45] to the influence of reduced motivation [21], or the intake of caffeine [45]. So how does a person decide whether to increase ~~her~~ effort to potentially gain a greater monetary reward within the EEfRT? Our mixed pattern of results shows that there is ~~a~~ no simple answer to this question. Especially the impact of reward attributes hints at a complex pattern behind participants' decisions and at the importance of individual reward evaluation.

Reward-based decision making is not a uniform process, it can rather be described as a set of distinct cognitive processes, which together direct the evaluation of a reward and thus form a person`s decisions within a concrete situation. According to Orsini et al. [61], reward-based (or "value-based") decision-making is compriseds~~ds~~ of three phases: 1. Decision representation (different options are identified, as are the costs and benefits associated with each option) and option valuation (each option is also assessed in terms of its subjective value in the moment of the decision), 2. action selection and 3. outcome evaluation (the value of the outcome of a choice is compared with the expected value of that outcome). It is reasonable to assume that the evaluation of potential benefits and costs can differ greatly between participants~~ and goes beyond personality traits~~. Importantly, these individual differences may be insufficiently captured by typical personality trait questionnaires. A potentially important factor is the type of reward and how much a person values this reward. Real-life reward types include e.g., ~~i.e.~~ social [62], physical [63, 64], and recreational [65, 66] rewards and their valuation has been successfully differentiated via self-reports [67]. As stated above, Lopez-

Formatted: Indent: First line: 0.49"

Gamundi & Wardle [51] were able to show that participants ~~did choose~~chose the hard task more often within a modified version of the EEfRT using cognitive tasks (C-EEfRT), although participants described the modified version as more difficult. The cognitive challenge of the modified version might have been rewarding in itself (although the monetary reward magnitude was unchanged). These results indicate that “costs” and “benefits” within a task can also be related to properties of the task itself.

To reach a better understanding of the self-evaluated aspects which might have influenced participants decisions, we asked them a series of questions about their strategies and motivation at the end of Study 1. We were able to show that effort allocation on both task versions was impacted by the self-evaluated importance of probability of reward attainment and ~~by the~~ reward magnitude, indicating that participants ~~are~~ show some ~~well~~ awareness of the factors that impact their behavior. Surprisingly, participants’ self-evaluated motivation to win money throughout the whole study correlated positively only with the mean number of clicks within the modified EEfRT, in all three categories of probability of reward attainment as well as in trials with medium and high reward magnitudes. The percentage of hard-task-choices within the original ~~motivation~~ EEfRT was not correlated with~~to~~ this self-evaluated monetary motivation. These results indicate that the individual evaluation of “costs” and “benefits” differs between both versions of the EEfRT, and hints at a potentially better validity of the modified EEfRT.

~~On the same note~~Lastly, one should also consider the nature of the questionnaires which assess personality traits, like trait BAS [6,55], trait anticipatory pleasure [7] or trait impulsivity [56, 57]: These questionnaires ~~co~~consist of~~mprise of~~ questions about various different situations, most of them complex real-life situations. Linking those ~~accumulated~~ scales to behavior in one artificial experimental situation might be rather difficult. There are several potential reasons for the lack of consistent associations. These pertain to (1) the validity of the questionnaire measures (e.g., self-reported traits only reflect one aspect of

personality), (2) the validity of the tasks (e.g., factors other than approach motivation may substantially affect task performance), and (3) the similarity of the measured constructs. Regarding the last point, one issue that deserves particular attention is the breadth of global trait measures as compared to the narrowness of ~~-~~task ~~This does not implicate that the EEfRT is not able to measure approach motivation. But it indicates that~~ behavior in one laboratory situation. For instance, the EEfRT could be a valid measure of approach motivation in this circumscribed situation and nevertheless be too ~~(affected by multiple factors) could be too limited~~ narrow ~~to assess global personality traits.~~for the assessment of broad trait-like behavioral tendencies.

4.2 Limitations and future directions

Although we analyzed two rather large samples to test the reliability and validity of the original and the modified version of the EEfRT and our study is one of the first to directly compare two versions of the EEfRT within one experimental design, there are still some limitations to our study. First, although we tried to stick as close to the original version of the EEfRT as possible [21], there is still a noteworthy ~~small~~ adaption, which might have impacted participants` behavior in our “original” EEfRT strongly. The adaption is based on a study by Hughes et al. [46], who decided to pay participants a percentage of the virtually won money instead of paying participants the money which they have won on two random trials [21]. We followed this adaption, as we expected the non-random payment to increase participants` overall approach motivation. However, we did not expect this adaption to change the basic response pattern in any significant way, which is also supported by our results replicating the basic predictors (i.e., reward attributes). Nonetheless, as we stated in the introduction, many ~~smaller and larger~~ adaptions of the EEfRT have been ~~installed~~ used in various studies, ranging from reduced complexity by fixing the monetary rewards [37], or by removing trials with low probability of reward attainment [38] to the addition of “loose” – trials [52], or the addition of

Formatted: Indent: First line: 0.49"

a social component [47]. Thus, we cannot rule out that ~~even~~ our ~~small~~ modification might have caused a significant change in behavior within the original EEfRT. In this regard, our modification might have impacted participants' strategic behavior, as the random payment introduced by the original EEfRT [21] might~~should~~ reduce strategic task choices compared to our version of the EEfRT. Therefore, another direct comparison of these two task versions is needed in future studies.

Furthermore, although both task versions used in Study 1 correlated significantly, suggesting some ~~conceptual~~ overlap, the correlations ranging from r = .160 to .305 between ~~complementary~~matching variables ~~also~~ indicate that the variable~~(s)~~ captured by the two tasks also differ substantially. Fig 7 indicates that this might partly be a result of floor-, and ceiling-effects regarding the effort allocation within original EEfRT (especially in trials with low and high probability of reward attainment). Future studies should therefore always consider that any change made to the original EEfRT e.g., to make the task fit to the experimental setting could lead to substantial changes in behavior and should therefore carefully compare any new version of the EEfRT to the original version. Furthermore, the floor- and ceiling-effects could be a general problem of the original EEfRT – at least when testing young healthy participants. These effects should also be considered, for instance, when ~~when i.e.~~ comparing healthy participants to patients with impaired approach motivation. Second, although we were able to test the original EEfRT within a large sample in Study 2, the comparison of both studies is limited. As stated in the methods section, the original EEfRT was part of a larger genetic study in Study 2 and task administration took place approximately 4 hours after start of the testing session. It is reasonable to assume that completing the task after 4 hours of testing might have~~be~~ influenced ~~by~~ participants' fatigue or boredom. Study 1 on the other hand lasted for only one hour~~ at our lab~~, which might have led to less fatigue or boredom.

Third, both samples ~~comprised~~ consisted mainly of young healthy students and both samples were not balanced in terms of age or gender. ~~We would like to state that~~Thus, it remains an open question whether the results of our research can~~not~~ be generalized to broader populations. As~~lthough~~ the original EEfRT has demonstrated to be a sensitive tool for detecting reduced approach motivation within various clinical samples [32–38], we cannot rule out that the results we found regarding the reliability and validity of the EEfRT could show a different pattern for other populations, e.g., within clinical samples. Fourth, as we discussed above, the rather mixed results regarding the validity of the original and the modified EEfRT might be a result of correlating scales from self-reports summarizing a large set of real-life situations and behavioral measurements within one ~~computational~~ laboratory task. The EEfRT ~~comprises investing~~assesses the investment of physical effort for monetary rewards. Other forms of effort as well as other forms of rewards should be introduced and tested. Furthermore, we suggest that assessing approach motivation should not be limited to~~on~~ one specific “cost” and one specific “benefit”. ~~Authors should rather~~For instance, future work might use a range of tasks or a range of variations of the EEfRT and calculate scales comparable to questionnaires assessing personality traits. The EEfRT offers a solid foundation to probe other forms of “costs” (~~ei~~.ge., the C-EEfRT which uses cognitive ~~tasks~~costs) [51] and a variety of different “benefits” (~~ei~~.ge., food portions) [50].

4.3 Conclusion

Taken together, our findings provide additional support for the split-half reliability of the original and the modified version of the EEfRT. Furthermore, the correlations between both task versions provide evidence for some overlap a~~the conceptual linkage of~~between~~overlap of both task versions~~the two tasks. However, ~~as~~ these correlations have to be considered small to moderate given that the same construct was targeted.- Thus~~are rather small~~, these findings also ~~they also~~ hint at noteworthy differences between the two tasks.~~.~~ The results regarding the

validity of the tasks are mixed. While the basic predictors of both task versions replicated well in both studies and are also supported by participants' self-evaluated importance of these factors, we were not able to replicate previous findings linking trait BAS and trait anticipatory pleasure to effort allocation within both versions of the EEfRT as only some performance parameters in Study 1 correlated with self-reported~~to~~ personality traits. Study 2 with a larger sample did not reveal any correlation involving ~~between~~ trait BAS ~~and trait BIS~~ for the original EEfRT. Furthermore, self-evaluated motivation hints at a possible~~n~~ advantage of the modified EEfRT regarding its validity. Our results indicate a complex ~~interaction~~ interplay of personality traits, task properties, and~~,~~ reward attributes. ~~, and study design.~~ Furthermore, they highlight the importance of analyzing the reliability and validity of the EEfRT and any modification applied to the task.

Acknowledgements

This research did not receive any specific grant from funding agencies in the public, commercial, or not-for-profit sectors. There were no conflicts of interest. The studies and the corresponding hypotheses were not preregistered. Datasets and syntax will be made publicly available here: https://osf.io/35k2w/?view_only=1f315859028c471787a96e153f5ac43a. Furthermore, we would like to thank Karoline Rosenkranz for supporting our project by collecting the data of study 2.

Field Code Changed

5. References

1. Harmon-Jones E, Harmon-Jones C, Price TF. What is approach motivation? Emot Rev. 2013; 5(3): 291–295. doi: 10.1177/1754073913477509

Field Code Changed

2. Lazarus RS. Cognition and motivation in emotion. Am Psychol. 1991; 46(4): 352–367. doi: 10.1093/jcr/ucaa025 025
3. Lazarus RS. Progress on a cognitive-motivational-relational theory of emotion. Am Psychol. 1991; 46(8): 819–834. doi: 10.1037/0003-066X.46.8.819
4. Gray JA. Brain Systems that mediate both emotion and cognition. Cogn Emot. 1990; 4(3): 269–288. doi: 10.1080/02699939008410799
5. Corr PJ. Testing problems in JA Gray's personality theory: a commentary on Matthews and Gillard (1999). Pers Individ Dif. 2001; 30(2): 333–352. doi: 10.1016/S0191-8869(00)00028-3
6. Carver CS, White TL. Behavioral inhibition, behavioral activation, and affective responses to impending reward and punishment: The BIS/BAS scales. J Pers Soc Psycholog. 1994; 67(2): 319–319. doi: 10.1037/0022-3514.67.2.319
7. Gard DE, Gard MG, Kring AM, John OP. Anticipatory and consummatory components of the experience of pleasure: A scale development study. J Res Per. 2006; 40(6): 1086–1102. doi: 10.1037/abn0000244
8. Mischel W. Personality and assessment. New York: Wiley; 1968.
9. Mischel W. From personality and assessment (1968) to personality science, 2009. J Res Per. 2009; 43(2): 282–290. doi: 10.1016/j.jrp.2008.12.037
10. Dang J, King KM, & Inzlicht M. Why Are Self-Report and Behavioral Measures Weakly Correlated? Trends Cogn Sci. 2020; 24(4): 267–269. doi: 10.1016/j.tics.2020.01.007
11. Salamone JD, Cousins MS, Bucher S. Anhedonia or anergia? Effects of haloperidol and nucleus accumbens dopamine depletion on instrumental response selection in a T-maze cost/benefit procedure. Behav Brain Res. 1994; 65(2): 221–229. doi: 10.1016/0166-4328(94)90108-2

12. Cléry-Melin M-L, Schmidt L, Lafargue G, Baup N, Fossati P, Pessiglione M. Why don't you try harder? An investigation of effort production in major depression. PLoS One. 2011; 6(8): e23178. doi: 10.1371/journal.pone.0023178

Field Code Changed

13. Kurniawan IT, Seymour B, Talmi D, Yoshida W, Chater N, Dolan RJ. Choosing to make an effort: the role of striatum in signaling physical effort of a chosen action. J Neurophysiol. 2010; 104(1): 313–321. doi: 10.1152/jn.00027.2010

14. Reddy LF, Horan WP, Barch DM, Buchanan RW, Dunayevich E, Gold JM, et al. Effort-based decision-making paradigms for clinical trials in schizophrenia: Part 1—Psychometric characteristics of 5 paradigms. Schizophr Bull. 2015; 41(5): 1045–1054. doi: 10.1093/schbul/sbv089

15. Hartmann MN, Hager OM, Reimann AV, Chumbley JR, Kirschner M, Seifritz E, et al. Apathy but not diminished expression in schizophrenia is associated with discounting of monetary rewards by physical effort. Schizophr Bull. 2015; 41(2): 503–512. doi: 10.1093/schbul/sbu102

16. Hartmann MN, Hager OM, Tobler PN, Kaiser S. Parabolic discounting of monetary rewards by physical effort. Behav Processes. 2013; 100: 192–196. doi: 10.1016/j.beproc.2013.09.014

17. Klein-Flügge MC, Kennerley SW, Saraiva AC, Penny WD, Bestmann S. Behavioral modeling of human choices reveals dissociable effects of physical effort and temporal delay on reward devaluation. PLoS Comput Biol. 2015; 11(3): e1004116. doi: 0.1371/journal.pcbi.1004116

18. Hershenberg R, Satterthwaite TD, Daldal A, Katchmar N, Moore TM, Kable JW, et al. Diminished effort on a progressive ratio task in both unipolar and bipolar depression. J Affect Disord. 2016; 196: 97–100. doi: 10.1016/j.jad.2016.02.003

19. Lane SD, Cherek DR, Tcheremissine OV, Steinberg JL, & Sharon JL. Response perseveration and adaptation in heavy marijuana-smoking adolescents. Addict Behav. 2007; 32(5): 977–990. doi: 10.1016/j.addbeh.2006.07.007

20. Strauss GP, Whearty KM, Morra LF, Sullivan SK, Ossenfort KL, & Frost KH. Avolition in schizophrenia is associated with reduced willingness to expend effort for reward on a Progressive Ratio task. Schizophr Res. 2016; 170(1): 198–204. doi: 10.1016/j.schres.2015.12.006

21. Treadway MT, Buckholtz JW, Schwartzman AN, Lambert WE, Zald DH. Worth the 'EEfRT'? The effort expenditure for rewards task as an objective measure of motivation and anhedonia. PLoS One. 2009; 4(8): e6598. doi: 10.1371/journal.pone.0006598

22. Horan WP, Felice Reddy L, Barch DM, Buchanan RW, Dunayevich E, Gold JM, et al. Effort-based decision-making paradigms for clinical trials in schizophrenia: Part 2—External validity and correlates. Schizophr Bull. 2015; 41(5): 1055–1065. doi: 10.1093/schbul/sbv090

23. Geaney JT, Treadway MT, Smillie LD. Trait anticipatory pleasure predicts effort expenditure for reward. PLoS One. 2015; 10(6): e0131357. doi: 10.1371/journal.pone.0131357

24. Ohmann HA, Kuper N, Wacker J. A low dosage of the dopamine D2-receptor antagonist sulpiride affects effort allocation for reward regardless of trait extraversion. Personal Neurosci. 2020; 3: E7. doi: 10.1017/pen.2020.7

25. Anand D, Oehlberg KA, Treadway MT, Nusslock R. Effect of failure/success feedback and the moderating influence of personality on reward motivation. Cogn Emot. 2016; 30(3): 458–471. doi: 10.1080/02699931.2015.1013088

26. Kaack I, Chae J, Shadli SM, Hillman K. Exploring approach motivation: Correlating self-report, frontal asymmetry, and performance in the Effort Expenditure for Rewards Task. Cogn Affect Behav Neurosci. 2020; 20(6): 1234–1247. doi: 10.3758/s13415-020-00829-x

27. Smillie LD. What is reinforcement sensitivity? Neuroscience paradigms for approach-avoidance process theories of personality. Eur J Pers. 2008; 22(5): 359–384. doi: 10.1002/per.674

28. Ohmann HA, Kuper N, Wacker J. Left frontal anodal tDCS increases approach motivation depending on reward attributes. Neuropsychologia. 2018; 119: 417–423. doi: 10.1016/j.neuropsychologia.2018.09.002

29. Harmon-Jones E, Gable PA. On the role of asymmetric frontal cortical activity in approach and withdrawal motivation: An updated review of the evidence. Psychophysiol. 2017; 55(1): e12879. doi: 10.1111/psyp.12879

30. Rutherford HJV, Lindell AK. Thriving and surviving: Approach and avoidance motivation and lateralization. Emot Rev. 2011; 3(3): 333–343. doi: 10.1177/1754073911402392

31. Wardle MC, Treadway MT, Mayo LM, Zald DH, de Wit H. Amping up effort: Effects of d-amphetamine on human effort-based decision-making. J Neurosci. 2011; 31(46): 16597–16602. doi: 10.1523/JNEUROSCI.4387-11.2011

32. Fervaha G, Graff-Guerrero A, Zakzanis KK, Foussias G, Agid O, Remington G. Incentive motivation deficits in schizophrenia reflect effort computation impairments during cost-benefit decision-making. J Psychiatr Res. 2013; 47(11): 1590–1596. doi: 10.1016/j.jpsychires.2013.08.003

33. Barch DM, Treadway MT, Schoen N. Effort, anhedonia, and function in schizophrenia: Reduced effort allocation predicts amotivation and functional impairment. J Abnorm Psychol. 2014; 123(2): 387–397. doi: 10.1037/a0036299

34. McCarthy JM, Treadway MT, Bennett ME, Blanchard JJ. Inefficient effort allocation and negative symptoms in individuals with schizophrenia. Schizophr Res. 2016; 170(2-3): 278–284. doi: 10.1016/j.schres.2015.12.017

35. Chang WC, Chu AOK, Treadway MT, Strauss GP, Chan SKW, Lee EHM, et al. Effort-based decision-making impairment in patients with clinically-stabilized first-episode psychosis and its relationship with amotivation and psychosocial functioning. Eur Neuropsychopharmacol. 2019; 29(5): 629–642. doi: 10.1016/j.euroneuro.2019.03.006

36. Treadway MT, Bossaller NA, Shelton RC, Zald DH**.** Effort-based decision-making in major depressive disorder: A translational model of motivational anhedonia. J Abnorm Psychol. 2012; 121(3): 553–558. doi: 10.1037/a0028813

37. Yang XH, Huang J, Zhu CY, Wang YF, Cheung EF, Chan RC, et al. Motivational deficits in effort-based decision making in individuals with subsyndromal depression, first-episode and remitted depression patients. Psychiatry Res. 2014; 220(3): 874–882. doi: 10.1016/j.psychres.2014.08.056

38. Damiano CR, Aloi J, Treadway M, Bodfish JW, Dichter GS. Adults with autism spectrum disorders exhibit decreased sensitivity to reward parameters when making effort-based decisions. J Neurodev Disord. 2012; 4(1): 13. doi: 10.1186/1866-1955-4-13

39. Nguyen NH, Albert AB, Van Orman S, Forken P, Blatt SD, Fremont WP, et al. Effort valuation and psychopathology in children and adults. Psychol Med. 2019; 49(16): 2801–2807. doi: 10.1017/S0033291718003884

40. Hughes DM, Yates MJ, Morton EE, & Smillie LD. Asymmetric frontal cortical activity predicts effort expenditure for reward. Cogn Affect Behav Neurosci. 2015; 10(7): 1015–1019. doi: 10.1093/scan/nsu149

41. Huang J, Yang X-H, Lan Y, Zhu C-Y, Liu X-Q, Wang Y-F, et al. Neural substrates of the impaired effort expenditure decision making in schizophrenia. Neuropsychology. 2016; 30(6): 685–696. doi: 10.1037/neu0000284

42. Gignac GE, Szodorai ET. Effect size guidelines for individual differences researchers. Pers Individ Dif. 2016; 102: 74–78. doi: 10.1016/j.paid.2016.06.069

43. Open Science Collaboration. Estimating the reproducibility of psychological science. Science. 2015; 349(6251): aac4716. doi: 10.1126/science.aac4716

44. Kuper N, Käckenmester W, Wacker J. Resting Frontal EEG Asymmetry and Personality Traits: A Meta-analysis. Eur J Pers. 2019; 33(2): 154–175. doi: 10.1002/per.2197

45. Wardle MC, Treadway MT, De Wit H. Caffeine increases psychomotor performance on the effort expenditure for rewards task. Pharmacol Biochem Behav. 2012; 102(4): 526–531. doi: 10.1016/j.pbb.2012.06.016

46. Hughes DM, Yates MJ, Morton EE, Smillie LD. Asymmetric frontal cortical activity predicts effort expenditure for reward. Cogn Affect Behav Neurosci. 2015; 10(7): 1015–1019. doi: 10.1093/scan/nsu149

47. Gilman JM, Treadway MT, Curran MT, Calderon V, Evins AE. Effect of social influence on effort-allocation for monetary rewards. PLoS One. 2015; 10(5): e0126656. doi: 10.1371/journal.pone.0126656

48. Hughes JR, Budney AJ, Muellers SR, Lee DC, Callas PW, Sigmon SC, et al. Does tobacco abstinence decrease reward sensitivity? A human laboratory test. Nicotine Tob Res. 2017; 19(6): 677–685. doi: 10.1093/ntr/ntw204

49. Johnson SL, Swerdlow BA, Treadway M, Tharp JA, Carver CS. Willingness to Expend Effort Toward Reward and Extreme Ambitions in Bipolar I Disorder. Clin Psychol Sci. 2017; 5(6): 943–951. doi: 10.1177/2167702617718181

50. Racine SE, Horvath SA, Brassard SL, Benning SD. Effort expenditure for rewards task modified for food: A novel behavioral measure of willingness to work for food. Int J Eat Disord. 2019; 52(1): 71–78. doi: 10.1002/eat.22999

51. Lopez-Gamundi P, Wardle MC. The cognitive effort expenditure for rewards task (C-EEfRT): A novel measure of willingness to expend cognitive effort. Psychol Assess. 2018; 30(9): 1237–1248. doi: 10.1037/pas0000563

52. Byrne KA, Ghaiumy Anaraky R. Strive to win or not to lose? Age-related differences in framing effects on effort-based decision-making. J Gerontol B Psychol Sci Soc Sci. 2020; 75(10): 2095-2105. doi: 10.1093/geronb/gbz136

53. Liang KY, Beaty TH, Cohen BH. Application of odds ratio regression models for assessing familial aggregation from case-control studies. Am J Epidemiol. 1986; 124(4): 678–683. doi: 10.1093/oxfordjournals.aje.a114441

54. Zeger SL, Liang KY. Longitudinal data analysis for discrete and continuous outcomes. Biometrics. 1986; 42(1): 121–130. doi: 10.2307/2531248

55. Strobel A, Beauducel A, Debener S, Brocke B. Eine deutschsprachige Version des BIS/BAS-Fragebogens von Carver und White. Zeitschrift für Differentielle und Diagnostische Psychologie. 2001; 22(3): 216-227. doi: 10.1024//0170-1789.22.3.216

56. Whiteside SP, Lynam DR. The five factor model and impulsivity: Using a structural model of personality to understand impulsivity. Pers Individ Dif. 2001; 30(4): 669–689. doi: 10.1016/S0191-8869(00)00064-7

57. Schmidt RE, Gay P, D’Acremont M, Van Der Linden M. A German adaptation of the UPPS impulsive behavior scale: Psychometric properties and factor structure. Swiss J Psychol. 2008; 67(2): 107–112. doi: 10.1024/1421-0185.67.2.107

58. Lejuez CW, Richards JB, Read JP, Kahler CW, Ramsey SE, Stuart GL, et al. Evaluation of a behavioral measure of risk taking: The balloon analogue risk task (BART). J Exp Psychol Appl. 2002; 8(2): 75–84. doi: 10.1037/1076-898X.8.2.75

59. Pleskac TJ, Wallsten TS, Wang P, Lejuez CW. Development of an automatic response mode to improve the clinical utility of sequential risk-taking tasks. Exp Clin Psychopharmacol. 2008; 16(6): 555–564. doi: 10.1037/a0014245

60. Janssen T, Larsen H, Peeters M, Boendermaker WJ, Vollebergh WAM, Wiers RW. Do online assessed self-report and behavioral measures of impulsivity-related constructs predict onset of substance use in adolescents? Addict Behav Rep. 2015; 1: 12–18. doi: 10.1016/j.abrep.2015.01.002

61. Orsini CA, Hernandez CM, Bizon JL, Setlow B. Deconstructing value-based decision making via temporally selective manipulation of neural activity: Insights from rodent models. Cogn Affect Behav Neurosci. 2019; 19(3): 459–476. doi: 10.3758/s13415-018-00649-0

62. Forbes EE, Dahl RE. Research Review: altered reward function in adolescent depression: what, when and how? J Child Psychol Psychiatry. 2012; 53(1): 3–15. doi: 10.1111/j.1469-7610.2011.02477.x

63. Chapman LJ, Chapman JP, Raulin ML. Scales for physical and social anhedonia. J Abnorm Psychol. 1976; 85(4): 374–382. doi: 10.1037/0021-843X.85.4.374

64. Zhang H, Harris L, Split M, Troiani V, Olson IR. Anhedonia and individual differences in orbitofrontal cortex sulcogyral morphology. Hum Brain Mapp. 2016; 37(11): 3873–3881. doi: 10.1002/hbm.23282

Field Code Changed

65. Johnson SL, Fulford D, Carver CS. The double-edged sword of goal engagement: consequences of goal pursuit in bipolar disorder. Clin Psychol Psychother. 2012; 19(4): 352–362. doi: 10.1002/cpp.1801

66. Ryba MM, Hopko DR. Gender Differences in Depression: Assessing Mediational Effects of Overt Behaviors and Environmental Reward through Daily Diary Monitoring. Depress Res Treat. 2012; 2012: 865679. doi: 10.1155/2012/865679

67. Khazanov GK, Ruscio AM, Forbes CN. The Positive Valence Systems Scale: Development and Validation. Assessment. 2020; 27(5): 1045–1069. doi: 10.1177/1073191119869836

Fakultät für Psychologie und
Bewegungswissenschaft

Dr. Hanno Andreas Ohmann
Universität Hamburg
Von-Melle-Park 5
Raum 4006
20146 Hamburg, Germany

Email: hanno_ohmann@hotmail.com
Tel: +49 (0)40 - 42838-5406
Web: www.psychobiology.de

August 19, 2021

Subject: Submission of revised paper PONE-D-21-08512 (title: "Examining the reliability and validity of two versions of the Effort-Expenditure for Rewards Task (EEfRT)")

Dear Prof. Dr. De Pascalis,

Thank you for your email dated 02 June 2021 enclosing the reviewers' comments. We have carefully reviewed the comments and have revised the manuscript accordingly. Our responses are given in a point-by-point manner below. Changes to the manuscript are tracked. Please note that line numbers in our response refer to the new "Manuscript" file (as line numbering is messed up within the tracked changes document

We feel that the reviewers' comments were very helpful in further improving on the Ms and hope that the revised version is now suitable for publication and look forward to hearing from you.

Sincerely,

Dr. Hanno Andreas Ohmann

Fakultät für Psychologie und
Bewegungswissenschaft

Response to Reviewer 1:

Reviewer #1: This study represents an important, relatively large scale study of the psychometrics and convergent and discriminant validity of alternate forms of the EEfRT, a task that is often modified with surprisingly little examination of the effects of those modifications. This report is an important one for anyone using the EEfRT to consider, and I offer a variety of suggestions to strengthen its contribution to the literature.

Thank You! Please note that line numbers in our response refer to the new “Manuscript” file (as line numbering is messed up within the tracked changes document.

INTRODUCTION

In the Hypotheses, the authors note that the reliability of the EEfRT appears promising from three previous studies; however, it would be helpful to note that based on their table, this is test-retest reliability specifically.

Thank you for raising this important issue. We clarified the types of reliability measured in previous studies as mentioned in the hypothesis (p. 11, l. 176-179).

In the paragraph spanning pp. 11-12, what were the actual correlations in the cited studies between EEfRT behavior and self-report measures? Having those relayed in the text would allow the reader to gauge immediately whether the authors’ assertions about the expected effect size were appropriate. In general, it would be appropriate to expect some shrinkage from published effect sizes, especially with a larger sample (which then has a smaller critical r value for a particular alpha level). Furthermore, Study 2’s Participants description seems to assume a smaller critical correlation without it being introduced here.

We fully agree and added the actual correlations to that paragraph (p. 11-12, l. 193-198). Indeed, we also expected some shrinkage from the published effect sizes. The smaller critical correlation expected in study 2 is partly based on the discrepancy found between published correlations and the

correlations found in our own lab. We clarified this issue in the methods section of study 2 (p. 19-20, l. 402-406).

METHOD (with references largely confined to Study 1)
To what extent do the rather rigorous exclusion criteria match those used in other studies with healthy samples?

Our exclusion criteria within study 1 are in line with previous studies conducted within our lab (i.e., Ohmann et al., 2018; Ohmann et al., 2020) and we believe those criteria are commonly used in the literature. The stricter criteria within study 2 are due to the clinical setting and the various molecular genetic hypotheses which are not part of the reported project.

Would changing the distribution of money from a randomly sampled two trials to 5% of the total winnings on the EEfRT constitute a modification to the overall procedure?
See below.

Nice job of using randomized task order to prevent order effects from influencing behavior. Why was the BART always in the middle of the task order?

Both versions of the EEfRT demand rather high motor-intense reactions. Thus, the BART (without any motor-intense reaction) has been used in the middle to avoid motoric fatigue of the subjects throughout the whole study. We adjusted the methods section to point out this issue (p. 14, l. 258-260).

Though the authors cite a couple of studies for the BART's automatic response procedure, it would seem that version of the task would compromise the motoric response component of the BART that might make it most comparable to the EEfRT. To what extent is that modified version of the BART consistent with the motor-intensive original version that requires key presses for each pump, both in terms of scores and correlations with external variables in theoretically expected ways?

The reactions to the BART should be seen as a different kind of reaction compared to the EEfRT, as the "pumps" of the original BART are not made under time pressure and each reaction could lead to the loss of the earned money within the specific trial (Lejuez et al., 2002). Thus, the task is not intended to measure physical effort. Instead, the BART (especially the automatic response procedure we used) measures impulsivity and the willingness of taking risks – which is the reason why we used this task. In this regard the automatic response version appears to be related to other measures in comparable fashion as the original version (for a comparison of both versions see Pleskac et al., 2008).

In the paragraph describing the modified EEfRT, I now see how the awarding of 5% of the total money earned might constitute a substantial modification compared to awarding the money won on two randomly selected trials. When money is awarded as a function of the proportion of (hard) trials completed, strategic concerns are minimized (which was an integral part of Zald and Treadway's reasoning for designing the task that way; see the middle of the second to last paragraph in the section describing the EEfRT in Treadway et al., 2009). However, when a flat 5% of the winnings are awarded, strategy plays a substantial role in what kinds of trials a person might choose to play. This discrepancy in extra compensation paradigms deserves explication.

Thank you for pointing out this important matter! We added a more critical review on this issue in the discussion (p. 41, l. 832-848).

Just to clarify, the maximum number of button presses across each of the 10 motoric ability trials was used as the MaxMot variable? Was this variable computed separately for each of the two iterations of this task?

Yes, the maximum number of button presses across each of the 10 motoric ability trials was used as the MaxMot variable and it was computed separately for each iteration of this task. As both versions of the EEfRT demand motor-intense reactions, we couldn`t rule out any kind of motoric fatigue. Therefore, we added two iterations of the motoric ability task (one before each version of the EEfRT). However, the maximum number of button presses did not differ significantly between both iterations.

What response function was used in the GEEs? The analyses of hard task choices should use some kind of binomial-based response rather than one assuming a linear response.

Yes, we used a binomial-based response for the hard task choices. We added this information to the methods section (p. 18, l. 373-375).

The GEEs should not use an independent working correlation matrix; that will assume there are no correlations among cells, which is incorrect in a design featuring within-subjects factors. Treadway et al. (2009) used an unstructured correlation matrix to fully account for the vagaries of the relationships among cells, but one can argue that correlation matrix estimates too many parameters to be stable. An autoregressive or m-dependent matrix might be plausible, as might an exchangeable matrix if the authors really wanted to cut down on the parameters estimated. However, the dependence among observations should be accounted for in the working correlation matrix to ensure reasonable standard errors. The marginal means are consistent even if a working correlations is misspecified, but the standard errors are not (e.g., Hin & Wang, 2009). However, the overall standard errors are not so extreme as to vitiate finding meaningful results in this study (per Table 3).

GEEs provide unbiased estimates and appropriate robust standard errors even in the case that the working correlation matrix is misspecified. We-emphasize this in the text (p. 18, l. 368-372). In line with your suggestions, we now report results using an exchangeable correlation matrix (Table 3). Results using this correlation matrix were highly similar to our original results.

Did the authors ensure equal distributions of low, medium, and high reward magnitudes across original EEfRT trials based on their binnings presented in the Data Analysis section?

The distribution of low, medium and high reward magnitudes is equal across the binnings of the original EEfRT trials.

RESULTS

If the split half reliability was calculated for the EEfRT's reliability, Cronbach's alpha (which is the average of all possible split halves) could also be calculated unless the halves were carefully balanced in some way. However, this is a relatively minor point.

Thank you for this suggestion. However, we intentionally chose the implemented split-half correlation over a measure of internal consistency. In particular, reliability was calculated by splitting by trial number (original task, with varying trial numbers per person) and block (modified task), yielding balanced test halves. We adjusted the method section to increase precision (p. 18, l. 363-365). Furthermore, we adjusted the report of reliabilities to show all relevant variables (Table 2)

How did the authors adjust raw EEfRT scores in Study 1 for motoric activity? Did they analyze some sort of residualized task performance score?

Yes, we residualized the task performance of the EEfRT (p. 21, l. 450-454). Within the GEEs we added the motoric abilities as an additional factor (MaxMot) to directly access its impact on task performance.

If I recall correctly, the β column in Table 3 should actually be a B column, as those coefficients are unstandardized.

Thank you for making us aware of this, we replaced all betas with Bs accordingly.

Figures 3-5 were really nicely done as a way to examine each participant's data along with the measures of central tendency and variability.

Thank You!

In Table 4, what adjustment was made for families of multiple comparisons? Some adjustment needs to be made given the exploratory nature of these analyses, which would likely alter the presentation of the results on p. 27. To maintain the presentation, it might be reasonable to use one tabular asterisk for an uncorrected $p < .05$ and two tabular asterisks for a corrected $p < .05$.

Thank you for this suggestion. We implemented the suggested approach in Tables 4, 5, 6, 7, and 8. In particular, we Bonferroni-corrected the alpha level for each correlate (trait or strategy / motivation) separately (5 correlates each), yielding an adjusted alpha level of .01. Accordingly, we note in the respective table notes that * indicates $p <.05$ at the uncorrected significance level and ** indicates $p <.01$, corresponding to $p <.05$ at an alpha level corrected for multiple comparisons for each correlate.

In the first paragraph of the Secondary Analyses section, I would argue that these correlations are even moderate given that they ostensibly represent alternate versions of the same task. I also did not see the values of the correlations for the various reward magnitudes, only the reward probabilities reported in Figure 7 (which is a lovely visualization of the relevant data).

We agree and added an additional table (Table 6) containing all relevant correlations between task parameters of the two tasks.

Tables 6 and 7 need corrections for multiple comparisons as well.

Thank you for making us aware of this, we implemented this correction as described above.

DISCUSSION

I believe the authors oversell substantially the comparability of the two versions of the EEfRT. Considerations of effect size are paramount in the first paragraph (e.g., difference between each form's internal consistency and alternate forms' correlations).
We adjusted the discussion and added a more critical view on this issue (p. 37, l. 741-746).

The discussion of self-report correlations may be a discussion of false positive errors given the lack of replicability of most of them across Studies 1 and 2 and the lack of corrections for multiple comparisons. The authors nod to this near the end of the second paragraph of the Discussion's first section, but they can check how well their findings would survive correction for multiple comparisons.
We adjusted the discussion and added a more critical view on this issue based on the correction for multiple testing (p. 38, l. 761-767).

I can appreciate how the authors contextualize the size of their adaptation of extra compensation allocation compared to other EEfRT adaptations. However, I think they may underestimate how that changes the (lack of) strategic considerations in the original EEfRT, which may explain some of the low correlations across EEfRT forms.
We adjusted the discussion and added a more critical view on this issue (p. 41, l. 832-848).

Good note that floor and ceiling effects in the original EEfRT might limit the size of the validity coefficients with the modified EEfRT.

Thank You!

MINOR POINTS

Many paragraphs span multiple pages. These must be broken so that they occupy ½ to ¾ of a single manuscript page so they contain a single idea and its elaborations.

We tried to break paragraphs wherever possible and hope that the Ms is now easier to read.

REFERENCES FOR THIS REVIEW

Hin, L.-Y., & Wang, Y.-G. (2009). Working-correlation-structure identification in generalized estimating equations. Statistics in Medicine, 28, 642-658. https://doi.org/10.1002/sim.3489

References for this response

Lejuez CW, Richards JB, Read JP, Kahler CW, Ramsey SE, Stuart GL, et al. Evaluation of a behavioral measure of risk taking: The balloon analogue risk task (BART). J Exp Psychol Appl. 2002; 8(2): 75–84. doi: 10.1037/1076-898X.8.2.75

Ohmann HA, Kuper N, Wacker J. Left frontal anodal tDCS increases approach motivation depending on reward attributes. Neuropsychologia. 2018; 119: 417–423. doi: 10.1016/j.neuropsychologia.2018.09.002

Ohmann HA, Kuper N, Wacker J. A low dosage of the dopamine D2-receptor antagonist sulpiride affects effort allocation for reward regardless of trait extraversion. Personal Neurosci. 2020; 3: E7. doi: 10.1017/pen.2020.7

Pleskac TJ, Wallsten TS, Wang P, Lejuez CW. Development of an automatic response mode to improve the clinical utility of sequential risk-taking tasks. Exp Clin Psychopharmacol. 2008; 16(6): 555–564. doi: 10.1037/a0014245

Response to Reviewer 2:

Reviewer #2: Generally a well done paper. It is a useful set of results about a fairly widely used measure. It is also quite informative to show that two versions of what is ostensibly the same measure, may actually have fairly modest correlations.

Thank you! Please note that line numbers in our response refer to the new “Manuscript” file (as line numbering is messed up within the tracked changes document.

In the discussion, I think the authors should explicitly mention all the personality traits that are not related to EEfRT, such as the UPPS. Although they do have some discussion of what it means that the behavioral measure is not really related to some important reward related measures of depression, I also think that they should provide more discussion of what it means that this behavioral measure is only minimally correlated with personality variables that have been shown to be related to depression, which the EEfRT is claimed to help measure.

We agree and now explicitly mention these traits in the discussion (p. 38, l. 755-760). Furthermore, we expanded the discussion of the minimal correlations between behavioral measures and

personality variables: We think that these correlations are still too exploratory in nature to be interpreted in terms of measurement of depression (via the EEfRT) and there are too many other possible factors impacting these correlations (p. 40, l. 814-826).

I have a number of minor comments.

As they note, the measure was developed for use in distinguishing clinical samples from normals, so it might be problematic to only collect data on a young, healthy sample. They do note at one point, the problem they had with ceiling and floor effects. This might be a general problem with the EEfRT.

Thank you for mentioning this issue. We adjusted the discussion (p. 42, l. 857-860).

They aren't always as explicit as they should be in stating that some of their measures, such as the TEPS and the UPPS are essentially not correlated with the EEfRT in a normal sample.

We adjusted the discussion as mentioned above (p. 38, l. 755-760).

Line 49. Culminated would be a better word choice than accumulated.

We corrected the term, thank you.

Line 75. Specifically would be a better word choice than Precisely.

We corrected the term, thank you.

Line 86. Say that one would expect relatively specific associations, but then don't give any idea of what those are.

We added an explanation to this section (p. 4-5, l. 89-92).

Line 108. "During task performance", not "while task performance"

We corrected the term, thank you.

Line 279. "Then, after a ready – screen (1s, C) main screen for the trial showing a…" There is punctuation or words missing. Doesn't' read properly.

We increased the precision of the task description (p. 15, l. 289-291).

Line 324 starting with "Furthermore, we…" Something is wrong with this sentence.

We corrected that sentence (p. 17, l. 334).

Line 387. "criterion" not "criterium"

We corrected the term, thank you.

Line 550. A correlation with a difference score is hard to interpret. Would be nice to explicitly state what it means.

We added an explanation to that section (p. 28 l. 576-579).

Line 560. So basically almost none of the personality variables are correlated with either version of the EEfRT in a normal sample?

That's correct. Overall, we increased precision of our manuscript regarding this issue according to your valuable input!

Data Review URL

Click here to download Data Review URL

http://osf.io/35k2w/?view_only=1f315859028c471787a96e153f5ac43a
