# Supplementary material for: Examining the reliability and validity of two versions of the Effort-Expenditure for Rewards Task (EEfRT)
Source: PLoS One. 2022 Jan 28;17(1):e0262902. doi: 10.1371/journal.pone.0262902 (PMC8797221; doi:10.1371/journal.pone.0262902)
Supplement: Attachment — Submitted filename: Response to Reviewers.docx [file pone.0262902.s003.docx]

**Dr. Hanno Andreas Ohmann**

Universität Hamburg

Von-Melle-Park 5

Raum 4006

20146 Hamburg, Germany

Email: hanno_ohmann@hotmail.com

Tel: +49 (0)40 - 42838-5406

Web: www.psychobiology.de

December 20, 2021

Subject: Submission of revised paper PONE-D-21-08512 (title: “Examining the reliability and validity of two versions of the Effort-Expenditure for Rewards Task (EEfRT)”)

Dear Prof. Dr. De Pascalis,

Thank you for your email dated 29 November 2021 enclosing the reviewers’ comments. We have carefully reviewed the comments and have revised the manuscript accordingly. Our responses are given in a point-by-point manner below. Changes to the manuscript are tracked. Please note that line numbers in our response refer to the new “Manuscript” file (as line numbering is messed up within the tracked changes document).

We feel that the reviewers’ comments were very helpful in further improving on the Ms and hope that the revised version is now suitable for publication and look forward to hearing from you.

Sincerely,

Dr. Hanno Andreas Ohmann

**Response to Reviewer 1:**

Reviewer #1: Overall, the authors were nicely responsive to the critiques raised in the original manuscript. I have only two minor interpretive points remaining in considering the GEE results in Study 1 (as reported in Table 3) that deserve further interpretation. I note it is possible the authors did so already and I just missed these points, but I didn’t find these interpretations on my reads of the revised and tracked-changes versions.

1) What does it mean that MaxMot did not influence the number of hard choices on the original EEfRT but was positively associated with the number of button presses in the modified EEfRT? In particular, I wonder if this pattern indicates the relative importance of basic motoric functioning as an influence on the primary outcome variable on the two versions of the task.

Our findings suggest that motoric abilities are more strongly related to the number of clicks in the modified EEfRT compared to hard task choices in the original EEfRT. These results indicate that both tasks also differ in terms of physical demands. Therefore, future work using this task should also include a measure of motoric abilities. We adjusted the discussion accordingly (p. 42, 856-868).

2) What does it mean that the Probability x Reward Magnitude interactions were opposite in sign between the original and modified EEfRT? The two outcomes are coded in the same direction, so the differences in these signs point to another potentially important difference in the two versions of the tasks – and which version is more theoretically compatible with the EEfRT’s aims?

Our findings suggest a different interplay of probability of reward attainment and reward magnitude in the two versions of the EEfRT. In the original EEfRT, the effects of reward magnitude on hard task choices are strongest for higher probabilities of reward attainment. In contrast, in the modified EEfRT, effects of reward magnitude on the number of clicks are largest for smaller probabilities of reward attainment. These differential patterns could be attributable to (1) strategy use in the original EEfRT stemming from the fact that hard task choices for low probability trials may lead to less money won overall, and (2) limits to the additional clicks possible in the modified EEfRT for high probability trials (more effort is required for additional clicks when the number of clicks is already high). We adjusted the discussion accordingly (p. 42, 856-868)

**Response to Reviewer 2:**

Reviewer #2: I'm fine with the current version. They have addressed the concerns I raised. I did flag some missing words and a couple of other issues. But generally I think it is in good shape.

Thank you! we have incorporated your suggestions in the manuscript. for instance, we have (1) corrected several language issues, (2) made it more explicit that our modifications of the EEfRT (number of clicks vs. choice between two tasks) represents a substantial change (see p. 16, 317-318), and (3) discussed the differences between both task versions in a more detailed manner (see p. 42, l. 856-868)

One issue that still concerns me is that the samples for both studies were recruited from a psychologically healthy population, which could lead to serious restriction of range problems on all the measures, including the EEfRT. Also, the first sample is only 120 or so, which suggests that there will be very few people with elevated or clinical levels of depression.

We agree to your concerns, which we already discuss within our manuscript. We added the restriction of range you mentioned to our discussion (p. 43, l. 883-884).
